# Ammonia oxidation at pH 2.5 by a new gammaproteobacterial ammonia-oxidizing bacterium

**DOI:** 10.1038/s41396-020-00840-7

**Published:** 2020-12-10

**Authors:** Nunzia Picone, Arjan Pol, Rob Mesman, Maartje A. H. J. van Kessel, Geert Cremers, Antonie H. van Gelder, Theo A. van Alen, Mike S. M. Jetten, Sebastian Lücker, Huub J. M. Op den Camp

**Affiliations:** 1 Department of Microbiology, IWWR, Radboud University, Heyendaalseweg 135, NL-6525 AJ Nijmegen, The Netherlands; 2 Laboratory of Microbiology, Wageningen University and Research, Stippeneng 4, 6708 WE Wageningen, The Netherlands

**Keywords:** Microbiology, Environmental microbiology

## Abstract

Ammonia oxidation was considered impossible under highly acidic conditions, as the protonation of ammonia leads to decreased substrate availability and formation of toxic nitrogenous compounds. Recently, some studies described archaeal and bacterial ammonia oxidizers growing at pH as low as 4, while environmental studies observed nitrification at even lower pH values. In this work, we report on the discovery, cultivation, and physiological, genomic, and transcriptomic characterization of a novel gammaproteobacterial ammonia-oxidizing bacterium enriched via continuous bioreactor cultivation from an acidic air biofilter that was able to grow and oxidize ammonia at pH 2.5. This microorganism has a chemolithoautotrophic lifestyle, using ammonia as energy source. The observed growth rate on ammonia was 0.196 day^−1^, with a doubling time of 3.5 days. The strain also displayed ureolytic activity and cultivation with urea as ammonia source resulted in a growth rate of 0.104 day^−1^ and a doubling time of 6.7 days. A high ammonia affinity (*K*_*m*(app)_ = 147 ± 14 nM) and high tolerance to toxic nitric oxide could represent an adaptation to acidic environments. Electron microscopic analysis showed coccoid cell morphology with a large amount of intracytoplasmic membrane stacks, typical of gammaproteobacterial ammonia oxidizers. Furthermore, genome and transcriptome analysis showed the presence and expression of diagnostic genes for nitrifiers (*amoCAB*, *hao*, *nor*, *ure*, *cbbLS*), but no *nirK* was identified. Phylogenetic analysis revealed that this strain belonged to a novel bacterial genus, for which we propose the name “*Candidatus* Nitrosacidococcus tergens” sp. RJ19.

## Introduction

Anthropogenic activities like the excessive use of nitrogen-based fertilizers in agriculture cause increased nitrification rates, leading to nitrogen loss from the soils, but also acidification. Low pH values influence many chemical and biological processes taking place in the soil [1]. Nitrification itself is directly affected by acidification, as, due to protonation of ammonium (NH_4_^+^), less ammonia (NH_3_) is available as substrate for ammonia-oxidizing archaea (AOA) and bacteria (AOB). Ammonia-oxidizing microorganisms use ammonia monooxygenase (AMO) to convert NH_3_ to hydroxylamine (NH_2_OH) [2, 3], which then is oxidized to nitric oxide (NO) by hydroxylamine oxidoreductase (HAO) and further to nitrite (NO_2_^−^) by a yet unidentified enzyme [4]. The produced NO_2_^−^ is subsequently taken up by nitrite-oxidizing bacteria (NOB) and converted to nitrate (NO_3_^−^) [5, 6] using the nitrite oxidoreductase complex. Recently, the existence of complete nitrifiers within the genus *Nitrospira* was demonstrated, and it was found that these comammox bacteria encode all enzymes for the conversion of NH_3_ to NO_3_^−^ [7, 8]. Under acidic conditions, NO_2_^−^ is chemically decomposed to NO_3_^−^ and NO [9, 10], the latter being extremely toxic to the cell, causing an additional challenge besides limited substrate availability.

Except for the archaeal “*Candidatus* (*Ca*.) Nitrosotalea” species and the acid-tolerant bacterium “*Candidatus* (*Ca*.) Nitrosoglobus (Ng.) terrae” sp. TAO100 that grow at pH 4 and 5, respectively, NH_3_ oxidation usually takes place at pH > 5.5 [5, 11–14]. However, several reports documented nitrification in acidic environments [2, 5, 15, 16]. Different mechanisms have been proposed to explain this observation. First, micro-sites with higher pH values might form in soil. Additionally, AOB and NOB might grow in close physical association, which could promote the transfer of NO_2_^−^ and thus prevent the accumulation of toxic compounds [5, 17, 18]. Supporting these hypotheses, it was observed that the formation of biofilm and cell aggregates helped maintaining activity at low pH [5, 17]. Moreover, to circumvent decreased substrate availability, microorganisms could intracellularly hydrolyze urea and use the released NH_3_ for nitrification [13, 19, 20]. Urea is an important nitrogen source and is heavily utilized as fertilizer. In 2004, urea represented >50% of the total nitrogenous fertilizer in agriculture. Furthermore, it is used as animal feed, in manufacturing processes, and it is excreted by animals [21]. Some microorganisms, including ammonia oxidizers [11, 22], have the ability of metabolizing urea thanks to the nickel-dependent metalloenzyme urease, producing NH_3_ and bicarbonate that helps buffering the acidification caused by subsequent ammonia oxidation [23, 24]. However, the limited amount of cultured acidophilic NH_3_ oxidizers limits our knowledge about their lifestyle and adaptations to these adverse conditions. Therefore, the aim of this project was to enrich ammonia-oxidizing microorganisms growing under acidic conditions. Indeed, in this study we report on the successful cultivation of a novel chemolithoautotrophic ammonia-oxidizing bacterium obtained from the biofilm of a full-scale air bioscrubber from a pig farm. This bacterium was able to grow even at pH 2.5, representing the most acidophilic ammonia oxidizer reported to date. It could use ammonia and urea as energy source(s), showing a faster growth rate during growth on ammonia. Furthermore, it possessed a high affinity for NH_3_ and extreme tolerance to NO.

## Materials and methods

### Sampling

Samples were obtained from a biofilter unit of a pig farm located in Erp, the Netherlands, in May 2016. Biofilm was scraped off the filter material and diluted in water taken from the biofilter tank. The materials were transported in closed buckets at room temperature to Radboud University (Nijmegen, The Netherlands) and processed for analysis. For chemical analysis, a water sample from the biofilter tank was centrifuged (14,000 rpm, 5 min). Subsequently, the supernatant was used for pH determination (691 pH meter, Metrohm, Ionenstrasse, Switzerland). Pelleted biomass was stored at −20 °C for DNA extraction and fixed with 3% (v/v) paraformaldehyde (PFA) for fluorescence in situ hybridization (FISH) as described below.

### Batch cultivation and medium composition

Biomass was diluted in 100 ml of AOB medium, composed of 0.8 mM MgSO_4_·7H_2_O, 0.2 mM CaCl_2_·2H_2_O, 1 mM NaCl, and 70 mM NH_4_Cl. After these chemicals were dissolved, a 1 M solution of K_2_HPO_4_ was added to a final concentration of 1 mM. Chelated trace elements (nitrilotriacetic acid) were supplied at final concentrations of: 1.5 μM ZnSO_4_·7H_2_O, 1 μM CoCl_2_·6H_2_O, 5 μM MnCl_2_·4H_2_O, 1 μM CuSO_4_·5H_2_O, 0.9 μM NaMoO_4_·2H_2_O, 0.8 μM NiCl_2_·6H_2_O, 1.1 μM Na_2_SeO_4_·10H_2_O, 2.3 μM H_3_BO_4_, and 0.7 μM CeCl_3_·6H_2_O. Iron was added as a separate solution to a final concentration of 32.9 μM FeSO_4_. The pH of the medium was adjusted to 3.5 with 1 M H_2_SO_4_. Cultures were incubated at room temperature on a shaker (SM-30 Edmund Buhler GmbH, 100 motions per min). pH was checked regularly with a (691 pH meter, Metrohm, Ionenstrasse, Switzerland) and adjusted to 3.5 with 1 M KHCO_3_ when necessary.

### Cultivation conditions with ammonium

Biomass from an active batch culture was used to inoculate a continuous flow membrane bioreactor (Applikon Biotechnology B.V., Delft, The Netherlands) filled with 2 L of AOB medium at pH 3.5. The reactor was operated at room temperature with a stirring speed of 850 rpm and was equipped with pH and level sensors connected to an ADI 1010 biocontroller (Applikon Biotechnology B.V., Delft, The Netherlands). The controller automatically maintained the pH at 3.5 by dosing with sterile 1 M KHCO_3_ solution. Gas was regulated to supply 290 mL/min air and 4 mL/min CO_2_. After an initial batch phase of 20 days, the reactor was supplied with ~1 L/day of AOB medium pH 3.5 and biomass was removed at a rate of 0.2 L/day. The remaining 0.8 L/day of liquid was removed via the membrane filter. The exact volume of the effluent and consumption of KHCO_3_ were measured by analytical balances.

### Cultivation conditions with urea

50 ml of biomass from the reactor grown with NH_4_Cl was used to inoculate a new membrane bioreactor (Applikon Biotechnology B.V., Delft, The Netherlands) with a working volume of 1.5 L. The reactor was filled with AOB medium at pH 3.5 supplied with 50 mM urea instead of NH_4_Cl and with double the concentration of Trace element solution. The reactor was operated at room temperature and supplied with 0.8 L/day medium. pH and agitation (800 rpm) were regulated via an In-Control biocontroller (Applikon Biotechnology B.V., Delft, The Netherlands). pH was controlled at 3.5 by dosing 1 M KHCO_3_ or 1 M H_2_SO_4_ solutions. Gas flow was maintained at 300 ml/min air and 10 ml/min CO_2_. KHCO_3_ consumption and effluent volumes over the membrane were measured by analytical balances.

### Analytical methods

The concentration of urea was measured by converting urea to ammonia via the urease reaction. 200 µL of sample were incubated with 100 µL urease (2 mg/ml, Sigma-Aldrich) at 37 °C for 30 min. Ammonium was determined colorimetrically using the reaction of orthophatal-dialdehyde and mercaptoethanol with NH_4_^+^ salts at pH 7.4 [25].

NO and NO_2_ gases were measured using a Nitric Oxide Analyzer (CLD 700EL, Eco Physics).

For NO_2_^−^ and NO_3_^−^ determination, 1 ml of liquid sample was centrifuged at 14,000 rpm for 2 min, and the supernatant was diluted immediately in 400 mM phosphate buffer pH 7.4 to avoid chemical conversion of NO_2_^−^. NO_3_^−^ and NO_2_^−^ were measured on an ICS2100 Ion chromatography system (Thermo Scientific, Breda, The Netherlands), using a Dionex Ionpac AS16 column (250 × 2 mm) held at 30 °C. The eluent was a gradient generated from a potassium hydroxide cartridge, held at a flow of 0.4 ml/min. The detection was done via a suppressed conductivity detector.

The KHCO_3_ consumption in the reactor was measured daily using a balance. For the pH optimum experiment, a custom made measuring cylinder was connected to the reactor and the amount of KHCO_3_ solution (1 M) was registered every 15 min. During the measurements the reactor was kept in batch mode to prevent buffering from the medium supply. At each pH set point, values were consecutively tested in the reactor and results were reported as the average of three biological replicates. When changing pH value, the culture was adapted for a period of time (4–24 h) to the new pH set point before the start of the measurements.

### DNA extraction and sequencing

To obtain the metagenome of the acidic air scrubber, data retrieved from two sequencing runs performed with Ion Torrent next generation sequencing (Thermo Fisher Scientific Inc., Waltham, USA) and Illumina MiSeq (Illumina, San Diego, USA) were combined. For Illumina sequencing, genomic DNA was extracted from biofilm and water samples collected from the biofilter using the DNeasy Blood & Tissue Kit (Qiagen, Valencia, USA) according to manufacturer’s instructions. Sequencing libraries were prepared with the Nextera XT Library Preparation Kit (Illumina) in accordance with the manufacturer’s instructions. Libraries were checked for quality and size distribution using the Agilent 2100 Bioanalyzer and the High Sensitivity DNA kit (Agilent Technologies, Santa Clara, USA). Quantitation of the library was performed by Qubit using the Qubit dsDNA HS Assay Kit (Thermo Fisher Scientific Inc., Waltham, USA). Paired-end sequencing (2 × 300 bp) was done using the Illumina MiSeq sequence machine (Illumina) and the MiSeq Reagent Kit v3 (Illumina) according the manufacturer’s protocol. For Ion Torrent sequencing, DNA isolation was done by resuspending the sample in 0.75 ml 120 mM PBS. After addition of ~0.3 g glass beads (0.25 mm diameter), bead beating was performed using the Tissuelyser LT (Qiagen, Netherlands) at maximum speed for 1 min. Subsequently, DNA was extracted as described by Kowalchuk et al. [26]. DNA quality and concentration were determined by 0.7% agarose electrophoresis and Qubit using the dsDNA HS Assay Kit, respectively. For both samples 100 ng of DNA were used to prepare 400 base pair libraries for Ion Torrent sequencing according the manufacturer’s protocol. For sequencing, the libraries were subjected to clonal amplification using the Ion One TouchTM 2 Instrument and the Ion PGM™ Template OT2 400 Kit (Life Technologies, Carlsbad, USA) according to the manufacturer’s instructions. Sequencing was performed using the Ion PGM™ Sequencing 400 Kit and the Ion Torrent PGM using 212 cycles (850 flows) (Thermo Fisher Scientific, Waltham, USA). Sequencing reads were filtered using the PGM software to remove polyclonal and low quality sequences.

### Genome assembly and annotation

Ion Torrent sequencing resulted in 1,775,546 reads. Trimming was performed with CLC Genomics Workbench 7.5 (Qiagen Aarhus A/S, Denmark), resulting in 1,560,659 trimmed reads (quality limit 0.05, minimum size 100 bp). Illumina MiSeq sequencing resulted in 32,998,926 paired reads and after trimming (quality limit 0.01, minimum size 100 bp), 28,745,790 paired reads and 1,208,706 orphan reads were obtained.

All paired-end Illumina sequencing reads were assembled using CLC Genomics Workbench 7.5, resulting in 22,914 contigs, ranging in size from 1843 to 544,772 bp with an average coverage of 2.28× to 1879.40×. Contigs were manually checked by BlastN [27] and 11 of them, ranging from 2088 to 544,772 bp and 246.26× to 277.75× coverage, were selected for reassembly with Spades [28] (−k 21,33,55,77,99,121 –cov-cutoff auto). The reassembly included the 11 contigs and both the complete set of trimmed paired-end and orphan reads from the Illumina Miseq sequencing run, as well as the trimmed reads from Ion Torrent sequencing. This resulted in 118,612 contigs, ranging from 159 to 1,070,812 bp.

BlastN [27] was used to compare the sequences of the 11 contigs with the Spades assembly, resulting in 5 final contigs. These contigs were manually joined and checked by mapping all trimmed sequencing reads (full length, 100% similarity) to the completed genome. Finally, two repeated regions and the 16S rRNA genes were resolved by Sanger sequencing using the primers listed in Supplementary Table S1. The final closed genome of 1,811,369 bp was submitted to the MicroScope annotation platform [29, 30]. Genes of main metabolic pathways were manually annotated in MicroScope as described elsewhere [31].

### PCR amplification and cloning

The presence of “*Candidatus* (*Ca*.) Nitrosacidococcus (Na.) tergens RJ19” in DNA samples was checked by using the primer pairs 16Snitro2F/R and amoAnitro1F/R (Supplementary Table S1), which were designed to target the novel acidophilic ammonia oxidizer, together with members of the *Nitrosococcus* genus.

PCR experiments were performed in a final volume of 20 μL containing 10 μL PerfeCTa SYBR Green FastMix (Quantabio, Beverly, USA) and 1 μM of each primer (Supplementary Table S1). PCRs consisted of an initial denaturation at 96 °C for 5 min, followed by 35 cycles of 96 °C for 1 min, 49–60 °C for 1 min, and 72 °C for 30–60 s, and a final elongation at 72 °C for 10 min. Annealing temperatures used for the different primer pairs are listed in Supplementary Table S1. PCR amplicons were visualized by horizontal gel electrophoresis using 1% (w/v) agarose gels pre-stained with ethidium bromide and run in SB buffer for 20 min at 100 V. PCR products were purified using the PCR Purification Kit (Qiagen, Valencia, USA) following manufacturer’s instructions, ligated into the pGEM-T Easy TA cloning vector (Promega, Madison, USA) and transformed into *Escherichia coli* XL-1 Blue according to the manufacturers’ instructions. Transformants were screened using PCR with M13 primers; recombinant plasmids were extracted using GeneJET Plasmid Miniprep Kit (Thermo Fisher Scientific, Waltham, USA) and sequenced by Sanger sequencing (BaseClear B.V., Leiden, The Netherlands).

### Phylogenetic analysis

Representative reference 16S rRNA gene and ammonia monooxygenase subunit A (AmoA) sequences were identified in the NCBI *nr* database by BLAST, aligned with the sequences obtained in this study by MUSCLE [32], and used to build phylogenetic trees with the Maximum Likelihood method and 500 bootstraps in Mega 10.1.5 [33].

### RNA extraction and sequencing

Total RNA was extracted in triplicate using the RiboPure RNA Purification Kit (Thermo Fisher Scientific) according to the manufacturer’s protocol. Transcriptome libraries were constructed using the TruSeq Stranded mRNA Library Prep protocol (Illumina) according to the manufacturer’s instructions. Total RNA was used for library preparation, which was checked for quality using the Agilent 2100 Bioanalyzer (Santa Clara, USA). Library quantitation was performed by Qubit using the Qubit RNA BR Assay Kit (Thermo Fisher Scientific). Libraries were normalized, pooled, and sequenced using the Illumina MiSeq sequence machine. For sequencing, the 150 bp single-read sequencing chemistry was performed using the MiSeq Reagent Kit v3 (Illumina, San Diego, California, USA) according the manufacturer’s protocol. Raw reads were exported and analyzed in CLC Genomics Workbench (version 12, Qiagen Aarhus A/S, Denmark), trimmed and mapped against ncRNAs to discard rRNAs and tRNAs. Expression values were reported as RPKM (Reads Per Kilobase of transcript per Million mapped reads) [34]. Statistical analyses were performed using the DESeq2 package [35] in R Studio.

### Kinetics of ammonia oxidation

The NH_3_ affinity constant was determined using an oxygen microsensor (RC350, Strathkelvin, Motherwell, UK). The experiments were performed with concentrated biomass obtained as follows: 15 ml of culture from the bioreactor cultivated with NH_4_Cl was centrifuged for 10 min at 2000 rpm and washed twice in AOB medium pH 4.7 without substrate to remove any residual NH_4_^+^. Subsequently, the pellet was resuspended in 3 ml medium. After addition of 3 ml cell suspension to the measurement chamber, the cells were aerated for 5 min at room temperature, and the O_2_ signal was recorded using SensorTrace Basic software (Unisense, Aarhus, Denmark). The respiration chamber was kept at a constant temperature of 22 °C and stirred at 500 rpm. By injecting via a 100 µL syringe different volumes of a solution of NH_4_Cl, different ammonium concentrations were provided to the cell suspension and the resulting oxygen consumption was recorded for analysis. The experiment was carried out in triplicate for each concentration of NH_4_Cl and fresh biomass was used for every replicate. At the end of the experiment, cells were retrieved from the respiration chamber, centrifuged for 5 min at 4000 rpm and stored at −20 °C for protein determination.

Oxygen consumption data were analyzed in OriginPro 2018 (64-bit) b9.5.0.193 and fitted to a Michaelis–Menten type curve using nonlinear least-squares analysis, according to the equation *V* = *V*_max_ × [*S*]/(*K*_*m*_ + [*S*]) in which *V* was measured as oxygen consumption rate and [*S*] represents the ammonium concentration.

### Protein determination

Proteins were extracted by incubating pelleted biomass in 0.5 ml 0.5 M NaOH for 30 min at 90 °C, followed by neutralization by adding about one volume of 0.5 M HCl. For samples from the kinetics determination experiment, cell lysis was achieved by using the B-PER Bacterial Protein Extraction Reagent (Thermo Fisher Scientific) according to manufacturer’s instructions. Protein concentrations were measured with the BCA Protein Assay (Thermo Fisher Scientific) following the manufacturer’s protocol.

### Fluorescence in situ hybridization (FISH)

Biomass was fixed with 3% (v/v) PFA and hybridized with a newly designed Cy3-labeled oligonucleotide probe specific for the novel ammonia-oxidizing bacterium (Nater1117; 5′-CTAAATCGCTGGCAACTAA-3′) using 10% formamide, as described elsewhere [7]. For visualization, slides were embedded in Vectashield (Vector Laboratories Inc., Burlingame, CA) containing 4′,6-diamidino-2-phenylindole (DAPI), and fluorescence was recorded by a Zeiss Axioplan 2 microscope (Carl Zeiss AG, Oberkochen, Germany) equipped with an AxioCam (Zeiss) and processed using the AxioVision software (Version 4.8.2 SP3).

### Electron microscopy

4 ml of biomass was harvested from the membrane bioreactor and concentrated by centrifugation (400 × *g*, 4 min). The pellet was resuspended in 15 μL of supernatant, and subsequently samples of 0.6 μL were high-pressure frozen in a HPM-100 (Leica Microsystems) using gold-plated platelets (2 mm inner diameter, 100 μm sample thickness). Samples were freeze-substituted in an AFS2 (Leica Microsystems) using anhydrous acetone (Seccosolv, Merck, Darmstadt, Germany) containing 0.2% uranyl acetate (Merck). The substitution started at −90 °C for 48 h, followed by a +2 °C/h temperature increase to −70 °C, where the sample remained for 12 h. Afterwards, the temperature was raised by 2 °C/h to −50 °C, where the sample remained for 12 h. The sample was washed twice with anhydrous acetone at −50 °C and stepwise infiltrated with Lowicryl HM20 (Electron Microscopy Sciences, Hatfield PA, USA) in anhydrous acetone at −50 °C. After four changes of 100% HM20, the samples were polymerized by UV irradiation at −50 °C for 96 h. Next, the temperature was increased by 2 °C/h to 0 °C, where the sample was kept for 24 h. Ultrathin sections (ca. 50 nm) were cut using an ultramicrotome (Ultracut, Reichert-Jung, Vienna, Austria) and transferred to 100 mesh copper grids (Stork-Veco, Eerbeek, Netherlands) containing a carbon-coated formvar film. Micrographs were recorded in a JEOL Jem-1400 Flash transmission electron microscope (JEOL Ltd., Tokyo, Japan) operating at 120 kV.

## Results

### Description of the sampling site

The samples were obtained from a biological full-scale packed-bed scrubber that was treating exhaust air from a pig stable (Erp, The Netherlands). The exhaust air passed through a dust filter unit before entering the scrubber. The scrubber showed a stable performance at 18–19 °C and no acid was needed to maintain pH 2–3, indicating considerable microbial ammonia-oxidizing activity that is confirmed by scrubber performance (Supplementary Fig. S1). The washing water contained high NH_4_^+^ and NO_3_^−^ concentrations and NH_4_^+^ was removed at 99% efficiency (Supplementary Table S2). As expected, low concentrations of NO_2_^−^ were detected since this compound will be chemically converted into NO_3_^−^ and NO at low pH [9, 10]. The microbial nitrifying community inhabiting the full-scale air scrubber was characterized by metagenomic analysis. 6.4% of the 16S rRNA gene reads extracted from the full metagenome were affiliated with one novel gammaproteobacterial species distantly related to the genus *Nitrosococcus* (Supplementary Table S3). From this putative novel ammonia-oxidizing microorganism, we subsequently assembled the complete genome using a combination of high-throughput short read sequencing and manual, Sanger sequencing-based genome refinement.

### Genome description and phylogenetic analysis

The circular genome consisted of 1,811,369 bp and contained 1776 coding sequences (CDSs). Average CDS length was estimated at 929 bp, with a protein coding density of 90.3%. The G + C content was 37% and no plasmids were identified. *Ca*. Ng. terrae has a G + C content of 42%, while the obligately halophilic gamma AOB all have a GC content over 50%. The genome contained two full rRNA operons with 99.5%, 100%, and 100% 16S, 23S, and 5S rRNA identity to each other, respectively; in one of them (NSCAC_5s_rRNA_2–NSCAC_16s_rRNA_2) Ala and Ile tRNAs (NSCAC_tRNA30, 31) were encoded in between the 16S and 23S rRNA genes.

16S rRNA gene sequence-based phylogenetic analysis including searching the IMNGS platform (https://www.imngs.org/) (Supplementary Fig. 2A) revealed that the most closely related sequences were obtained from a refuse dump of leaf-cutter ants [36] (>99% identity), from a Finnish soil (PRJNA393827), and from a synthetic urine nitrification reactor [37], the closest cultured species was “*Ca*. Ng. terrae” [11]. The two species showed only 92% 16S rRNA gene identity (Supplementary Table S4), and an average nucleotide identity (ANI) [38, 39] of 76.2% (Supplementary Table S5). The AmoA sequences had 78% amino acid identity (Supplementary Fig. S2B and Table S6). ANI and 16S rRNA identity values fell far below the cut-off for species delimitation (95% for ANI and 98.7–99% for 16S rRNA) [38, 40–44], placing this nitrifier in a novel genus, for which we propose the name of “*Ca.* Nitrosacidococcus tergens” sp. RJ19.

**Fig. 1:**
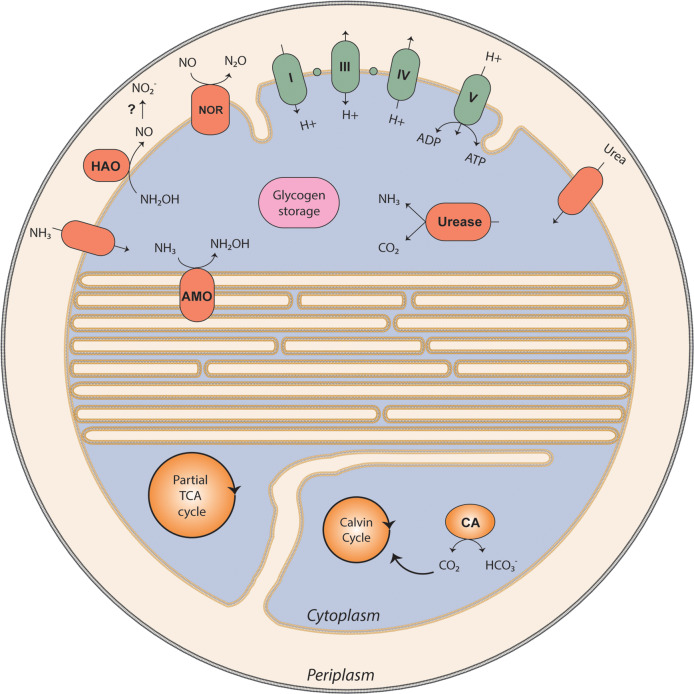
Metabolic cartoon of “*Ca*. Na. tergens” sp. RJ19. AMO ammonia monooxygenase, HAO hydroxylamine oxidoreductase, NOR nitric oxide reductase, CA carbonic anhydrase, TCA tricarboxylic acid. In green are depicted the complexes of the respiratory chain. The small green circles represent cytochromes. A list of all annotated genes can be found in Supplementary Table S7.

Analysis of the genome (see Fig. 1 for a schematic representation) predicted that energy was conserved through the oxidation of NH_3_ to hydroxylamine via AMO (AmoCAB, NSCAC_1277, 1278, 1280). Downstream in the *amoCAB* operon additional genes are found, which show homology to *amoD* and *amoE*. The first oxidation step is followed by the conversion of hydroxylamine to NO by the hydroxylamine-ubiquinone redox module (HURM) [45], consisting of the HAO (HaoAB, NSCAC_1126, 1127) and the quinone-reducing tetraheme cytochrome *c*_M_552 (CycB, NSCAC_1125). Interestingly, the “*Ca*. Na. tergens” sp. RJ19 genome lacks the soluble cytochrome *c*554 (CycA), which is encoded in the same operon (*haoAB-cycAB*) in all other genome-sequenced AOB including comammox *Nitrospira* [7, 46], except the closely related “*Ca*. Ng. terrae” [11]. When comparing the HURM operon structure of “*Ca*. Ng. terrae” and “*Ca*. Na. tergens” it looks like in both cases the CycA encoding gene is lost. While in “*Ca*. Ng. terrae” some remains seem to be present (BAW79846 and BAW79847), we were not able to find remains in the “*Ca*. N. tergens” genome (between NSCAC_1125 and NSCAC_1125). The two genes are separated by only 247 bases. CycA was speculated to shuttle electrons between HAO and cytochrome *c*_M_552 [47], a function that apparently is performed differently in “*Ca*. Na. tergens” sp. RJ19 and “*Ca*. Ng. terrae.” For the further oxidation of NO to NO_2_^−^ the enzyme responsible has not been identified yet, but both, a NO-producing nitrite reductase (NirK) working in reverse and nitrosocyanin have been speculated to catalyze this reaction [4, 48]. However, while nitrosocyanin is encoded by “*Ca*. Na. tergens” sp. RJ19 (NSCAC_0629), its genome does not contain a gene for NirK, as was also reported for “*Ca*. Ng. terrae” [11]. The electrons from ammonia oxidation are transferred to the electron transport chain, which, as in all nitrifiers, works bifurcated, as the entry point of electrons yielded by hydroxylamine oxidation will be the quinone pool. All genes encoding the respiratory complexes I (NSCAC_0235–0248), III (NSCAC_0089–0091), IV (NSCAC_0136, 0137, 1713–1722), and V (NSCAC_1767–1774) were detected in the genome. Conversely, no succinate dehydrogenase (complex II) was found, indicating an incomplete tricarboxylic acid (TCA) cycle that is not directly linked to the respiratory chain.

Although as mentioned above, the “*Ca*. Na. tergens” sp. RJ19 genome did not possess any genes encoding for a NO-forming nitrite reductase, we detected the presence of a gene encoding a NO reductase (NSCAC_1563) for the conversion of NO to N_2_O. This NO reductase is a monomeric type, the only adjacent gene encodes for a formate/nitrite transporter. Furthermore, cytochrome P460 (NSCAC_1129) was present and might cause additional N_2_O production from hydroxylamine detoxification under oxygen-limited conditions [49].

Canonical AmtB-type transporters for ammonia uptake were not detected in the genome. However, a CDS (NSCAC_1037) with 54% amino acid identity to a duplicated putative ammonia permease of *Nitrosococcus oceani* ATCC 19707 (Noc_2700 and Noc_2701) [50] was identified, but the function of this protein in ammonia transport still requires experimental verification. Urea appeared to be an alternative source of ammonia for energy conservation and growth. All genes encoding an urea transporter (NSCAC_0475), the three subunits of the urease enzyme (UreA–UreC—NSCAC_0477–0479) and four different accessory proteins (UreD–UreG—NSCAC_0476, 0480–0482) necessary for the correct assembly of the urease were predicted. Ureolysis could be beneficial for our *Nitrosacidococcus* strain, as, besides ammonium, also CO_2_ is produced as carbon source for assimilation. CO_2_ can be accumulated intracellularly via two types of carbonic anhydrases (NSCAC_0743, 0938) and assimilated by a type I ribulose-1,5-bisphosphate carboxylase/oxygenase (RuBisCO; NSCAC_0587, 0588). The carbonic anhydrases present in the genome of “*Ca*. Na. tergens” do not have signal peptides. This implies a cytoplasmic localization of these enzymes and a possible involvement in proton scavenging and pH homeostasis [14, 51]. In some acidophiles, acid resistance is achieved by a reverse membrane potential and, because of this, a high number of cation transporters is found [52]. In strain RJ19, several proton transporters were identified (Supplementary Table S7), including a Na^+^/H^+^ exchanger (NSCAC_1736). Different types of prokaryotic Na^+^/H^+^ transporters were found to be differentially regulated in relation to pH [53, 54].

All genes for the Calvin–Benson–Bassham (Supplementary Table S7) cycle were identified, except for sedoheptulose 1,7-bis-phosphatase (EC 3.1.3.37). The function of this enzyme is probably catalyzed by fructose-1,6-bisphosphatase (NSCAC_0004) [55], which has been demonstrated to be a bifunctional enzyme hydrolyzing both fructose and sedoheptulose bisphosphate. Phosphoglycolate, which is formed by the oxygenase function of RuBisCO at low CO_2_ concentrations relative to O_2_, is presumably converted to pyruvate by a modified glycolate salvage pathway. First, glycolate is formed by phosphoglycolate phosphatase (EC 3.1.3.18, NSCAC_1268) and further converted to glyoxylate by the secondary reaction of glycerate dehydrogenase (EC 1.1.1.29, SCAC_0419). Finally, glyoxylate is detoxified by a transamination of L-alanine to pyruvate and glycine, catalyzed by alanine–glyoxylate aminotransferase (EC 2.6.1.44, NSCAC_0568). The enzymes necessary for glycolysis and gluconeogenesis were also predicted, as were the oxidative and non-oxidative branches of the pentose phosphate pathway (Supplementary Table S7). Interestingly, only a part of the TCA cycle seemed to be present in “*Ca*. Na. tergens”: genes encoding the 2-oxoglutarate dehydrogenase (EC 1.2.4.2, 2.3.1.61) and succinate dehydrogenase (EC 1.3.5.1) complexes, as well as malate dehydrogenase (EC 1.1.1.37) and pyruvate carboxylase (EC 6.4.1.1), could not be identified. Instead, oxaloacetate is most likely replenished by phosphoenolpyruvate carboxylase (EC 4.1.1.31, NSCAC_1317) and subsequently used for L-aspartate biosynthesis (NSCAC_1014, 1244). From L-aspartate, fumarate can be formed via either adenylo- or L-arginino-succinate intermediates (NSCAC_0583, 0584, 1629, 1756). Together with the fumarate formed here, L-aspartate can also be used to form succinate, as the “*Ca*. Na. tergens” sp. RJ19 genome encodes a L-aspartate oxidase (EC 1.4.3.16, NSCAC_1254), which can use fumarate as an electron acceptor during L-aspartate oxidation [56]. Moreover, fumarate can be converted to malate by a conventional fumarate hydratase (EC. 4.2.1.2, NSCAC_0890). Lastly, malate might be converted back to pyruvate by an oxaloacetate-decarboxylating malate dehydrogenase (EC 1.1.1.40, NSCAC_0061). Carbon storage is achieved by the orchestrated reactions of phosphoglucomutase (EC 5.4.2.2, NSCAC_1039), glucose-1-phosphate adenylyltransferase (EC 2.7.7.27, NSCAC_1118), and glycogen synthase (EC 2.4.1.21, NSCAC_1316), which together form glycogen from D-glucose 6-phosphate.

In addition, the genome of “*Ca*. Na. tergens” sp. RJ19 contained genes encoding the biosynthesis of the 20 amino acids, but threonine and methionine metabolism seemed incomplete. For threonine biosynthesis, no homoserine kinase (EC 2.7.1.39) for the conversion of homoserine to phospho-homoserine was identified, and for methionine the enzymes forming the intermediates cystathionine and homocysteine from either cysteine or aspartate were absent.

### Enrichment of the acidophilic ammonia oxidizer

To facilitate the physiological characterization of “*Ca*. Na. tergens” sp. RJ19, we started to enrich and cultivate this novel nitrifier under aerobic conditions in a continuous flow membrane bioreactor supplied with 76.2 mmoles NH_4_Cl day^−1^ at a constant pH of 3.5. After 150 days, a stable enrichment was obtained and FISH analysis with the newly developed *Nitrosacidococcus*-specific probe Nater1117 (see below), showed that “*Ca*. Na. tergens” sp. RJ19 was the dominant microorganism in the culture. The high level of enrichment was also supported by transcriptomic data (see below). These data also proved that we enriched the dominant bacterium from the air scrubber. At high stirring speed, biomass appeared as a combination of yellow small flocs and planktonic cells; if stirring was decreased or stopped, biofilm formation and big cell aggregates were observed.

**Fig. 2:**
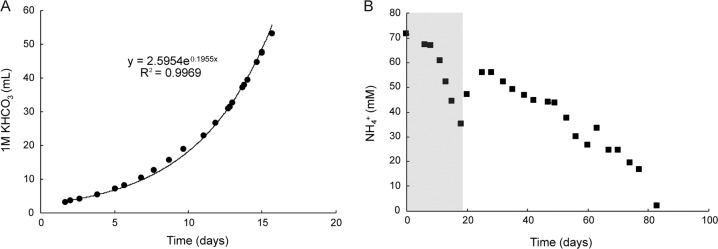
Ammonia-oxidizing activity of the “*Ca*. Na. tergens” sp. RJ19 enrichment. Consumption of **A** KHCO_3_ and **B** NH_4_Cl over time. The gray box in (**B**) indicates the NH_4_^+^ consumption during the exponential growth shown in (**A**). After 20 days the reactor was converted from batch to continuous operation. Error bars are smaller than the symbols and represent the standard deviation over three technical replicates.

The reactor was run as a batch system for 20 days and the pH was kept at 3.5 by supplying sterile 1 M KHCO_3_ solution. Since ammonia oxidation acidifies the medium, the consumption of KHCO_3_ can directly be correlated to growth, and KHCO_3_ consumption indeed increased exponentially over time (Fig. 2A, B). From this, we calculated the growth rate at pH 3.5 to reach 0.196 day^−1^, corresponding to a doubling time of 3.5 days. When an identical batch reactor was inoculated with inactive biomass, no KHCO_3_ consumption or pH drop was observed (Supplementary Fig. S3).

After the enrichment reached a semi-steady state phase, the reactor was switched from batch to continuous mode, with a media exchange rate of 1 L/day, to demonstrate continuous growth. Biomass retention was achieved through membrane filtration and the biomass removal rate was adjusted to 0.2 L/day. The reactor was monitored for protein content and nitrogen compound concentrations, which allowed the calculation of nitrogen conversion rates and mass balances (Table 1). From these data, a total recovery of 90 ± 11% of the ammonium–nitrogen supplied could be achieved, with 7% recovered as NO_2_^−^, 40% as NO_3_^−^, 20% as NO/NO_2_ gas, and 23% still found as not consumed NH_4_^+^. Approximately 1% of the NH_4_^+^-N was assimilated into biomass.

**Table 1:** Nitrogen balance of the “*Ca*. Na. tergens” sp. RJ19 enrichment culture supplied with NH_4_^+^ or urea.

NH_4_^+^ fed (mmol/day)	NO_2_^-^ (mmol/day)	NO_3_^-^ (mmol/day)	NH_4_^+^ not consumed (mmol/day)	NO/NO_2_ (mmol/day)	Protein-N (mmol/day)	Total N (mmol/day)	Recovery (%)
76.2 ± 1.1	5.2 ± 0.5	30.6 ± 2.7	17.7 ± 4.4	14.4 ± 1.0	0.5 ± 0.0	68.4 ± 8.6	90 ± 11

Urea fed (mmol/day)			Urea not consumed (mmol/day)				
31.1 ± 0.0	1.3 ± 0.0	2.4 ± 0.0	27.7 ± 0.5	4.8 ± 0.1	0.4 ± 0.1	36.7 ± 0.6	118 ± 2

The table shows the amount of substrate (NH_4_^+^ or urea) fed to the reactor and the nitrogen products formed (NO_2_^−^, NO_3_^−^, NO/NO_2_). The amount of nitrogen utilized for biomass production is also calculated (protein-N), together with total Nitrogen (Total N). The resulting nitrogen recovery is expressed in %. Values are shown as average ± standard deviation (*n* = 3). NH_4_^+^-feeding experiments were performed in chemostat mode, while urea-fed experiments were performed in the exponential phase of a batch reactor over an 8-h time period.

**Fig. 3:**
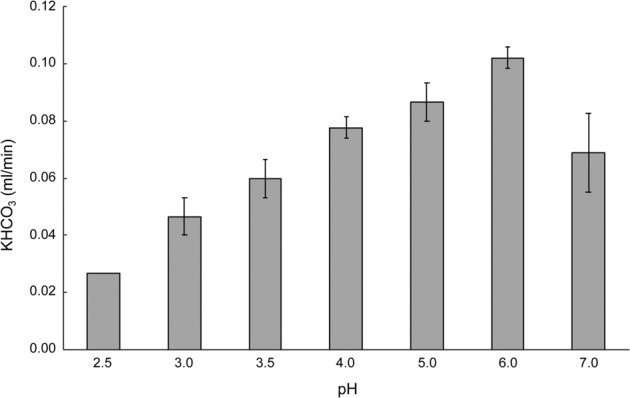
pH range of a “*Ca*. Na. tergens” sp. RJ19 enrichment. The culture shows activity between pH 2.5 and 7, with the highest activity detected at pH 6. Error bars represent the standard deviation over three different experiments.

The activity of the enrichment was also determined at different pH values to estimate the pH optimum of “*Ca*. Na. tergens” sp. RJ19. The culture was active over a pH range from 2.5 to 7, with the optimum at pH 6 (Fig. 3).

### Kinetics of ammonia oxidation

The apparent NH_3_ affinity constant (*K*_*m*(app)_) was calculated by measuring the ammonia-dependent oxygen consumption of “*Ca*. Na. tergens” sp. RJ19. Increasing concentrations of NH_4_Cl were injected into a cell suspension-containing respiration chamber and oxygen consumption was recorded via an oxygen microsensor. Using Michaelis–Menten type curve fitting (Supplementary Fig. S4), a *K*_*m*(app)_ at pH 4.7 of 5.2 ± 0.5 mM NH_4_^+^ + NH_3_ was obtained, which corresponds to 147 ± 14 nM NH_3_ according to the Henderson–Hasselbalch equation. The maximum O_2_ consumption rate (*V*) was calculated at 0.28 ± 0.01 µM O_2_ min^−1^ µg protein^−1^.

### Utilization of urea

**Fig. 4:**
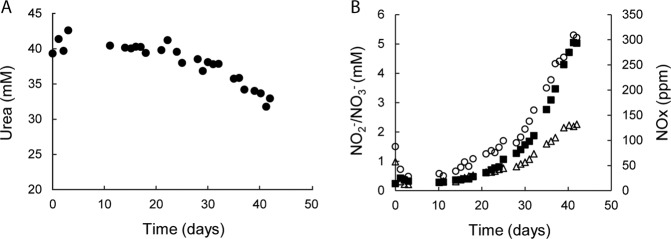
Growth on urea of the “*Ca*. Na. tergens” sp. RJ19 enrichment culture. **A** Urea consumption; black circles represent the average of three technical replicates, error bars indicate the standard deviation of these triplicates. Where not visible, the error bars were smaller than the symbols. **B** Production of NO_2_^−^ (white triangles), NO_3_^−^ (black squares), NOx (NO + NO_2_, white circles).

Since the genome of “*Ca*. Na. tergens” sp. RJ19 revealed the presence of an operon for urea uptake and degradation, we tested whether urea could serve as ammonia and thus energy source at pH 3.5. A batch reactor was set up with medium containing urea as substrate and monitored over a period of 42 days with daily measurements of urea, NO_2_^−^, NO_3_^−^, and NO/NO_2_ concentrations, and protein content (Fig. 4 and Table 1).

After an initial acclimatization phase of 11 days, the culture consumed up to 12 mmol day^−1^ urea, which was converted into NO_2_^−^, NO_3_^−^, and NO (Fig. 4 and Table 1). Unlike ammonia oxidation, urea hydrolysis does not lead to acidification [57], and KHCO_3_ consumption could thus not be used as growth indicator. Therefore, growth rate (*μ*) with urea was calculated from NO_3_^−^ formation, which followed an exponential curve (*y* = 0.711e^0.1038×^, *R*^2^ = 0.997) and indicated a *μ* = 0.104 day^−1^ and a doubling time of 6.7 days. The ratio between urea consumption and product formation was calculated by determining the amount of nitrogen metabolized by the culture over 8 h (Table 1), resulting in a recovery of 118 ± 2% of the urea-nitrogen. The overestimation of the recovered nitrogen could be explained by systematic errors in the measurements, partly due to the difficulty of combining gas and liquid sampling. Based on substrate conversion rate, it was evident that most of the urea fed into the system (89%) was not metabolized and that 4% of the nitrogen was recovered as NO_2_^−^, 8% as NO_3_^−^, and 15% NO/NO_2_ gas, and 1% was assimilated into biomass.

### Morphological characteristics

**Fig. 5:**
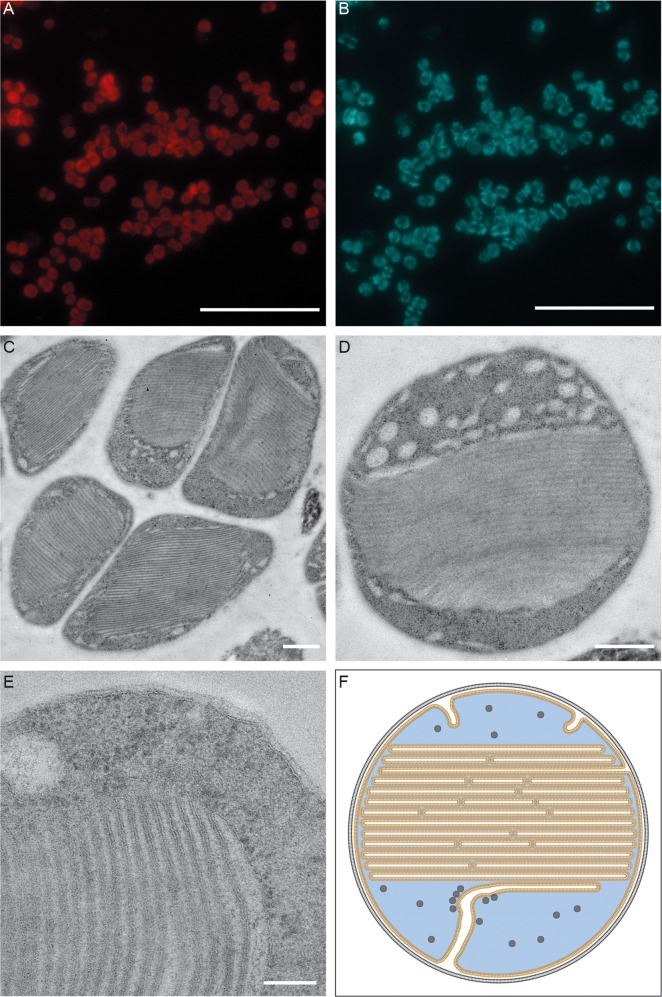
Morphology of “*Ca*. Na. tergens” sp. RJ19. FISH micrographs: **A** cells of “*Ca*. Na. tergens” sp. RJ19 stained with probe Nater1117 (red); **B** cells stained with DAPI (blue). Scale bar = 10 μm. **C**–**E** Electron micrograms. Scale bars = 500 nm. **C** Typical cell cluster of “*Ca*. Na. tergens” sp. RJ19. An EPS layer is visible surrounding the cells. **D** Coccoid cell shape. Membrane stacks are visible in the center of the cell; in white the tubular structures are represented; black dots in the cytoplasm correspond to the ribosomes. **E** “*Ca*. Na. tergens” sp. RJ19 cell in higher magnification. Scale bar = 100 nm. The outer membrane appears less electron dense compared to the inner membrane, and the small periplasm is visible. **F** Schematic representation of a “*Ca*. Na. tergens” sp. RJ19 cell; orange = membrane stacks and inner membrane; white = tubular structures; black circles = ribosomes; blue = cytoplasm; black = outer membrane.

Morphology of “*Ca*. Na. tergens” sp. RJ19 in the enrichment culture was investigated by electron microscopy and FISH. Cells appeared as round cocci with a diameter of 500 nm and often occurred in aggregates of two or more cells (Fig. 5A–C). Transmission electron microscopy revealed a small periplasm and an EPS layer surrounding the cells (Fig. 5C–E). The most evident feature was an extensive system of intracytoplasmic membrane stacks, which supposedly contain the AMO complex [58, 59]. Near the membrane stacks tubule-like structures with low electron density were visible (appearing white; Fig. 5D); these invaginations of the membrane could be membrane stack precursors, where an increasing AMO content would transform the membrane from tubular to a planar conformation [60]. The remaining cytoplasm was densely populated with ribosomes (visible as electron-dense black dots; Fig. 5D, E).

### Transcriptomic analysis

**Fig. 6:**
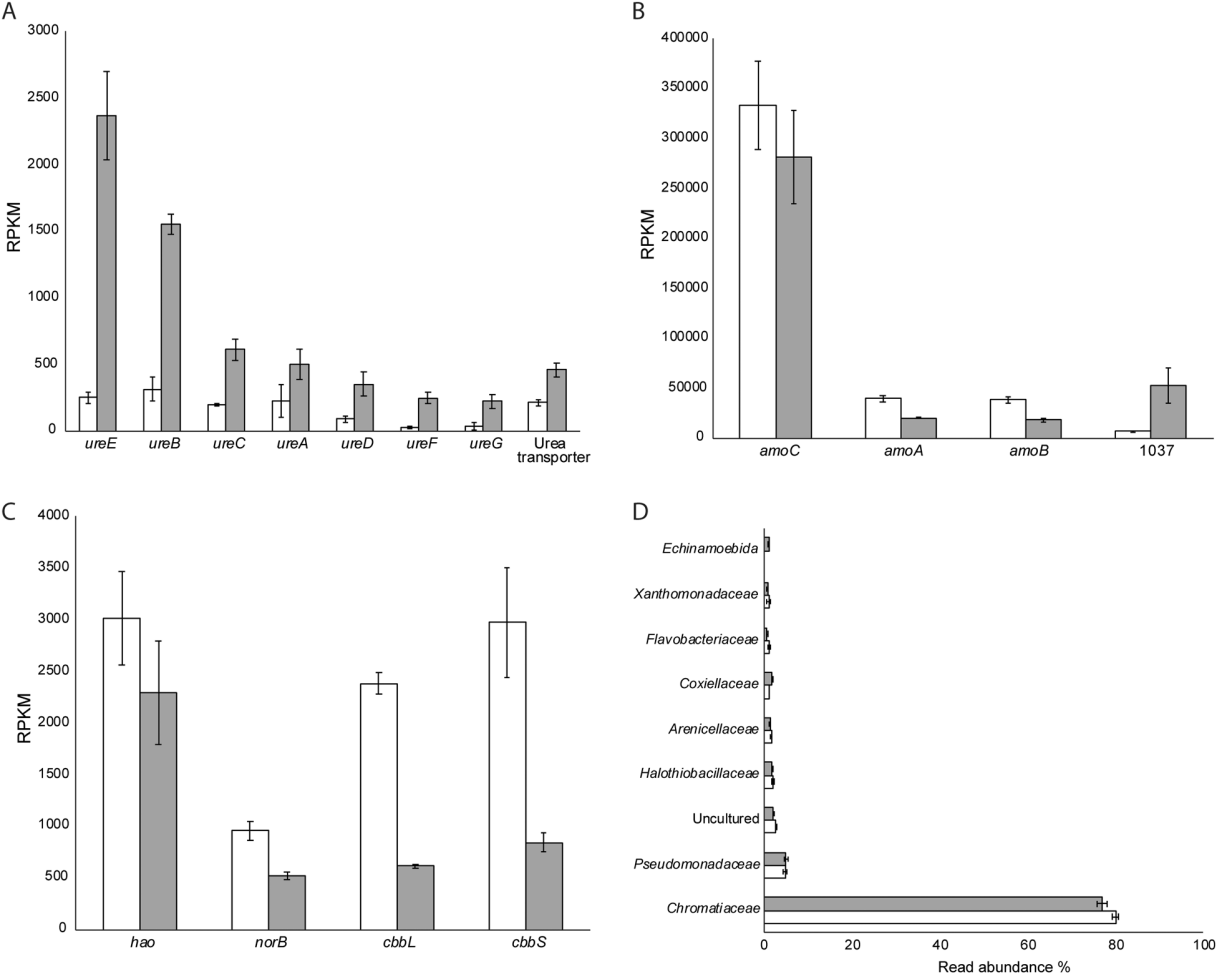
Transcriptomic response of “*Ca*. Na. tergens” sp. RJ19 to growth with ammonia or urea. Genes involved in **A** urea metabolism; **B** ammonia oxidation; **C** hydroxylamine oxidation, NO reduction, and carbon fixation. White bars represent gene expression levels (in reads per kilobase per million mapped reads, RPKM) on ammonia, gray bars on urea. Differences in expression are statistically significant (*p* < 0.05), except for *ureA* (*p* = 0.06, Supplementary Table S8). *1037*: NSCAC_*1037*, putative NH_3_/NH_4_^+^ permease; **D** community composition based on 16S rRNA gene abundance in NH_4_^+^ (white bars) and urea culture (grey bars). In both reactors, *Chromatiaceae*, the family that includes “*Ca*. N. tergens” RJ19, is the most abundant.

The gene expression patterns of “*Ca*. Na. tergens” sp. RJ19 utilizing ammonia or urea as a substrate was compared using transcriptomics (Fig. 6). As expected, genes involved in urea metabolism were clearly upregulated during growth on urea (Fig. 6A). Contrastingly, the subunits of AMO (*amoCAB*) showed higher expression levels in the culture fed with ammonium as substrate (*p* < 0.05; Fig. 6B and Supplementary Table S8); however, *amoCAB* was still among the most highly expressed genes in the urea culture. Under both conditions, *amoC* had the highest RPKM value among the three subunits. The putative NH_3_/NH_4_^+^ permease (NSCAC_1037) was threefold higher expressed during growth on urea (*p* = 0.00; Fig. 6B), whereas hydroxylamine oxidoreductase (*hao*), NO reductase (*norB*) and, interestingly, also RuBisCO (*cbbLS*) had lower expression levels compared to the ammonium-fed culture (Fig. 6C).

Since total RNA was used for transcriptome analysis, the 16S rRNA sequences were extracted and mapped against the SILVA database, to investigate the community composition. *Chromatiaceae*, the family that included “*Ca*. N. tergens” RJ19, resulted to be the most abundant (~80%) in both cultures (Fig. 6D). Furthermore, when the 16S rRNA reads were mapped against the genome of “*Ca*. Na. tergens” sp. RJ19 (cut-off 98% identity), 96% of the reads for the ammonium and 92% for the urea reactor were recruited by the genome of this microorganism. These result, combined with the FISH pictures (Fig. 5A, B), showed that this novel ammonia-oxidizing bacterium had reached a high level of enrichment in both reactors.

## Discussion

In this study, we characterized a species of a novel gammaproteobacterial ammonia-oxidizing bacterium, for which we proposed the name of “*Ca.* Nitrosacidococcus tergens” sp. RJ19. This organism showed the ability to oxidize ammonia and grow at pH values as low as 2.5, representing the most acidophilic ammonia oxidizer reported to date. The previously described acidophilic ammonia oxidizers “*Ca*. Nitrosotalea devanaterra” [12] and “*Ca*. Ng. terrae” [11] exhibited activity at pH of 4 and 5, respectively. In addition, *Nitrosococcus*-like species were found to be dominant in reactors treating human urine down to pH 2.2 [37]. The ability of oxidizing ammonia at low pH could be due to different adaptation mechanisms, metabolic flexibility, or by the cooperation between different species in the environment [5, 13, 20]. However, the challenge of very low NH_3_ availability at acidic pH could also be solved with high ammonia affinities. In this regard, “*Ca*. Na. tergens” sp. RJ19 showed a *K*_*m*(app)_ for NH_3_ of 147 ± 14 nM. Lower values were only reported for specialized marine AOA and for the comammox species *Nitrospira inopinata*, which thrive at extremely low substrate concentrations [61–63]. Despite the low *K*_*S*(app)_ of *Nitrosacidococcus*, we could not yet isolate this strain in pure culture using dilution to extinction or floating filter techniques [64, 65]. We could obtain colonies on floating filters but no growth was observed after transferring single colonies to liquid medium.

*“Ca*. Na. tergens” sp. RJ19, like many other ammonia oxidizers [11, 22], encoded genes for urea hydrolysis and could use it as source of ammonia for growth. Growth on urea, however, was less efficient compared to NH_3_ and our calculations indeed showed that 89% of the urea fed into the system was not consumed. Moreover, when lower concentrations of urea were supplied, activity was lost from the reactor (data not shown). This could suggest that this organism had a low affinity for urea, or that transport efficiency into the cytoplasm was ineffective for some reason. Alternatively, the export of ammonia formed during urea degradation into the periplasm, or the pseudo-periplasmic space within the intracytoplasmic membrane stacks, might be inefficient due to the lack of canonical ammonia transporters. As the active site of the AMO complex is assumed to be located in the periplasm [58, 66], this would severely limit substrate availability and might additionally cause substrate inhibition and possibly even toxicity in the cytoplasm. This latter hypothesis would also explain why the putative NH_3_/NH_4_^+^ permease (NSCAC_1037) was upregulated during growth on urea (Fig. 6B). The growth rate of “*Ca*. Na. tergens” sp. RJ19 on ammonium was almost twice as fast as in the presence of urea (0.195 vs. 0.104 days^−1^ at pH 3.5). This was still significantly slower compared to the rates of the other acidophilic ammonia oxidizers, “*Ca*. N. devanaterra” (0.23–0.37 days^−1^ at pH 4.5) [12] and “*Ca*. Ng. terrae” sp. TAO100 (0.89 day^−1^ at pH 6) [11]. However, we did not determine the growth rate of “*Ca*. Na. tergens” sp. RJ19 at optimal pH.

The products of ammonia and urea oxidation included NO and NO_2_^−^ as well as chemically formed NO_3_^−^. NO is a toxic compound that can be reduced to N_2_O via NO reductase (EC 1.7.2.5) [67], which was detected in the genome of “*Ca*. Na. tergens” sp. RJ19 (*norB*, NSCAC_1563) and expressed at levels of 955 and 525 RPKM in the ammonium and urea cultures, respectively. Nevertheless, this bacterium did not seem to be inhibited by high concentrations of NO, and the presence of N_2_O was not detected in the off-gas of the culture. Further studies will be necessary to understand if the expression of *norB* is actually linked to enzyme activity.

Several metabolic pathways identified in the genome of “*Ca*. Na. tergens” sp. RJ19 appeared to be incomplete. In the Calvin–Benson–Bassham cycle, the gene for sedoheptulose 1,7-bis-phosphatase (EC 3.1.3.37) could not be identified; however, fructose-1,6-bis-phosphatase (EC 3.1.3.11, NSCAC_0004) might be bifunctional and also function in sedoheptulose 1,7-bis-phosphate hydrolysis, as shown for the enzymes in *Ralstonia metallidurans* and *Xanthobacter flavus* [68, 69]. Additionally, the interconversion of fructose-6-phosphate and fructose-1,6-bis-phosphate during glycolysis and glyconeogenesis can be carried out by a reversible pyrophosphate-dependent 6-phosphofructose-1-kinase (EC 2.7.1.90, NSCAC_1402) [69, 70]. In addition, the TCA cycle also was predicted to be incomplete (see above).

Interestingly, the genome of “*Ca*. Na. tergens” sp. RJ19 did not contain any amtB-type ammonia transporters; this was also the case for the acid-tolerant “*Ca*. Ng. terrae” sp. TAO100, for which it was proposed that NH_3_ transport relied on passive diffusion over the membrane [11, 14]. In “*Ca*. Na. tergens” sp. RJ19, the gene NSCAC_1037 showed 54% identity to a putative NH_3_/NH_4_^+^ permease of *Nitrosococcus oceani* ATCC 19707 (Noc_2700 and Noc_2701) [50]. This gene was threefold upregulated (*p* = 0.00) when the culture grew on urea, which might indicate that NSCAC_1037 is involved in NH_3_/NH_4_^+^ transport, although its role would be in ammonia export during urea degradation.

Contrary to *Nitrosococcus* species [71], “*Ca*. Na. tergens” sp. RJ19 does not seem to be distributed in marine environments. Metagenomic sequence data (IMNGS platform) showed that closely related sequences (based on 16S rRNA gene similarity) were found in a refuse dump of leaf-cutter ants [36], in a soil in Finland and in a synthetic urine nitrification reactor [37], which all are environments with significant nitrogen cycling and high ammonium concentrations. Furthermore, similar 16S rRNA gene sequences were retrieved from sewage and bat guano (Supplementary Fig. S2A). High ammonium or urea concentrations seem to be a common factor selecting for this novel nitrifying bacterium in an ecosystem. More metagenomic data and implementation of the primers we used to detect this species (Supplementary Table S1) could yield more information regarding the distribution and abundance of this organism in the environment.

In conclusion, we were able to obtain a highly enriched culture of a novel ammonia-oxidizing bacterium that showed activity and growth even at pH 2.5. This autotrophic microorganism had a high ammonia affinity and was able to use urea as alternative ammonia and thus energy source. The growth rate at pH 3.5 was calculated at 0.2 day^−1^ with ammonium and 0.1 day^−1^ with urea, and its morphology was similar to the closest relatives within the genera *Ca*. Ng. and *Nitrosococcus* [11, 50]. Phylogenetic and genomic analysis showed that this bacterium belonged to a novel species and genus, for which we proposed the name of “*Ca.* Nitrosacidococcus tergens” sp. RJ19. Taken together, our findings expand the knowledge about acidophilic microorganisms and confirm prior observation of oxidation of ammonia at pH as low as 2.5.

### Description of *Ca*. Nitrosacidococcus tergens gen. nov. sp. nov

Ni.tro.sa.ci.do.coc’cus. L. masch. adj. *nitrosus*, full of natron, here intended to mean nitrous; L. neut. n. *acidum*, an acid; from L. masc. adj. *acidus*, sour; M.L. masc. n. *coccus*, sphere; from Gr. masc. n. *kokkos*, grain, seed; N.L. masc. n. *Nitrosacidococcus*, an acid-loving nitrous sphere; ter’gens. L. v. tergere, to clean; L. part. adj. *tergens*, cleaning, named thus for its ability to clean the air of ammonia in an air scrubber. Cells are Gram-negative, round cocci with a diameter of 0.5 µm and contain an extensive system of intracytoplasmic membrane stacks. Obligate chemolithotroph that oxidizes ammonia to nitrite. Urea can be utilized as alternative energy source. At pH 3.5, growth rate is 0.1 day^−1^ on ammonia and 0.2 day^−1^ on urea. The optimum pH for growth is 6, but growth is observed between pH 2.5 and 7. Cells exhibit a *K*_*m*(app)_ for NH_3_ of 147 ± 14 nM. The type strain RJ19^T^ was enriched from an air-cleaning bioscrubber of a pig farm in Erp, The Netherlands. The DNA G + C content of the type strain calculated on the basis of the genome sequence is 37 % and the genome size is 1.8 Mbp.

## Supplementary information

Supplementary Information

## Data Availability

The complete genome sequence of “*Ca*. Na. tergens” sp. RJ19 has been deposited in NCBI BioProject database with accession number PRJEB36691.
