# Supplementary material for: Ammonia oxidation at pH 2.5 by a new gammaproteobacterial ammonia-oxidizing bacterium
Source: ISME J. 2020 Dec 10;15(4):1150–64. doi: 10.1038/s41396-020-00840-7 (PMC8115276; doi:10.1038/s41396-020-00840-7)
Supplement: Supplementary file 1 — Supplementary Information [file 41396_2020_840_MOESM1_ESM.pdf]

Supplementary Information

Ammonia oxidation at pH 2.5 by a new gammaproteobacterial ammonia-oxidizing bacterium

Nunzia Picone[1], Arjan Pol[1], Rob Mesman[1], Maartje A.H.J. van Kessel[1], Geert Cremers[1], Antonie H. van Gelder[2], Theo van Alen[1], Mike S.M. Jetten[1], Sebastian Lücker[1] and Huub J.M. Op den Camp[1*]

[1]Department of Microbiology, IWWR, Radboud University, Heyendaalseweg 135, NL-6525 AJ, Nijmegen, The Netherlands

[2]Department of Agrotechnology and Food Sciences, Wageningen University, Stippeneng 4, NL-6708 WE, Wageningen, The Netherlands

*Correspondent author

Huub J.M. Op den Camp

h.opdencamp@science.ru.nl

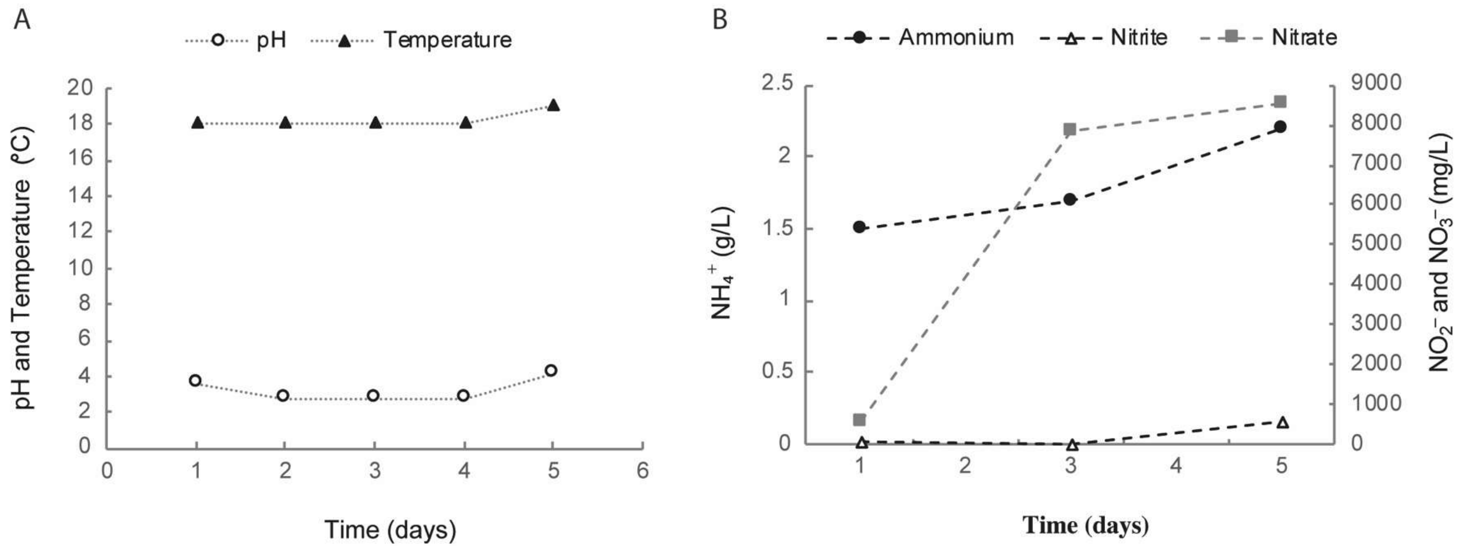

Figure S1. Characteristics of the acidic air scrubber. A) pH and Temperature registered in the air scrubber. B) Concentrations of NH_4^+, NO_2^- and NO_3^- in the washing water.

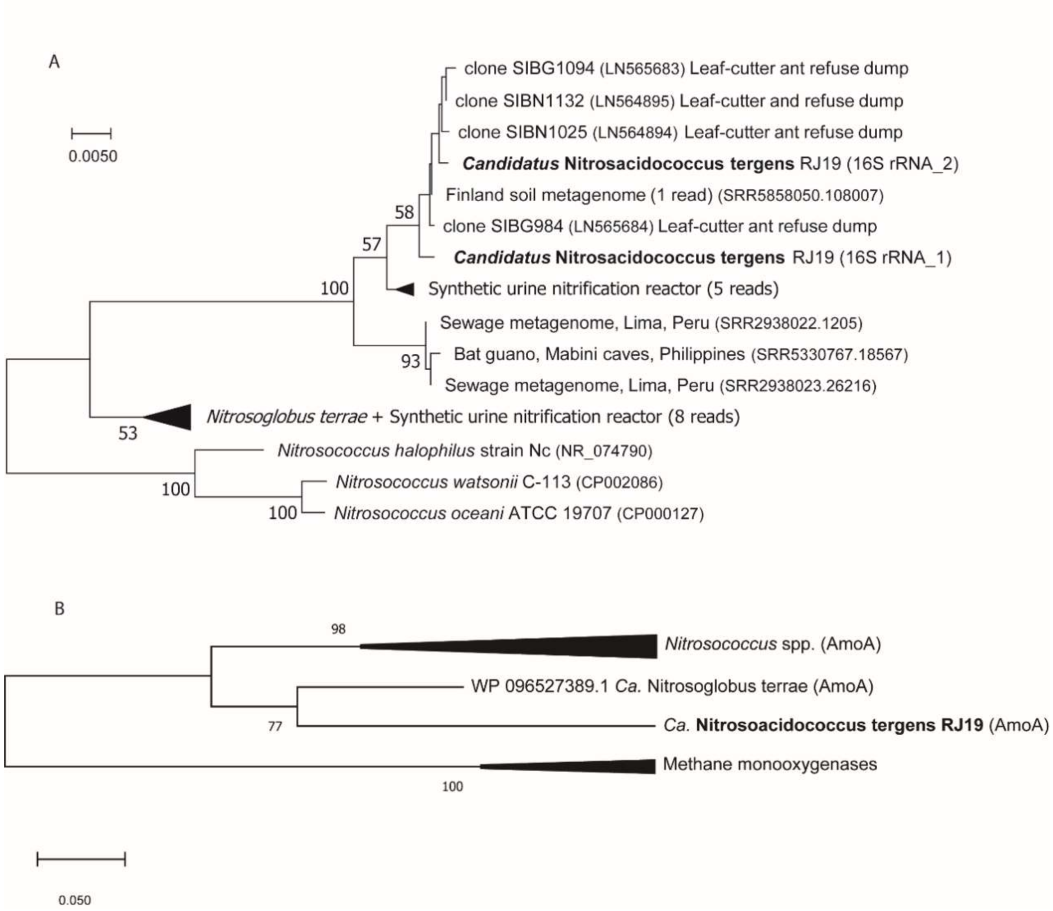

Figure S2. (A) 16S rRNA gene-based phylogenetic tree of "*Ca*. Nitrosoacidococcus tergens" RJ19, other nitrifiers and environmental sequences. The evolutionary history was inferred using the Neighbor-Joining method. The optimal tree with the sum of branch length = 0.22931256 is shown. The percentage of replicate trees in which the associated taxa clustered together in the bootstrap test (500 replicates) are shown next to the branches if >50 . The tree is drawn to scale, with branch lengths in the same units as those of the evolutionary distances used to infer the phylogenetic tree. The evolutionary distances were computed using the Jukes-Cantor method and are in the units of the number of base substitutions per site. The analysis involved 29 nucleotide sequences. All ambiguous positions were removed for each sequence pair. There were a total of 1541 positions in the final dataset. **(B)** AmoA phylogenetic tree. The evolutionary history was inferred by using the Maximum Likelihood method and JTT matrix-based model. The tree with the highest log likelihood (-2179.61) is shown. The percentage of trees in which the associated taxa clustered together is shown next to the branches. Initial tree(s) for the heuristic search were obtained automatically by applying Neighbor-Join and BioNJ algorithms to a matrix of pairwise distances estimated using a JTT model, and then selecting the topology with superior log likelihood value. The tree is drawn to scale, with branch lengths measured in the number of substitutions per site. This analysis involved 13 amino acid sequences. There were a total of 255 positions in the final dataset. Evolutionary analyses were conducted in MEGA X. The group "*Nitrosococcus* spp." contains 7 AmoA sequences of different members of the *Nitrosococcus* genus. The group "Methane monooxygenases" includes 4 methane/ammonia monooxygenases subunit A sequences from *Methylosarcina lacus* (WP02298805.1), an uncultured bacterium from Pavin lake (ARX77274.1), *Methylomonas* sp. (PPD34918.1), *Methylobacter* sp. (PPC90499.1).

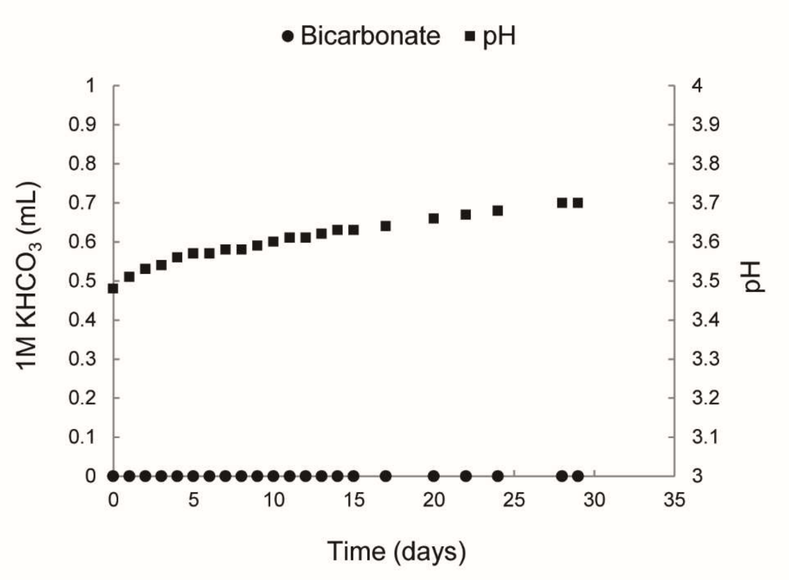

Figure S3. $KHCO_3$ consumption and pH trend in a bioreactor inoculated with inactive "*Ca.* N. tergens RJ19" biomass. $KHCO_3$ was not consumed and pH slightly increased.

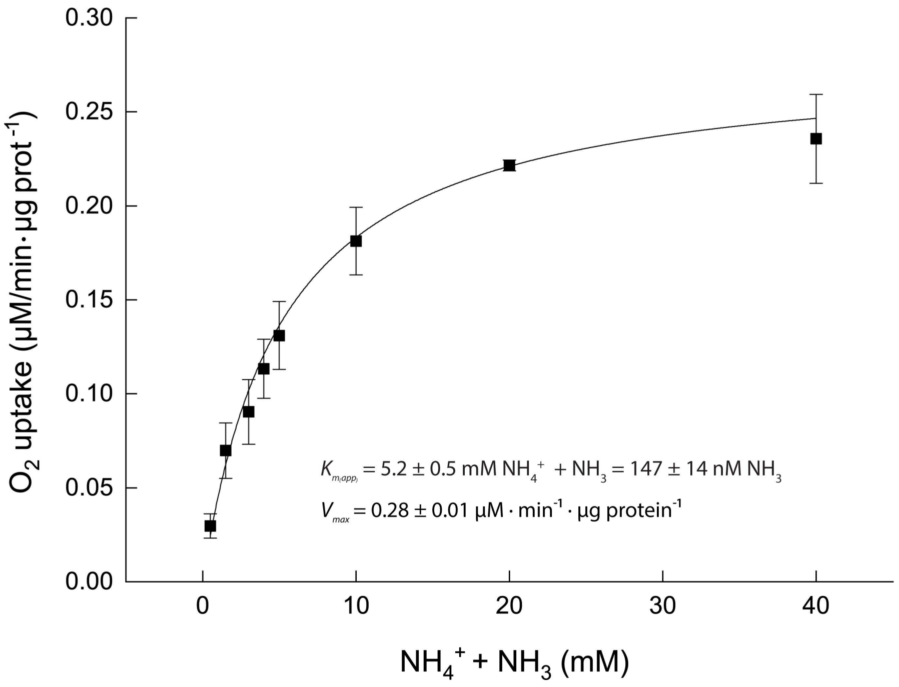

Figure S4. Ammonia oxidation kinetics of the "*Ca.* Na. tergens" sp. RJ19 enrichment culture. Black squares represent the average of three biological replicates. Bars indicate the standard deviation over the three measurements. The black curve represents the best nonlinear least-squares fit using Michaelis-Menten-type kinetics (R^2 = 0.998).

Table S1. PCR primers used in this study.

Target	Primer name	Sequence	T_m (°C)	Product size (bp)	Reference
Genome gap #1	1_Nac_370455f	5'- GAC GCT TAC CAT AGC GCT CA -3'	58.5	645	This work
Genome gap #1	1_Nac_371080r	5'- CAG TGG TAG TGC TAG AGC AG -3'	56.4		This work
Genome gap #2	2_Nac_1370669f	5'- TGC TGT CGT CCA TAC ATG GC -3'	56.6	619	This work
Genome gap #2	2_Nac_1371268r	5'- GCA TTA CAA GAG CAG AAG TGC -3'	55.9		This work
Genome gap #3	3_Nac_1402503f	5'- GAG CTA GCG TAA TAG TGA GTG -3'	55.2	697	This work
Genome gap #3	3_Nac_1403179r	5'- CTG TCC AAG TAC TGG TAG CTG A -3'	56.8		This work
pGEM-T Easy plasmid	M13F	5'-GGT TTT CCC AGT CAC GAC-3'	56		BaseClear B.V.
pGEM-T Easy plasmid	M13R	5'-AGC GGA TAA CAA TTT CAC AC-3'	52		BaseClear B.V.
16S rRNA	16Snitro2F	5'-GGA ATT ACT GGG CGT AAA GG-3'	53	~630	This work
16S rRNA	16Snitro2R	5'-CCT TCC TCC GTT TTG TCA A-3'	51.8		This work
Ammonia monooxygenase subunit A	amoAnitro1F	5'-TKG AYT GGA AAG ATC GCC G-3'	55	421	This work
Ammonia monooxygenase subunit A	amoAnitro1R	5'-CCA GTR CGM ACA TAM TG-3'	48		This work

Table S2. Efficiency measurements of NH_3 and odour removal in the acidic air scrubber.

Component	Location	Unit	Value
NH_3	Before air scrubber	mg/m^3_0	16
	After air scrubber	mg/m^3_0	0.13
	Performance	%	99
	Air	mg/m^3_0	0.039
Odour	Before air scrubber	OU_E/m^3	5500
	After air scrubber	OU_E/m^3	1160
	Performance	%	79
	Air	OU_E/m^3	78

Table S3. Composition of the bacterial community in the acidic air scrubber. The metagenome reads were mapped against the Silva database for 16S rRNA analysis. Values are expressed as % of the total read count and reported on Genus level. The group "others" contains genera with total read count ≤ 2%.

**Genus**	**Abundance (%)**
uncultured	20.8
Castellaniella	8.9
Nitrosococcus-like	6.4
Moheibacter	3.6
Comamonas	2.9
Salmonella	2.9
Pseudomonas	2.7
Vibrio	2.6
Pseudoxanthomonas	2.3
Ottowia	2.2
Rhodanobacter	2.0
others*	42.6

Table S4. 16S rRNAsequence identity between "*Ca.* N. tergens RJ19", *Nitrosococcus oceani* ATCC 19707, *Nitrosococcus halophilus* Nc4, *Nitrosococcus watsonii* C-113 and "*Ca.* Nitrosoglobus terrae TAO100".

	**"*Ca.* N. tergens RJ19"**	***N. oceani* ATCC 19707**	***N. halophilus* Nc4**	***N. watsonii* C-113**
***N. oceani* ATCC 19707**	91%			
***N. halophilus* Nc4**	91%	96%		
***N. watsonii* C-113**	91%	99%	96%	
**"*Ca.* N. terrae TAO100"**	92%	95%	95%	95%

Table S5. Average Nucleotide Identity (ANI) of "*Ca.* N. tergens RJ19" and *Nitrosococcus oceani* ATCC 19707, *Nitrosococcus halophilus* Nc4, *Nitrosococcus watsonii* C-113 and "*Ca.* Nitrosoglobus terrae TAO100".

	***"Ca.* N. tergens RJ19"**	***N. oceani* ATCC 19707**	***N. halophilus N*c4**	***N. watsonii* C-113**
***N. oceani* ATCC 19707**	81%			
***N. halophilus* Nc4**	78.5%	78.6%		
***N. watsonii* C-113**	78.6%	88.8%	74.5%	
***"Ca.* N. terrae" TAO100**	76.2%	74.8%	74.5%	74.8%

Table S6. Ammonia monooxygenase (AmoA) amino acid sequence identity between" *Ca.* N. tergens RJ19" and *Nitrosococcus oceani* ATCC 19707, *Nitrosococcus halophilus* Nc4, *Nitrosococcus watsonii* C-113 and "*Ca.* Nitrosoglobus terrae TAO100".

	***"Ca.* N. tergens RJ19"**	***N. oceani* ATCC 19707**	***N. halophilus* Nc4**	***N. watsonii* C-113**
***N. oceani* ATCC 19707**	71%			
***N. halophilus* Nc4**	72%	85%		
***N. watsonii* C-113**	72%	97%	84%	
***"Ca.* N. terrae" TAO100**	78%	74%	79%	75%

Table S7. "*Ca.* Nitrosacidococcus tergens RJ19" proteins with predicted functions.[a] "*Ca.* N. tergens" protein identifier; [b] Organism with highest scoring BLAST hit to "*Ca*. N. tergens RJ19" protein in SwissProt database. In parentheses: SwissProt accession number of best hit, amino acid identity.[c] Organism with highest scoring BLAST hit to "*Ca*. N. tergens RJ19" protein in TrEMBL database. In parentheses: TrEMBL accession number of best hit, amino acid identity.

**Energy metabolism**					
	**COMPLEX I**				
**Gene**	**Product**	**EC no.**	**CDS[a]**	**Best BLAST hit in SwissProt[b]**	**Best BLAST hit in TrEMBL[c]**
nuoA	NADH ubiquinone oxidoreductase subunit A	1.6.99.5	NSCAC _0235	*Acidithiobacillus ferrooxidans* ATCC 53993 (B5EN71, 73.73%)	*Nitrosococcus halophilus* Nc4 (D5C2P5, 83.9%)
nuoB	NADH ubiquinone oxidoreductase subunit B	1.6.99.5	NSCAC _0236	*Nitrosococcus oceani* ATCC 19707 / NCIMB 11848 (Q3J829, 94.3%)	*Nitrosococcus oceani* AFC27 (B6C6D6, 94.3%)
nuoC	NADH ubiquinone oxidoreductase subunit C	1.6.99.5	NSCAC _0237	*Nitrosococcus oceani* ATCC 19707 / NCIMB 11848 (Q3J830, 69.91%)	*Nitrosococcus oceani* AFC27 (B6C6D5, 69.91%)
nuoD	NADH ubiquinone oxidoreductase subunit D	1.6.99.5	NSCAC _0238	*Nitrosococcus oceani* ATCC 19707 / NCIMB 11848 (Q3J831, 88.49%)	*Nitrosococcus halophilus* Nc4 (D5C2P2, 88.01%)
nuoE	NADH ubiquinone oxidoreductase subunit E	1.6.99.5	NSCAC _0239	*Rickettsia bellii* RML369-C (Q1RJJ1, 47.22%)	*Nitrosococcus halophilus* Nc4 (D5C2P1, 77.64%)
nuoF	NADH ubiquinone oxidoreductase subunit F	1.6.99.5	NSCAC _0240	*Rickettsia bellii* OSU 85-389 (A8GYE0, 54.79%)	*Nitrosococcus halophilus* Nc4 (D5C2P0, 79.95%)
nuoG	NADH ubiquinone oxidoreductase subunit G	1.6.99.5	NSCAC _0241	*Macaca fascicularis* (Q4R6K9, 37.17%)	*Nitrosococcus halophilus* Nc4 (D5C2N9, 64.65%)
nuoH	NADH ubiquinone oxidoreductase subunit H	1.6.99.5	NSCAC _0242	*Nitrosococcus oceani* ATCC 19707 / NCIMB 11848 (Q3J835, 82.23%)	*Nitrosococcus oceani* AFC27 (B6C6D0, 82.23%)
nuoI	NADH ubiquinone oxidoreductase subunit I	1.6.99.5	NSCAC _0243	*Nitrosococcus oceani* ATCC 19707 / NCIMB 11848 (Q3J836, 88.89%)	*Nitrosococcus oceani* AFC27 (B6C6C9, 88.89%)
nuoJ	NADH ubiquinone oxidoreductase subunit J	1.6.99.5	NSCAC _0244	*Cyanidium caldarium* (P48925, 42.27%)	*Nitrosococcus halophilus* Nc4 (D5C2N6, 73.87%)
nuoK	NADH ubiquinone oxidoreductase subunit K	1.6.99.5	NSCAC _0245	*Methylotenera mobilis* JLW8 / ATCC BAA-1282 / DSM 17540 (C6WX51, 75.25%)	*Nitrosococcus halophilus* Nc4 (D5C2N5, 87.13%)
nuoL	NADH ubiquinone oxidoreductase subunit L	1.6.99.5	NSCAC _0246	*Neisseria meningitidis* serogroup A / serotype 4A strain Z2491 (Q9JX92, 55.77%)	*Nitrosococcus oceani* ATCC 19707 / NCIMB 11848 (Q3J839, 77.73%)
nuoM	NADH ubiquinone oxidoreductase subunit M	1.6.99.5	NSCAC _0247	*Rhodobacter capsulatus* (P50974, 45.96%)	*Nitrosococcus halophilus* Nc4 (D5C2N3, 78.4%)
nuoN	NADH ubiquinone oxidoreductase subunit N	1.6.99.5	NSCAC _0248	*Nitrosococcus oceani* ATCC 19707 / NCIMB 11848 (Q3J841, 73.64%)	*Nitrosococcus watsoni* C-113 (D8KAZ8, 74.27%)
	**COMPLEX III**				
petC	Quinol-cytochrome c reductase, cytochrome c1 subunit	1.10.2.2	NSCAC _0089	*Allochromatium vinosum* ATCC 17899 / DSM 180 / NBRC 103801 / D (O31216, 48.57%)	*Nitrosococcus halophilus* Nc4 (D5C3G7, 65%)
petB	Quinol-cytochrome c reductase, cytochrome b subunit	1.10.2.2	NSCAC _0090	*Allochromatium vinosum* ATCC 17899 / DSM 180 / NBRC 103801 / D (O31215, 61.88%)	*Nitrosococcus watsoni* C-113 (D8K9S4, 70.56%)

petA	Quinol-cytochrome c reductase, iron-sulfur subunit	1.10.2.2	NSCAC_0091	*Allochromatium vinosum* ATCC 17899 / DSM 180 / NBRC 103801 / D (O31214, 61.66%)	*Nitrosococcus watsoni* C-113 (D8K9S3, 75.49%)
	**COMPLEX IV**				
coxB	Cytochrome c oxidase, subunit II	1.9.3.1	NSCAC_0136	*Thermus thermophilus* (P98052, 30.28%)	*Nitrosococcus oceani* ATCC 19707 / NCIMB 11848 (Q3J6Y3, 65.52%)
coxA	Cytochrome c oxidase, subunit I	1.9.3.1	NSCAC_0137	*Allomyces macrogynus* (P80440, 22.32%)	*Nitrosococcus oceani* ATCC 19707 / NCIMB 11848 (Q3J6Y4, 70.12%)
	Cytochrome oxidase biogenesis protein SCO1/SenC		NSCAC_0138	*Pseudomonas stutzeri* (P47206, 29.26%)	*Nitrosococcus oceani* ATCC 19707 / NCIMB 11848 (Q3J6Y5, 58.38%)
	conserved membrane protein of unknown function, cytochrome oxidase associated		NSCAC_0139		*Nitrosococcus halophilus* Nc4 (D5BVI2, 67.96%)
ctaB	Protoheme IX farnesyltransferase	2.5.1.-	NSCAC_1713	*Nitrosococcus oceani* ATCC 19707 / NCIMB 11848 (Q3J6R6, 80.74%)	*Nitrosococcus halophilus* Nc4 (D5C4A2, 81.42%)
ctaA	Heme A synthase	1.3.-.-	NSCAC_1714	*Sphingomonas wittichii* RW1 / DSM 6014 / JCM 10273 (A5VF68, 24.93%)	*Nitrosococcus watsoni* C-113 (D8K4K7, 66.18%)
	putative Cytochrome oxidase biogenesis protein Surf1		NSCAC_1716	*Schizosaccharomyces pombe* 972 / ATCC 24843 (Q9Y810, 26.91%)	*Nitrosococcus watsoni* C-113 (D8K4K9, 46.53%)
ctaE	Cytochrome c oxidase, subunit III	1.9.3.1	NSCAC_1718	*Latimeria chalumnae* (O03170, 40.56%)	*Nitrosococcus halophilus* Nc4 (D5C497, 70.14%)
ctaG	Cytochrome c oxidase assembly protein CtaG		NSCAC_1719	*Arabidopsis thaliana* (Q8GWR0, 35.45%)	*Nitrosococcus oceani* ATCC 19707 / NCIMB 11848 (Q3J6R0, 60.22%)
ctaD	Cytochrome c oxidase, subunit I	1.9.3.1	NSCAC_1721	*Bradyrhizobium diazoefficiens* JCM 10833 / IAM 13628 / NBRC 14792 / USDA 110 (P31833, 53.32%)	*Nitrosococcus halophilus* Nc4 (D5C495, 78.05%)
ctaC	Cytochrome c oxidase subunit II	1.9.3.1	NSCAC_1722	*Rickettsia bellii* RML369-C (Q1RI44, 38.98%)	*Nitrosococcus halophilus* Nc4 (D5C494, 74.9%)
	**COMPLEX V**				
atpC	F1 sector of membrane-bound ATP synthase, epsilon subunit	3.6.3.14	NSCAC_1767	*Nitrosococcus oceani* ATCC 19707 / NCIMB 11848 (Q3J6N2, 72.46%)	*Nitrosococcus halophilus* Nc4 (D5C418, 75%)
atpD	F1 sector of membrane-bound ATP synthase, beta subunit	3.6.3.14	NSCAC_1768	*Nitrosococcus oceani* ATCC 19707 / NCIMB 11848 (Q3J6N1, 94.76%)	*Nitrosococcus watsoni* C-113 (D8K4P4, 95.2%)
atpG	F1 sector of membrane-bound ATP synthase, gamma subunit	3.6.3.14	NSCAC_1769	*Nitrosococcus oceani* ATCC 19707 / NCIMB 11848 (Q3J6N0, 78.28%)	*Nitrosococcus halophilus* Nc4 (D5C420, 82.29%)
atpA	F1 sector of membrane-bound ATP synthase, alpha subunit	3.6.3.14	NSCAC_1770	*Nitrosococcus oceani* ATCC 19707 / NCIMB 11848 (Q3J6M9, 87.77%)	*Nitrosococcus watsoni* C-113 (D8K4P6, 88.16%)
atpH	F1 sector of membrane-bound ATP synthase, delta subunit	3.6.3.14	NSCAC_1771	*Nitrosococcus oceani* ATCC 19707 / NCIMB 11848 (Q3J6M8, 60.22%)	*Nitrosococcus watsoni* C-113 (D8K4P7, 59.12%)
atpF	F0 sector of membrane-bound ATP synthase, subunit b	3.6.3.14	NSCAC_1772	*Nitrosococcus oceani* ATCC 19707 / NCIMB 11848 (Q3J6M7, 74.36%)	*Nitrosococcus watsoni* C-113 (D8K4P8, 74.36%)
atpE	F0 sector of membrane-bound ATP synthase, subunit c	3.6.3.14	NSCAC_1773	*Nitrosococcus oceani* ATCC 19707 / NCIMB 11848 (Q3J6M6, 86.02%)	*Nitrosococcus halophilus* Nc4 (D5C424, 89.36%)
atpB	F0 sector of membrane-bound ATP synthase, subunit a	3.6.3.14	NSCAC_1774	*Methylococcus capsulatus* ATCC 33009 / NCIMB 11132 / Bath (Q60CS0, 52.78%)	*Nitrosococcus halophilus* Nc4 (D5C425, 76.78%)

	**Cytochromes c**				
	Cytochrome c class I		NSCAC _0107	*Halomonas halodenitrificans* ATCC 12084 / NCIMB 8669 (P00105, 39.02 %)	*Nitrosococcus halophilus* Nc4 (D5C293, 55.66%)
	Putative di-heme cytochrome c		NSCAC _0988	*Rhizobium* sp. NGR234 (P55493, 28.08%)	*Nitrosococcus oceani* ATCC 19707 / NCIMB 11848 (Q3JAR1, 63.5%)
	Cytochrome c class I (fragment)		NSCAC _1084	*Chlorobaculum thiosulfatiphilum* (Q8RLX0, 29.2%)	*Nitrosococcus watsoni* C-113 (D8KBT1, 65.35%)
cycA	Cytochrome c class I		NSCAC _1390	*Azotobacter vinelandii* (P43302, 40.91%)	*Nitrosococcus halophilus* Nc4 (D5C0V9, 56.93%)
	Cytochrome c, class I		NSCAC _1724	*Pseudomonas aeruginosa* ATCC 15692 / PAO1 / 1C / PRS 101 / LMG 12228 (P00106, 49.47%)	*Nitrosococcus watsoni* C-113 (D8K4L7, 66.96%)
	Cytochrome c, class I		NSCAC _1725	*Marinobacter hydrocarbonoclasticus* (P82903, 41.67%)	*Nitrosococcus halophilus* Nc4 (D5C491, 69.72%)
	Putative cytochrome c, class I		NSCAC _0748	*Anopheles gambiae* (Q5TWR9, 45.71%)	*Rhodospirillum photometricum* DSM 122 (H6SIA7, 84.62%)
**Nitrogen metabolism**					
	**Ammonia oxidation**				
**Gene**	**Product**	**EC no.**	**CDS[a]**	**Best BLAST hit in SwissProt[b]**	**Best BLAST hit in TrEMBL[c]**
amoB	Ammonia monooxygenase subunit B	1.13.12.-	NSCAC _1277	*Methylococcus capsulatus* ATCC 33009 / NCIMB 11132 / Bath (G1UBD1, 53.56%)	*Nitrosococcus halophilus* Nc4 (D5BWX6, 64.99%)
amoA	Ammonia monooxygenase subunit A	1.13.12.-	NSCAC _1278	*Methylococcus capsulatus* ATCC 33009 / NCIMB 11132 / Bath (Q607G3, 58.92%)	*Nitrosococcus halophilus* Nc4 (D5BWX5, 71.95%)
amoC	Ammonia monooxygenase subunit C	1.13.12.-	NSCAC _1280		*Nitrosococcus oceani* ATCC 19707 / NCIMB 11848 (Q3J890, 74.81%)
amoD	Putative ammonia monooxigenase subunit D		NSCAC _1276		*Nitrosococcus halophilus* strain Nc4 (D5BWX7, 37.7%)
cycX	putative tetraheme cytochrome c		NSCAC _1125	*Nitrosomonas europaea* ATCC 19718 / NBRC 14298 (Q50926, 61.68%)	*Nitrosococcus oceani* AFC27 (B6BZS6, 73.21%)
	conserved protein of unknown function, putative HaoB		NSCAC _1126		*Nitrosococcus halophilus* Nc4 (D5BZ45, 56.61%)
hao	Hydroxylamine dehydrogenase	1.7.2.6	NSCAC _1127	*Nitrosomonas europaea* ATCC 19718 / NBRC 14298 (Q50925, 51.06%)	*Nitrosococcus halophilus* Nc4 (D5BZ44, 73.76%)
	conserved protein of unknown function, putative ammonia/ammonium permease		NSCAC _1037	*Escherichia coli* K12 (P0AA93, 29.49%)	*Nitrosococcus halophilus* Nc4 (D5C4N1, 53.85%)
	Formate/nitrite transporter		NSCAC _1564	*Methanothermobacter thermautotrophicus* (Q50568, 33.46%)	*Nitrosococcus watsoni* C-113 (D8K879, 66.28%)
cyp	Putative Cytochrome P460		NSCAC _1129		*Nitrosococcus halophilus* Nc4 (D5BZ42, 67.78%)
	**Nitric oxide metabolism**				

norB	Nitric oxide reductase	1.7.2.5	NSCAC_1563	*Pseudomonas aeruginosa* ATCC 15692 / PAO1 / 1C / PRS 101 / LMG 12228 (Q59647, 23.77%)	*Rhodanobacter denitrificans* (I4WS67, 65.38%)
	**Urea metabolism**				
	Urea transporter		NSCAC_0475	*Homo sapiens* (Q13336, 30.98%)	*Nitrosococcus oceani* AFC27 (B6C5D8, 58.63%)
ureD	Urease accessory protein UreD		NSCAC_0476	*Nitrosococcus oceani* ATCC 19707 / NCIMB 11848 (Q3J767, 62.77%)	*Nitrosococcus oceani* AFC27 (B6C5D7, 62.77%)
ureA	Urease gamma subunit	3.5.1.5	NSCAC_0477	*Nitrosococcus oceani* ATCC 19707 / NCIMB 11848 (Q3J768, 85.86%)	*Nitrosococcus watsoni* C-113 (D8K8W9, 87.88%)
ureB	Urease beta subunit	3.5.1.5	NSCAC_0478	*Nitrosococcus oceani* ATCC 19707 / NCIMB 11848 (Q3J769, 75.25%)	*Nitrosococcus oceani* AFC27 (B6C5D5, 75.25%)
ureC	Urease alpha subunit	3.5.1.5	NSCAC_0479	*Nitrosococcus oceani* ATCC 19707 / NCIMB 11848 (Q3J770, 77.92%)	*Nitrosococcus watsoni* C-113 (D8K8X1, 77.39%)
ureE	Urease accessory protein UreE		NSCAC_0480	*Nitrosococcus oceani* ATCC 19707 / NCIMB 11848 (Q3J771, 68.71%)	*Nitrosococcus oceani* AFC27 (B6C5D3, 68.71%)
ureF	Urease accessory protein UreF		NSCAC_0481	*Nitrosococcus oceani* ATCC 19707 / NCIMB 11848 (Q3J772, 60.71%)	*Nitrosococcus watsoni* C-113 (D8K8X3, 61.61%)
ureG	Urease accessory protein UreG		NSCAC_0482	*Nitrosococcus oceani* ATCC 19707 / NCIMB 11848 (Q3J773, 84.16%)	*Nitrosococcus watsoni* C-113 (D8K8X4, 84.65%)
**Carbon metabolism**					
	**Carbonate uptake**				
**Gene**	**Product**	**EC no.**	**CDS[a]**	**Best BLAST hit in SwissProt[b]**	**Best BLAST hit in TrEMBL[c]**
can1	Carbonic anhydrase 1	4.2.1.1	NSCAC_0743	*Shigella flexneri* (P61518, 54.37%)	*Pseudoxanthomonas spadix* BD-a59 (G7UV66, 63.11%)
can2	Carbonic anhydrase 2	4.2.1.1	NSCAC_0938	*Mycobacterium tuberculosis* (P64797, 42.31%)	*Nitrosococcus halophilus* Nc4 (D5BZ98, 79.69%)
	**Calvin cycle**				
cbbL	Ribulose bisphosphate carboxylase large chain	4.1.1.39	NSCAC_0587	*Nitrosococcus oceani* ATCC 19707 / NCIMB 11848 (Q3JE87, 92.4%)	*Nitrosococcus watsoni* C-113 (D8KAW1, 93.02%)
cbbS	Ribulose bisphosphate carboxylase small chain	4.1.1.39	NSCAC_0588	*Cupriavidus necator* ATCC 17699 / H16 / DSM 428 / Stanier 337 (Q59102, 70.29%)	*Nitrosococcus halophilus* Nc4 (D5C1B8, 85.42%)
pgk	Phosphoglycerate kinase	2.7.2.3	NSCAC_0211	*Nitrosococcus oceani* ATCC 19707 / NCIMB 11848 (Q3J7E3, 77.44%)	*Nitrosococcus halophilus* Nc4 (D5C1C3, 80.41%)
cbbGP	Glyceraldehyde-3-phosphate dehydrogenase	1.2.1.12	NSCAC_0210	*Cupriavidus necator* ATCC 17699 / H16 / DSM 428 / Stanier 337 (P50322, 74.48%)	*Nitrosococcus halophilus* Nc4 (D5C1C4, 83.68%)
fba	Fructose-bisphosphate aldolase	4.1.2.13	NSCAC_0213	*Pseudomonas stutzeri* (O87796, 76.27%)	*Nitrosococcus halophilus* Nc4 (D5C1C1, 88.7%)
fbp	Fructose-1,6-bisphosphatase I	3.1.3.11	NSCAC_0004	*Nitrosococcus oceani* ATCC 19707 / NCIMB 11848 (Q3JF33, 74.77%)	*Nitrosococcus halophilus* Nc4 (D5C445, 76.28%)

tktA	Transketolase	2.2.1.1	NSCAC _0209	*Escherichia coli* K12 (P27302, 70.48%)	*Nitrosococcus halophilus* Nc4 (D5C1C5, 77.74%)
rpiA	Ribose-5-phosphate isomerase	5.3.1.6	NSCAC _1520	*Nitrosococcus oceani* ATCC 19707 / NCIMB 11848 (Q3J7S6, 85%)	*Nitrosococcus oceani* AFC27 (B6C4Q6, 85%)
prkB	Phosphoribulokinase	2.7.1.19	NSCAC _0197	*Sinorhizobium medicae* WSM419 (P56887, 61.38%)	*Nitrosococcus halophilus* Nc4 (D5C1E0, 86.76%)
tal	Transaldolase	2.2.1.2	NSCAC _0980	*Nostoc* sp. PCC 7120 / UTEX 2576 (P48993, 50.27%)	*Nitrosococcus oceani* ATCC 19707 / NCIMB 11848 (Q3JEE7, 62.16%)
	**Pentose Phosphate pathway**				
zwf	Glucose-6-phosphate 1-dehydrogenase	1.1.1.49	NSCAC _1177	*Synechococcus elongatus* PCC 7942 (P29686, 47.86%)	*Nitrosococcus oceani* ATCC 19707 / NCIMB 11848 (Q3J9H4, 68.32%)
pgl	6-phosphogluconolactonase	3.1.1.31	NSCAC _0942	*Nostoc* sp. PCC 7120 / UTEX 2576 (P46016, 38.24%)	*Nitrosococcus halophilus* Nc4 (D5BZ91, 59.83%)
gnd	6-phosphogluconate dehydrogenase	1.1.1.44	NSCAC _1176	*Lactococcus lactis* subsp. lactis IL1403 (Q9CHU6, 47.69%)	*Nitrosococcus halophilus* Nc4 (D5BY03, 66.67%)
rpe	D-ribulose-5-phosphate 3-epimerase	5.1.3.1	NSCAC _1267	*Haemophilus influenzae* ATCC 51907 / DSM 11121 / KW20 / Rd (P44756, 78.38%)	*Nitrosococcus oceani* AFC27 (B6C0A2, 83.78%)
rpiA	ribose 5-phosphate isomerase A	5.3.1.6	NSCAC _1520	*Nitrosococcus oceani* ATCC 19707 / NCIMB 11848 (Q3J7S6, 85%)	*Nitrosococcus oceani* AFC27 (B6C4Q6, 85%)
tktA	Transketolase	2.2.1.1	NSCAC _0209	*Escherichia coli* K12 (P27302, 70.48%)	*Nitrosococcus halophilus* Nc4 (D5C1C5, 77.74%)
tal	Transaldolase	2.2.1.2	NSCAC _0980	*Nostoc* sp. PCC 7120 / UTEX 2576 (P48993, 50.27%)	*Nitrosococcus oceani* ATCC 19707 / NCIMB 11848 (Q3JEE7, 62.16%)
pgi	Glucose-6-phosphate isomerase	5.3.1.9	NSCAC _1119	*Nitrosococcus oceani* ATCC 19707 / NCIMB 11848 (Q3JCN1, 70.46%)	*Nitrosococcus halophilus* Nc4 (D5BZ55, 73.01%)
fbp	Fructose-1,6-bisphosphatase class 1	3.1.3.11	NSCAC _0004	*Nitrosococcus oceani* ATCC 19707 / NCIMB 11848 (Q3JF33, 74.77%)	*Nitrosococcus halophilus* Nc4 (D5C445, 76.28%)
fba	Fructose-bisphosphate aldolase	4.1.2.13	NSCAC _0213	*Pseudomonas stutzeri* (O87796, 76.27%)	*Nitrosococcus halophilus* Nc4 (D5C1C1, 88.7%)
	**TCA cycle**				
gltA	Citrate synthase	2.3.3.1	NSCAC _1482	*Acidithiobacillus ferrooxidans* (P51045 50.92%)	*Nitrosococcus watsoni* C-113 (D8KAD9, 59.02%)
acnA	Aconitate hydratase	4.2.1.3	NSCAC _0758	*Legionella pneumophila* (P37032, 58.4%)	*Halorhodospira halophila* DSM 244 / SL1 (A1WWW1, 60.57%)
icd	Isocitrate dehydrogenase	1.1.1.42	NSCAC _0452	*Coxiella burnetii* RSA 493 (Q9ZH99, 68.27%)	*Nitrosococcus halophilus* Nc4 (D5BWG2, 74.88%)
sucD	Succinyl-CoA ligase subunit alpha	6.2.1.5	NSCAC _1476	*Rickettsia felis* ATCC VR-1525 / URRWXCal2 (Q4ULQ8, 70%)	*Nitrosococcus halophilus* Nc4 (D5BWC3, 78.2%)
sucC	Succinyl-CoA ligase subunit beta	6.2.1.5	NSCAC _1477	*Thioalkalivibrio* sp. HL-EbGR7 (B8GN38, 55.7%)	*Nitrosococcus halophilus* Nc4 (D5BWC2, 64.68%)
fumC	Fumarate hydratase class II	4.2.1.2	NSCAC _0890	*Coxiella burnetii* RSA 493 (Q83CL8, 61.63%)	*Nitrosococcus watsoni* C-113 (D8K679, 71.03%)

maeB	Malic enzyme	1.1.1.40	NSCAC _0061	*Rhizobium meliloti* 1021 (O30808, 53.25%)	*Nitrosococcus halophilus* Nc4 (D5C3J9, 69.63%)
	**Glycolysis / Gluconeogenesis**				
pgm	Phosphoglucomutase	5.4.2.2	NSCAC _1039	*Rhizobium radiobacter* (P39671, 58.2%)	*Nitrosococcus watsoni* C-113 (D8K5X6, 68.93%)
pgi	Glucose-6-phosphate isomerase	5.3.1.9	NSCAC _1119	*Nitrosococcus oceani* ATCC 19707 / NCIMB 11848 (Q3JCN1, 70.46%)	*Nitrosococcus halophilus* Nc4 (D5BZ55, 73.01%)
pfp	Phosphofructokinase	2.7.1.90	NSCAC _1402	*Dictyoglomus thermophilum* (Q9KH71, 27.49%)	*Nitrosococcus halophilus* Nc4 (D5BW42, 78.31%)
tpiA	Triosephosphate isomerase	5.3.1.1	NSCAC _0233	*Nitrosococcus oceani* ATCC 19707 / NCIMB 11848 (Q3J827, 65.74%)	*Nitrosococcus halophilus* Nc4 (D5C2P7, 66.14%)
gap	Glyceraldheyde-3-phosphate dehydrogenase	1.2.1.12	NSCAC _0210	*Cupriavidus necator* ATCC 17699 / H16 / DSM 428 / Stanier 337 (P50322, 74.48%)	*Nitrosococcus halophilus* Nc4 (D5C1C4, 83.68%)
pgk	Phosphoglycerate kinase	2.7.2.3	NSCAC _0211	*Nitrosococcus oceani* ATCC 19707 / NCIMB 11848 (Q3J7E3, 77.44%)	*Nitrosococcus halophilus* Nc4 (D5C1C3, 80.41%)
eno	Enolase	4.2.1.11	NSCAC _0796	*Nitrosococcus oceani* ATCC 19707 / NCIMB 11848 (Q3JCT1, 71.46%)	*Nitrosococcus halophilus* Nc4 (D5BX72, 73.76%)
pyk	Pyruvate kinase	2.7.1.40	NSCAC _0974	*Bacillus subtilis* 168 (P80885, 39.62%)	*Nitrosococcus watsoni* C-113 (D8K7K1, 62.95%)
glk	Glucokinase	2.7.1.2	NSCAC _0941	*Nostoc punctiforme* ATCC 29133 / PCC 73102 (B2J224, 45.77%)	*Nitrosococcus halophilus* Nc4 (D5BZ92, 68.84%)
fbp	Fructose-1,6-bisphosphatase	3.1.3.11	NSCAC _0004	*Nitrosococcus oceani* ATCC 19707 / NCIMB 11848 (Q3JF33, 74.77%)	*Nitrosococcus halophilus* Nc4 (D5C445, 76.28%)
fba	Fructose-bisphosphate aldolase	4.1.2.13	NSCAC _0213	*Pseudomonas stutzeri* (O87796, 76.27%)	*Nitrosococcus halophilus* Nc4 (D5C1C1, 88.7%)
gpmA	Phosphoglyceromutase	5.4.2.11	NSCAC _0704	*Nitrosococcus oceani* ATCC 19707 / NCIMB 11848 (Q3JBA8, 74.67%)	*Nitrosococcus watsoni* C-113 (D8K6N5, 74.78%)
ppc	Phosphoenolpyruvate carboxylase	4.1.1.31	NSCAC _1317	*Ralstonia solanacearum* GMI1000 (Q8XWW2, 36.33%)	*Nitrosococcus halophilus* Nc4 (D5C581, 72.44%)
	**Carbon storage**				
glgA	Glycogen synthase	2.4.1.21	NSCAC _1316	*Nitrosococcus oceani* ATCC 19707 / NCIMB 11848 (Q3JD12, 61.75%)	*Nitrosococcus halophilus* Nc4 (D5C582, 63.83%)
	**Mannose / Fructose metabolism**				
fbp	Fructose-1,6-bisphosphatase class 1	3.1.3.11	NSCAC _0004	*Nitrosococcus oceani* ATCC 19707 / NCIMB 11848 (Q3JF33, 74.77%)	*Nitrosococcus halophilus* Nc4 (D5C445, 76.28%)
mak	Mannofructokinase	2.7.1.4	NSCAC _0194	*Escherichia coli* K12 (P23917, 55.18%)	*Sulfuricella denitrificans* skB26 (S6B2M3, 67.34%)
fba	Fructose-bisphosphate aldolase	4.1.2.13	NSCAC _0213	*Pseudomonas stutzeri* (O87796, 76.27%)	*Nitrosococcus halophilus* Nc4 (D5C1C1, 88.7%)
tpiA	Triosephosphate isomerase	5.3.1.1	NSCAC _0233	*Nitrosococcus oceani* ATCC 19707 / NCIMB 11848 (Q3J827, 65.74%)	*Nitrosococcus halophilus* Nc4 (D5C2P7, 66.14%)

fcl	GDP-L-fucose synthase	1.1.1.271	NSCAC_0511	*Arabidopsis thaliana* (Q9LMU0, 60.13%)	*Nitrosococcus watsoni* C-113 (D8K579, 75.74%)
gmd	GDP-D-mannose dehydratase	4.2.1.47	NSCAC_0859	*Vibrio cholerae* (Q56598, 69.32%)	*Nitrosococcus watsoni* C-113 (D8K6H6, 80.16%)
manC	Mannose-1-phosphate guanylyltransferase/mannose-6-phosphate isomerase	2.7.7.13, 5.3.1.8	NSCAC_1259	*Xanthomonas campestris* pv. campestris B100 (B0RVK6, 56.07%)	*Nitrosococcus watsoni* C-113 (D8KB73, 68.65%)
mpg1	Mannose-1-phosphate guanyltransferase	2.7.7.13	NSCAC_1309	*Yarrowia lipolytica* CLIB 122 / E 150 (Q6CCU3, 30.74%)	*Nitrosococcus halophilus* Nc4 (D5BV07, 68.84%)
pfp	Phosphofructokinase	2.7.1.90	NSCAC_1402	*Dictyoglomus thermophilum* (Q9KH71, 27.49%)	*Nitrosococcus halophilus* Nc4 (D5BW42, 78.31%)
khk	Ketohexokinase	2.7.1.3	NSCAC_1404	*Homo sapiens* (P50053, 35.84%)	*Nitrosococcus halophilus* Nc4 (D5BW40, 55.21%)
	putative UDP-glucose/GDP-mannose dehydrogenase	1.1.1.22, 1.1.1.132	NSCAC_1492	*Salmonella typhi* (Q04972, 61.85%)	*Nitrosococcus halophilus* Nc4 (D5BW99, 81.88%)
algC	Phosphomannomutase/phosphoglucomutase	5.4.2.2, 5.4.2.8	NSCAC_1698	*Pseudomonas aeruginosa* ATCC 15692 / PAO1 / 1C / PRS 101 / LMG 12228 (P26276, 56.46%)	*Nitrosococcus halophilus* Nc4 (D5C3B0, 68.16%)
**Aminoacid metabolism**					
	**Aspartate / Asparagine / Glutamate**				
**Gene**	**Product**	**EC no.**	**CDS[a]**	**Best BLAST hit in SwissProt[b]**	**Best BLAST hit in TrEMBL[c]**
carA	Carbamoyl-phosphate synthase, small subunit	6.3.5.5	NSCAC_0226	*Thioalkalivibrio* sp. HL-EbGR7 (B8GNX4, 66.22%)	*Nitrosococcus halophilus* Nc4 (D5C2Q5, 73.49%)
carB	Carbamoyl-phosphate synthase, large subunit	6.3.5.5	NSCAC_0227	*Pseudomonas syringae* pv. tomato DC3000 (Q87WP4, 69.71%)	*Nitrosococcus halophilus* Nc4 (D5C2Q4, 79.79%)
asnB	Asparagine synthetase	6.3.5.4	NSCAC_1330	*Bacillus subtilis* 168 (P54420, 26.61%)	*Sulfuricella denitrificans* skB26 (S6B8Y8, 46.02%)
aspC	Aspartate aminotransferase	2.6.1.1	NSCAC_1014	*Rickettsia bellii* RML369-C (Q1RGV0, 53.71%)	*Nitrosococcus halophilus* Nc4 (D5C014, 75.57%)
asd	Aspartate-semialdehyde dehydrogenase	1.2.1.11	NSCAC_1076	*Legionella pneumophila* (O31219, 65%)	*Nitrosococcus halophilus* Nc4 (D5C3U9, 79.71%)
pyrB	Aspartate carbamoyltransferase	2.1.3.2	NSCAC_0268	*Nitrosococcus oceani* ATCC 19707 / NCIMB 11848 (Q3JE55, 71.79%)	*Nitrosococcus halophilus* Nc4 (D5C4Z0, 73.31%)
panD	Aspartate 1-decarboxylase	4.1.1.11	NSCAC_1130	*Nitrosococcus oceani* ATCC 19707 / NCIMB 11848 (Q3JCP7, 70.09%)	*Nitrosococcus halophilus* Nc4 (D5BZ33, 70.94%)
glnA	Glutamine synthetase	6.3.1.2	NSCAC_1505	*Methylococcus capsulatus* ATCC 33009 / NCIMB 11132 / Bath (P15124, 75.69%)	*Nitrosococcus halophilus* Nc4 (D5BW85, 84.65%)
gdh	Glutamate dehydrogenase	1.4.1.4	NSCAC_0541	*Corynebacterium glutamicum* ATCC 13032 / DSM 20300 / JCM 1318 / LMG 3730 / NCIMB 10025 (P31026, 65.23%)	*Nitrosococcus watsoni* C-113 (D8K8F3, 76.46%)
proB	Glutamate 5-kinase	2.7.2.11	NSCAC_1710	*Nitrosococcus oceani* ATCC 19707 / NCIMB 11848 (Q3J6R9, 68.27%)	*Nitrosococcus halophilus* Nc4 (D5C4A5, 67.73%)
mptN	Alpha-L-glutamate ligase	6.3.2.33	NSCAC_0081	*Methanocaldococcus jannaschii* ATCC 43067 / DSM 2661 / JAL-1 / JCM 10045 / NBRC 100440 (Q58037, 33.45%)	*Nitrosococcus halophilus* Nc4 (D5C3I2, 58.92%)

gltX	Glutamate-tRNA ligase	6.1.1.17	NSCAC _0538	*Nitrosococcus oceani* ATCC 19707 / NCIMB 11848 (Q3J8Y8, 63.73%)	*Nitrosococcus halophilus* Nc4 (D5BYC6, 64.87%)
gshA	Glutamate-cysteine ligase	6.3.2.2	NSCAC _0994	*Alcanivorax borkumensis* SK2 / ATCC 700651 / DSM 11573 (Q0VSY7, 46.93%)	*Nitrosococcus halophilus* Nc4 (D5BZX8, 62.96%)
hemL	Glutamate-1-semialdehyde aminotransferase	5.4.3.8	NSCAC _1655	*Nitrosococcus oceani* ATCC 19707 / NCIMB 11848 (Q3JDH7, 75.24%)	*Nitrosococcus halophilus* Nc4 (D5C2V8, 75.82%)
glnE	Glutamate-ammonia-ligase adenylyltransferase	2.7.7.42	NSCAC _1683	*Nitrosococcus oceani* ATCC 19707 / NCIMB 11848 (Q3JES8, 55.33%)	*Nitrosococcus halophilus* Nc4 (D5C4W0, 55.97%)
argB	Acetylglutamate kinase	2.7.2.8	NSCAC _1697	*Nitrosococcus oceani* ATCC 19707 / NCIMB 11848 (Q3J6V9, 75%)	*Nitrosococcus oceani* AFC27 (B6C5S6, 75%)
	**Glycine / Serine / Threonine**				
hprA	Glycerate dehydrogenase	1.1.1.29	NSCAC _0419	*Methylobacterium extorquens* ATCC 14718 / DSM 1338 / AM1 (Q59516, 43.95%)	*Nitrosococcus halophilus* Nc4 (D5BXV7, 68.55%)
GLYK	D-glycerate 3-kinase	2.7.1.31	NSCAC _0567	*Schizosaccharomyces pombe* 972 / ATCC 24843 (Q9UUH3, 32.08)	*Nitrosococcus oceani* ATCC 19707 / NCIMB 11848 (Q3JDS9, 48.74%)
serA	D-3-phosphoglycerate dehydrogenase	1.1.1.95	NSCAC _0879	*Methanocaldococcus jannaschii* ATCC 43067 / DSM 2661 / JAL-1 / JCM 10045 / NBRC 100440 (Q58424, 37.14%)	*Nitrosococcus halophilus* Nc4 (D5BWP9, 71.06%)
serB	Phosphoserine phosphatase	3.1.3.3	NSCAC _1281	*Haemophilus influenzae* ATCC 51907 / DSM 11121 / KW20 / Rd (P44997, 42.96%)	*Nitrosococcus oceani* ATCC 19707 / NCIMB 11848 (Q3J889, 56.43%)
serC	Phosphoserine aminotransferase	2.6.1.52	NSCAC _0878	*Thiobacillus denitrificans* ATCC 25259 (Q3SK88, 64.8%)	*Nitrosococcus halophilus* Nc4 (D5BWP8, 73.89%)
hom	Homoserine dehydrogenase	1.1.1.3	NSCAC _1243	*Pseudomonas aeruginosa* ATCC 15692 / PAO1 / 1C / PRS 101 / LMG 12228 (P29365, 62.82%)	*Nitrosococcus halophilus* Nc4 (D5BX24, 75.06%)
psd	Phosphatidylserine decarboxylase proenzyme	4.1.1.65	NSCAC _0144	*Nitrosococcus oceani* ATCC 19707 / NCIMB 11848 (Q3J754, 60.96%)	*Nitrosococcus halophilus* Nc4 (D5BVJ8, 63.7%)
metA	Homoserine O-succinyltransferase	2.3.1.46	NSCAC _0619	*Vibrio fischeri* MJ11 (B5FA49, 34.22%)	*Nitrosococcus halophilus* Nc4 (D5C1M0, 78.03%)
metZ	O-succinylhomoserine sulfhydrylase	2.5.1.-	NSCAC _1004	*Pseudomonas aeruginosa* ATCC 15692 / PAO1 / 1C / PRS 101 / LMG 12228 (P55218, 61.66%)	*Nitrosococcus halophilus* Nc4 (D5BZZ8, 79.8%)
glyA	Serine hydroxymethyltransferase	2.1.2.1	NSCAC _0423	*Nitrosococcus oceani* ATCC 19707 / NCIMB 11848 (Q3J9K8, 83.69%)	*Nitrosococcus watsoni* C-113 (D8K532, 84.17%)
	Aspartate/glutamate/uridylate kinase		NSCAC _0076	*Arabidopsis thaliana* (Q93Y07, 31.37%)	*Nitrosococcus halophilus* Nc4 (D5C3I9, 51.83%)
asd	Aspartate-semialdehyde dehydrogenase	1.2.1.11	NSCAC _1076	*Legionella pneumophila* (O31219, 65%)	*Nitrosococcus halophilus* Nc4 (D5C3U9, 79.71%)
thrC	Threonine synthase	4.2.3.1	NSCAC _1242	*Synechocystis* sp. PCC 6803 / Kazusa (P74193, 56.12%)	*Nitrosococcus halophilus* Nc4 (D5BX25, 77.99%)
	**Cysteine / Methionine**				
cysE	Serine acetyltransferase	2.3.1.30	NSCAC _0752	*Azotobacter chroococcum* mcd 1 (P23145, 53.24%)	*Nitrosococcus halophilus* Nc4 (D5C3R4, 69.5%)
cysM	Cysteine synthase	2.5.1.47	NSCAC _0395	*Pseudomonas aeruginosa* ATCC 15692 / PAO1 / 1C / PRS 101 / LMG 12228 (Q9I526, 66.44%)	*Nitrosococcus watsoni* C-113 (D8K8M1, 76.61%)

iscS	Cysteine desulfurase	2.8.1.7	NSCAC _0753	*Dictyoglomus turgidum* Z-1310 / DSM 6724 (B8DZS1, 44.3%)	*Nitrosococcus watsoni* C-113 (D8K661, 64.29%)
icmt	Isoprenylcysteine carboxyl methyltransferase	2.1.1.100	NSCAC _1030	*Alcaligenes xylosoxydans* (Q44587, 28.41%)	*Nitrosococcus halophilus* Nc4 (D5C4M2, 57.75%)
metE	5-methyltetrahydropteroyltriglutamate-homocysteine S-methyltransferase	2.1.1.14	NSCAC _0673	*Nitrosococcus oceani* ATCC 19707 / NCIMB 11848 (Q3JAD5, 72.66%)	*Nitrosococcus halophilus* Nc4 (D5C080, 73.18%)
metK	S-adenosylmethionine synthase	2.5.1.6	NSCAC _1528	*Nitrosococcus oceani* ATCC 19707 / NCIMB 11848 (Q3J7R5, 83.51%)	*Nitrosococcus halophilus* Nc4 (D5BW58, 84.79%)
	**Valine / Leucine / Isoleucine**				
CHA1	L-threonine ammonia-lyase	4.3.1.19	NSCAC _1519	*Burkholderia multivorans* ATCC 17616 / 249 (P53607, 56.04%)	*Nitrosococcus halophilus* Nc4 (D5BW70, 75.51%)
ilvC	Ketol-acid reductoisomerase	1.1.1.86	NSCAC _1286	*Nitrosococcus oceani* ATCC 19707 / NCIMB 11848 (Q3J875, 85.5%)	*Nitrosococcus oceani* AFC27 (B6C687, 85.5%)
ilvH	Acetolactate synthase, small subunit	2.2.1.6	NSCAC _1287	*Escherichia coli* K12 (P00894, 56.71%)	*Nitrosococcus halophilus* Nc4 (D5BWV5, 80.37%)
ilvI	Acetolactate synthase, large subunit	2.2.1.6	NSCAC _1288	*Haemophilus influenzae* ATCC 51907 / DSM 11121 / KW20 / Rd (P45261, 57.88%)	*Nitrosococcus halophilus* Nc4 (D5BWV4, 76.75%)
ilvD	Dihydroxy-acid dehydratase	4.2.1.9	NSCAC _1351	*Nitrosococcus oceani* ATCC 19707 / NCIMB 11848 (Q3J9N3, 82.47%)	*Nitrosococcus watsoni* C-113 (D8K6Y1, 82.63%)
ilvE	Branched-chain-amino-acid aminotransferase	2.6.1.42	NSCAC _1682	*Pseudomonas aeruginosa* ATCC 15692 / PAO1 / 1C / PRS 101 / LMG 12228 (O86428, 67.33%)	*Nitrosococcus watsoni* C-113 (D8K8A8, 81.55%)
leuA	2-isopropylmalate synthase	2.3.3.13	NSCAC _1284	*Nitrosococcus oceani* ATCC 19707 / NCIMB 11848 (Q3J877, 75.54%)	*Nitrosococcus halophilus* Nc4 (D5BWV8, 76.91%)
leuB	3-isopropylmalate dehydrogenase	1.1.1.85	NSCAC _1078	*Nitrosococcus oceani* ATCC 19707 / NCIMB 11848 (Q3JCC4, 78.65%)	*Nitrosococcus watsoni* C-113 (D8K7H6, 79.55%)
leuD	3-isopropylmalate dehydratase, small subunit	4.2.1.33	NSCAC _1079	*Nitrosococcus oceani* ATCC 19707 / NCIMB 11848 (Q3JCC5, 69.95%)	*Nitrosococcus halophilus* Nc4 (D5C3U7, 71.83%)
leuC	3-isopropylmalate dehydratase, large subunit	4.2.1.33	NSCAC _0131	*Nitrosococcus oceani* ATCC 19707 / NCIMB 11848 (Q3J716, 82.66%)	*Nitrosococcus halophilus* Nc4 (D5BUS1, 83.08%)
	**Lysine / Arginine / Proline**				
lysA	Diaminopimelate decarboxylase	4.1.1.20	NSCAC _0103	*Vibrio cholerae* serotype O1 ATCC 39315 / El Tor Inaba N16961 (Q9KVL7, 63.88%)	*Nitrosococcus oceani* AFC27 (B6C3A1, 70.84%)
dapF	Diaminopimelate epimerase	5.1.1.7	NSCAC _0105	*Nitrosococcus oceani* ATCC 19707 / NCIMB 11848 (Q3JEA2, 63.04%)	*Nitrosococcus halophilus* Nc4 (D5C3E8, 65.58%)
dapE	Succinyl-diaminopimelate desuccinylase	3.5.1.18	NSCAC _0570	*Nitrosococcus oceani* ATCC 19707 / NCIMB 11848 (Q3J9B0, 66.13%)	*Nitrosococcus halophilus* Nc4 (D5BXS2, 66.93%)
dapB	4-hydroxy-tetrahydrodipicolinate reductase	1.17.1.8	NSCAC _0208	*Nitrosococcus oceani* ATCC 19707 / NCIMB 11848 (Q3J7E0, 61.36%)	*Nitrosococcus watsoni* C-113 (D8K9G0, 64.39%)
dapD	2,3,4,5-tetrahydropyridine-2,6-dicarboxylate N-succinyltransferase	2.3.1.117	NSCAC _0571	*Nitrosococcus oceani* ATCC 19707 / NCIMB 11848 (Q3J9B1, 78.39%)	*Nitrosococcus halophilus* Nc4 (D5BXS3, 79.12%)
asd	Aspartate-semialdehyde dehydrogenase	1.2.1.11	NSCAC _1076	*Legionella pneumophila* (O31219, 65%)	*Nitrosococcus halophilus* Nc4 (D5C3U9, 79.71%)

lysC	Aspartate kinase	2.7.2.4	NSCAC _1101	*Pseudomonas aeruginosa* ATCC 15692 / PAO1 / 1C / PRS 101 / LMG 12228 (O69077, 71.57%)	*Nitrosococcus halophilus* Nc4 (D5BZJ7, 78.92%)
hom	Homoserine dehydrogenase	1.1.1.3	NSCAC _1243	*Pseudomonas aeruginosa* ATCC 15692 / PAO1 / 1C / PRS 101 / LMG 12228 (P29365, 62.82%)	*Nitrosococcus halophilus* Nc4 (D5BX24, 75.06%)
alaC	Aspartate aminotransferase	2.6.1.1	NSCAC _1244	*Escherichia coli* K12 (P77434, 69.95%)	*Nitrosococcus oceani* ATCC 19707 / NCIMB 11848 (Q3J8D8, 84.14%)
murF	UDP-N-acetylmuramoyl-tripeptide:D-alanyl-D-alanine ligase	6.3.2.10	NSCAC _1421	*Escherichia coli* K12 (P11880, 41.48%)	*Nitrosococcus halophilus* Nc4 (D5BW22, 56.88%)
murE	UDP-N-acetylmuramoyl-L-alanyl-D-glutamate--2,6-diaminopimelate ligase	6.3.2.13	NSCAC _1422	*Coxiella burnetii* RSA 493 (Q83F28, 42.07%)	*Nitrosococcus watsoni* C-113 (D8K8Y2, 55.73%)
proA	Gamma-glutamyl phosphate reductase	1.2.1.41	NSCAC _1515	*Nitrosococcus oceani* ATCC 19707 / NCIMB 11848 (Q3J7T1, 72.73%)	*Nitrosococcus halophilus* Nc4 (D5BW74, 74.4%)
proC	Pyrroline-5-carboxylate reductase	1.5.1.2	NSCAC _1692	*Pseudomonas aeruginosa* ATCC 15692 / PAO1 / 1C / PRS 101 / LMG 12228 (P22008, 44.4%)	*Nitrosococcus oceani* AFC27 (B6C5T3, 61.07%)
proB	Glutamate 5-kinase	2.7.2.11	NSCAC _1710	*Nitrosococcus oceani* ATCC 19707 / NCIMB 11848 (Q3J6R9, 68.27%)	*Nitrosococcus halophilus* Nc4 (D5C4A5, 67.73%)
argH	Argininosuccinate lyase	4.3.2.1	NSCAC _1756	*Nitrosococcus oceani* ATCC 19707 / NCIMB 11848 (Q3JDS2, 80.17%)	*Nitrosococcus watsoni* C-113 (D8KA13, 79.7%)
	Arginine decarboxylase		NSCAC _0500		*Nitrosococcus halophilus* Nc4 (D5BWL0, 47.44%)
argJ	Arginine biosynthesis bifunctional protein ArgJ	2.3.1.1, 2.3.1.35	NSCAC _1406	*Nitrosococcus oceani* ATCC 19707 / NCIMB 11848 (Q3J7A0, 65.19%)	Nitrosococcus halophilus Nc4 (D5BW38, 65.43%)
	**Histidine**				
hisC	Histidinol-phosphate aminotransferase	2.6.1.9	NSCAC _1567	*Nitrosococcus oceani* ATCC 19707 / NCIMB 11848 (Q3J7H2, 66.57%)	*Nitrosococcus oceani* AFC27 (B6C529, 66.57%)
hisD	Histidinol dehydrogenase	1.1.1.23	NSCAC _1568	*Nitrosococcus oceani* ATCC 19707 / NCIMB 11848 (Q3J7H1, 73.26%)	*Nitrosococcus halophilus* Nc4 (D5BVB8, 73.26%)
hisG	ATP phosphoribosyltransferase	2.4.2.17	NSCAC _1569	*Nitrosococcus oceani* ATCC 19707 / NCIMB 11848 (Q3J7H0, 75.12%)	*Nitrosococcus watsoni* C-113 (D8K9J0, 75.59%)
hisE	Phosphoribosyl-ATP pyrophosphatase/Phosphoribosyl-AMP cyclohydrolase	3.6.1.31, 3.5.4.19	NSCAC _1731	*Nitrosococcus oceani* ATCC 19707 / NCIMB 11848 (Q3J6P9, 66.99%)	*Nitrosococcus halophilus* Nc4 (D5C484, 70.87%)
hisIE	Phosphoribosyl-AMP cyclohydrolase/phosphoribosyl-ATP pyrophosphatase	3.5.4.19, 3.6.1.31	NSCAC _1730	*Nitrosococcus oceani* ATCC 19707 / NCIMB 11848 (Q3J6Q0, 66.67%)	*Nitrosococcus halophilus* Nc4 (D5C485, 65.89%)
hisB	Imidazoleglycerol-phosphate dehydratase	4.2.1.19	NSCAC _1726	*Nitrosococcus oceani* ATCC 19707 / NCIMB 11848 (Q3J6Q4, 72.82%)	*Nitrosococcus halophilus* Nc4 (D5C489, 77.33%)
hisH	Imidazole glycerol phosphate synthase, subunit HisH	2.4.2.-	NSCAC _1727	*Nitrosococcus oceani* ATCC 19707 / NCIMB 11848 (Q3J6Q3, 65.88%)	*Nitrosococcus halophilus* Nc4 (D5C488, 67.77%)
hisA	Phosphoribosylformimino-5-aminoimidazole carboxamide ribotide isomerase	5.3.1.16	NSCAC _1728	*Nitrosococcus oceani* ATCC 19707 / NCIMB 11848 (Q3J6Q2, 80.08%)	*Nitrosococcus halophilus* Nc4 (D5C487, 81.33%)
hisF	Imidazole glycerol phosphate synthase, subunit HisF	4.1.3.-	NSCAC _1729	*Nitrosococcus oceani* ATCC 19707 / NCIMB 11848 (Q3J6Q1, 79.3%)	*Nitrosococcus halophilus* Nc4 (D5C486, 81.32%)

	**Phenylalanine / Tyrosine / Tryptophan**				
aroG	Phospho-2-dehydro-3-deoxyheptonate aldolase	2.5.1.54	NSCAC_0871	*Escherichia coli* K12 (P0AB91, 60.17%)	*Nitrosococcus halophilus* Nc4 (D5BWN5, 72.78%)
aroB	3-dehydroquinate synthase	4.2.3.4	NSCAC_0070	*Nitrosococcus oceani* ATCC 19707 / NCIMB 11848 (Q3JEG3, 69.38%)	*Nitrosococcus halophilus* Nc4 (D5C3J0, 71.07%)
aroQ	3-dehydroquinate dehydratase	4.2.1.10	NSCAC_1548	*Nitrosococcus oceani* ATCC 19707 / NCIMB 11848 (Q3JC85, 70.5%)	*Nitrosococcus watsoni* C-113 (D8K7D6, 71.94%)
aroE	Shikimate dehydrogenase	1.1.1.25	NSCAC_0224	*Nitrosococcus oceani* ATCC 19707 / NCIMB 11848 (Q3J818, 60.9%)	*Nitrosococcus watsoni* C-113 (D5C2Q7, 61.28%)
aroK	Shikimate kinase	2.7.1.71	NSCAC_0069	*Nitrosococcus oceani* ATCC 19707 / NCIMB 11848 (Q3JEG4, 59.17%)	*Nitrosococcus oceani* AFC27 (B6C338, 59.17%)
tyrA	Prephenate dehydrogenase	1.3.1.12	NSCAC_0882	*Zymomonas mobilis* subsp. mobilis ATCC 31821 / ZM4 / CP4 (Q04983, 41.13%)	*Nitrosococcus oceani* AFC27 (B6C2V5, 56.89%)
aroA	3-phosphoshikimate 1-carboxyvinyltransferase	2.5.1.19	NSCAC_0883	*Nitrosococcus oceani* ATCC 19707 / NCIMB 11848 (Q3JEN6, 70.67%)	*Nitrosococcus halophilus* Nc4 (D5BX27, 71.59%)
aroC	Chorismate synthase	4.2.3.5	NSCAC_1591	*Nitrosococcus oceani* ATCC 19707 / NCIMB 11848 (Q3J713, 82.05%)	*Nitrosococcus halophilus* Nc4 (D5BUS5, 82.05%)
alaC	Aspartate aminotransferase	2.6.1.1	NSCAC_1244	*Escherichia coli* K12 (P77434, 69.95%)	*Nitrosococcus oceani* ATCC 19707 / NCIMB 11848 (Q3J8D8, 84.14%)
hisC	Histidinol-phosphate aminotransferase	2.6.1.9	NSCAC_1567	*Nitrosococcus oceani* ATCC 19707 / NCIMB 11848 (Q3J7H2, 66.57%)	*Nitrosococcus oceani* AFC27 (B6C529, 66.57%)
pheA	Chorismate mutase / Prephenate dehydratase	5.4.99.5, 4.2.1.51	NSCAC_0880	*Pseudomonas stutzeri* (P27603, 52.49%)	*Nitrosococcus watsoni* C-113 (D8K8T7, 68.96%)
trpA	Tryptophan synthase alpha chain	4.2.1.20	NSCAC_1067	*Nitrosococcus oceani* ATCC 19707 / NCIMB 11848 (Q3JCB7, 69.55%)	*Nitrosococcus oceani* AFC27 (B6C0H6, 69.55%)
trpB	Tryptophan synthase beta chain	4.2.1.20	NSCAC_1068	*Methylococcus capsulatus* ATCC 33009 / NCIMB 11132 / Bath (Q604P3, 75.19%)	*Nitrosococcus oceani* AFC27 (B6C0H5, 80.9%)
**Hydrogen metabolism**					
**Gene**	**Product**	**EC no.**	**CDS[a]**	**Best BLAST hit in SwissProt[b]**	**Best BLAST hit in TrEMBL[c]**
	putative [NiFe]-hydrogenase, small subunit HyfI		NSCAC_1085	*Escherichia coli* K12 (P16433, 42.25%)	*Afipia* sp. 1NLS2 (D6V096, 79.52%)
	putative [NiFe]-hydrogenase, large subunit HyfG		NSCAC_1086	*Escherichia coli* K12 (P16431, 31.96%)	*Afipia* sp. 1NLS2 (D6V095, 74.9%)
	putative [NiFe]-hydrogenase, membrane subunit HyfF		NSCAC_1087	*Methylacidiphilum infernorum* isolate V4 (A9QPJ1, 58.99%)	*Afipia* sp. 1NLS2 (D6V094, 76.36%)
	putative [NiFe]-hydrogenase, membrane subunit HyfE		NSCAC_1088	*Mycobacterium tuberculosis* (P64681, 28.37%)	*Afipia* sp. 1NLS2 (D6V093, 78.9%)
	putative [NiFe]-hydrogenase, membrane subunit HyfC		NSCAC_1089	*Escherichia coli* K12 (P77858, 33.78%)	*Afipia* sp. 1NLS2 (D6V092, 79.94%)
	putative [NiFe]-hydrogenase, membrane subunit HyfB		NSCAC_1090	*Mycobacterium tuberculosis* (Q10880, 34.32%)	*Afipia* sp. 1NLS2 (D6V091, 71.71%)

Gene	Product	EC no.	CDS[a]	Best BLAST hit in SwissProt[b]	Best BLAST hit in TrEMBL[c]
**Sulfur metabolism**					
	**Assimilatory sulfate reduction**				
**Gene**	**Product**	**EC no.**	**CDS[a]**	**Best BLAST hit in SwissProt[b]**	**Best BLAST hit in TrEMBL[c]**
cysC	Adenylyl-sulfate kinase	2.7.1.25	NSCAC _1258	*Acaryochloris marina* MBIC 11017 (B0CAX3, 64%)	*Nitrosococcus halophilus* Nc4 (D5BWZ5, 71.07%)
cysH	5'-adenylylsulfate reductase (thioredoxin)	1.8.4.8	NSCAC _0382	*Burkholderia cepacia* (Q9RFS6, 51.58%)	*Nitrosococcus oceani* ATCC 19707 / NCIMB 11848 (Q3J8U8, 70.48%)
cysD	Sulfate adenylyltransferase, small subunit	2.7.7.4	NSCAC _0383	*Mycobacterium* sp. strain JLS (A3Q3I2, 65.55%)	*Nitrosococcus watsoni* C-113 (D8K7S8, 86.38%)
cysN	Sulfate adenylyltransferase, large subunit	2.7.7.4	NSCAC _0384	*Mycobacterium tuberculosis* (Q10600, 57.38%)	*Nitrosococcus halophilus* Nc4 (D5BVT0, 80.19%)
cysJ	Sulfite reductase [NADPH] flavoprotein, alpha subunit	1.8.1.2, 1.5.1.29	NSCAC _0552	*Bacillus subtilis* 168 (O32214, 52.18%)	*Nitrosococcus watsoni* C-113 (D8K5E5, 69.71%)
cysI	Sulfite reductase [NADPH] hemoprotein, beta subunit	1.8.1.2	NSCAC _0553	*Nitrosococcus oceani* ATCC 19707 / NCIMB 11848 (Q3JBJ1, 74.14%)	*Nitrosococcus watsoni* C-113 (D8K5E6, 74.31%)
	Sulfate transporter / antisigma-factor antagonist STAS		NSCAC _1572	*Bacillus subtilis* 168 (P17903, 30%)	*Nitrosococcus halophilus* Nc4 (D5BVB3, 49.48%)
**Transporters**					
**Gene**	**Product**	**EC no.**	**CDS[a]**	**Best BLAST hit in SwissProt[b]**	**Best BLAST hit in TrEMBL[c]**
	Heavy metal transport/detoxification protein		NSCAC _1686	*Staphylococcus haemolyticus* (Q4L971, 35.4%)	*Nitrosococcus halophilus* Nc4 (D5C4V6, 67.7%)
cutA	Divalent-cation tolerance protein CutA		NSCAC _1545	*Thermus thermophilus* (Q7SIA8, 42.7%)	*Nitrosococcus oceani* ATCC 19707 / NCIMB 11848 (Q3JC82, 58.88%)
czcA	Cation efflux system protein CzcA		NSCAC _0713	*Alcaligenes sp.* (strain CT14) (P94177, 40.17%)	*Candidatus* Nitrosoglobus terrae (A0A1Q2SN03, 75.7%)
	Sodium/hydrogen exchanger		NSCAC _1736	*Escherichia coli* (strain K12) (P45522, 25.57%)	*Nitrosococcus halophilus* Nc4 (D5C477, 63.65%)
yfeH	putative sodium/bile acid symporter family (mazG-like)		NSCAC _0057	*Pseudomonas aeruginosa* (P39879, 52.81%)	*gamma proteobacterium HdN1* (E1VNE1, 6961.85%)
trkH	potassium transporter		NSCAC _0059	*Nitrosococcus oceani* ATCC 19707 / NCIMB 11848 (Q87TN7, 53.97%)	*Nitrosococcus oceani* ATCC 19707 / NCIMB 11848 (A0A0E2Z5T5, 70%)
trkA	NAD-binding component of TrK potassium transporter		NSCAC _1602	*Halomonas elongata* (E1V6C6, 61.4%)	*Nitrosococcus halophilus* Nc4 (D5C3D0, 75.93%)
	Iron permease FTR1		NSCAC _0606	*Bacillus subtilis* (P39595, 27.68%)	*Candidatus* Nitrosoglobus terrae (A0A1Q2SNC9, 63.67%)
	copper/silver efflux system, membrane component		NSCAC _0608	*Escherichia coli* (Q8FK36, 45.97%)	*Candidatus* Nitrosoglobus terrae (A0A1Q2SN97, 68.7%)
corA	Magnesium and cobalt transport		NSCAC _0600	*Thermotoga maritima* (Q9WZ31, 43.1%)	*Nitrosococcus halophilus* Nc4 (D5C2B9, 61.27%)

	MotA/TolQ/ExbB proton channel		NSCAC_1649	*Synechocystis sp.* (strain PCC 6803 / Kazusa) (P72604, 38.89%)	*Candidatus* Nitrosoglobus terrae (A0A1Q2SKN4, 75.88%)
	Efflux transporter, RND family, MFP subunit		NSCAC_0609	*Salmonella typhimurium* (Q9ZHD0, 28.53%)	*Candidatus* Nitrosoglobus terrae (A0A1Q2SND8, 56.38%)

Table S8. Transcriptomics analysis of "Ca. N. tergens RJ19" grown on NH_3 and urea. Values are expressed as RPKM and they represent the average over three samples. Sd: standard deviation.

**Locus_tag**	**Gene**	**Product**	**NH_3**	**Urea**	**sd NH_3**	**sd urea**	**log2 Fold change**	**Pvalue**	**Padj**
NSCAC_1280	amoC	Ammonia monooxygenase subunit C	333276.3	281512.7	44135.7	46894.8	-1	0.02	0.09
NSCAC_1538		conserved exported protein of unknown function	50564.3	74270	8392.4	25928.9	0	0.74	0.86
NSCAC_1278	amoA	Ammonia monooxygenase subunit A	39278.4	19830.5	2666.3	806	-1	0	0
NSCAC_1277	amoB	Ammonia monooxygenase subunit B	38123.4	18143.5	3609.9	2050.1	-2	0	0
NSCAC_0496		conserved protein of unknown function	19015.8	15737.9	4288.9	3558.8	-1	0.02	0.09
NSCAC_0045		conserved exported protein of unknown function	15642.7	11313	7510.3	8227.9	-1	0.04	0.12
NSCAC_0627		protein of unknown function	12418.1	22022.9	4244.4	10397	0	0.24	0.46
NSCAC_1718	ctaE	Cytochrome c oxidase, subunit III	12007.9	7907.3	1002.2	1888.8	-1	0	0
NSCAC_0046		exported protein of unknown function	9312.6	8376.1	5424.3	5974.5	-1	0.19	0.41
NSCAC_0107		Cytochrome c class I	7732	8052.2	1664.2	2508	0	0.27	0.5
NSCAC_1721	ctaD	Cytochrome c oxidase, subunit I	7570.8	3279.1	459.9	67.4	-2	0	0
NSCAC_0629		Chain A, Red Copper Protein Nitrosocyanin	6783.8	4283.8	2445.5	1834.3	-1	0	0
NSCAC_1037		conserved protein of unknown function, putative ammonia/ammonium permease	6628.9	52550.8	251.1	17114.9	3	0	0
NSCAC_0173		putative cold shock protein (CspA-like)	6418.2	8391.2	1319.4	1809.7	0	0.84	0.92
NSCAC_0754		conserved membrane protein of unknown function	6022.3	6026.7	875	291.2	0	0.04	0.13
NSCAC_0844		exported protein of unknown function	5988	3000.9	724.3	78.8	-1	0	0
NSCAC_1722	ctaC	Cytochrome c oxidase subunit II	5979	2662.4	976.7	316.6	-2	0	0
NSCAC_0034		conserved exported protein of unknown function	5741.8	4890.4	2162.7	3781.5	-1	0.11	0.27
NSCAC_0116		conserved exported protein of unknown function	5647.6	4271.9	1028.3	177.1	-1	0	0

NSCAC_1276		conserved exported protein of unknown function	5282.3	4021	428.9	111.5	-1	0	0
NSCAC_0816		putative peroxiredoxin	5263.1	3887.4	213.5	227	-1	0	0
NSCAC_1539	smbP	Metal-binding protein SmbP	4687	9323.1	219.1	2518.9	1	0.05	0.16
NSCAC_0370	rpsE	30S ribosomal protein S5	4583.6	2544.7	1564	706.1	-1	0	0
NSCAC_0137	coxA	Cytochrome c oxidase, subunit I	4553.9	1306	166.1	51.6	-2	0	0
NSCAC_1125	cycX	putative tetraheme cytochrome c	4369.5	3267.7	665.1	792.5	-1	0	0
NSCAC_0136	coxB	Cytochrome c oxidase, subunit II	3954.1	1216.2	671.5	263.8	-2	0	0
NSCAC_0972		conserved exported protein of unknown function	3502.1	7215.4	211.2	74.6	1	0	0
NSCAC_0345	rplL	50S ribosomal subunit protein L7/L12	3397.4	3928.5	746.4	1070.9	0	0.24	0.47
NSCAC_1662	rbpD	putative RNA-binding protein RbpD	3233	3915.1	1021.8	1450.7	0	0.53	0.73
NSCAC_0032	groEL	chaperone Hsp60, peptide-dependent ATPase, heat shock protein	3187.2	3771.4	74.7	207.8	0	0.17	0.38
NSCAC_1719	ctaG	Cytochrome c oxidase assembly protein CtaG	3020.6	1040.4	274.1	261.4	-2	0	0
NSCAC_1127	hao	Hydroxylamine oxidoreductase	3015.8	2291.5	453.8	497.3	-1	0	0
NSCAC_0588	cbbS	Ribulose bisphosphate carboxylase small chain	2972.9	841.4	533.7	91.2	-2	0	0
NSCAC_0118		conserved exported protein of unknown function	2906.6	1495.5	345.7	192.7	-1	0	0
NSCAC_0497		conserved exported protein of unknown function	2403.6	2538.1	624.1	189.1	0	0.07	0.21
NSCAC_0587	cbbL	Ribulose bisphosphate carboxylase large chain	2380.9	611.8	105.6	19.3	-2	0	0
NSCAC_0592		Glutathione S-transferase, N-terminal domain	2231.6	1246.5	257.9	110.8	-1	0	0
NSCAC_0368	rplF	50S ribosomal subunit protein L6	2200.8	2511.5	76.3	423.5	0	0.08	0.23
NSCAC_0187		protein of unknown function	2107.2	1244.8	580.2	179.7	-1	0	0
NSCAC_1564		Formate/nitrite transporter	2003	5000.1	226.8	314.9	1	0	0
NSCAC_0893		exported protein of unknown function	1963.4	1448.9	298.6	107.2	-1	0	0
NSCAC_1725		Cytochrome c, class I	1883.2	1031.1	418.2	331.3	-1	0	0
NSCAC_0278	trxA	thioredoxin 1	1882.2	1929.4	164	586.4	0	0.17	0.38
NSCAC_0895		exported protein of unknown function	1801.5	1940.8	253.7	138.8	0	0.01	0.06
NSCAC_0351	tufB	protein chain elongation factor EF-Tu, possible GTP-binding factor (duplicate of tufA)	1623	2538.3	589.6	51.2	0	0.23	0.46
NSCAC_1635		conserved exported protein of unknown function	1555.1	1246.2	179.5	20.1	-1	0	0
NSCAC_0894		protein of unknown function	1520.9	1337.4	229.4	69.4	-1	0	0
NSCAC_0970	folE	GTP cyclohydrolase 1	1517	767.6	54.6	50.8	-1	0	0
NSCAC_1723		conserved protein of unknown function	1507.8	449.3	134.9	164.4	-2	0	0

NSCAC_0469		Sigma-54 factor interaction domain-containing protein	1493.1	1647.2	213	305.2	0	0.24	0.47
NSCAC_0039		exported protein of unknown function	1482.3	4404.9	303.7	1861.6	1	0	0
NSCAC_1129		Putative Cytochrome P460	1469.4	957.9	284.2	177.6	-1	0	0
NSCAC_0230	ftsH	protease, ATP-dependent zinc-metallo	1462.1	1403	253.5	180.4	0	0	0
NSCAC_0688		exported protein of unknown function	1448.5	2957	110.6	288.1	1	0	0
NSCAC_1028		Aminoglycoside phosphotransferase (modular protein)	1423.1	1082.1	370.3	482.2	-1	0.04	0.12
NSCAC_0590		conserved protein of unknown function	1374.1	1398.9	180.2	189.1	0	0.04	0.13
NSCAC_0767		conserved membrane protein of unknown function	1360.4	1413.2	56	100.5	0	0.03	0.11
NSCAC_1368		conserved exported protein of unknown function	1331.2	1709.4	79.7	134.6	0	0.67	0.82
NSCAC_1496	rpmG	50S ribosomal protein L33	1320	1552.7	327	98.3	0	0.33	0.56
NSCAC_0888		conserved protein of unknown function	1290.2	1003.8	224.7	159.2	-1	0	0
NSCAC_1387		conserved protein of unknown function	1286.6	896	96.8	168.2	-1	0	0
NSCAC_0089	petC	Quinol-cytochrome c reductase, cytochrome c1 subunit	1266.9	1221.1	293.6	220.9	0	0	0.01
NSCAC_1126		conserved protein of unknown function, putative HaoB	1257.9	745.8	122.8	303.9	-1	0	0
NSCAC_1574	yrbE	toluene transporter subunit: membrane component of ABC superfamily	1248.5	1406.3	84.6	178.3	0	0.2	0.42
NSCAC_1306	sodB	superoxide dismutase, Fe	1228.6	833.3	352.5	286.6	-1	0	0
NSCAC_0373	secY	preprotein translocase membrane subunit	1159.2	1297.3	74.7	20.5	0	0.04	0.14
NSCAC_1565		conserved protein of unknown function	1139.2	703.7	208.6	160.4	-1	0	0
NSCAC_1724		Cytochrome c, class I	1133.4	425.3	94.2	107.4	-2	0	0
NSCAC_0115		conserved protein of unknown function	1132.7	1489.4	347.4	119.9	0	0.88	0.94
NSCAC_0369	rplR	50S ribosomal subunit protein L18	1100.7	680.9	306.1	436.7	-1	0	0
NSCAC_0339	tufB	protein chain elongation factor EF-Tu, possible GTP-binding factor (duplicate of tufA)	1078.6	1582.1	13.8	156.5	0	0.53	0.73
NSCAC_1713	ctaB	Protoheme IX farnesyltransferase	1075.4	443.9	247.8	42.3	-2	0	0
NSCAC_1149		Blue (Type1) copper domain-containing protein	1074.7	1221.1	316.1	348.1	0	0.22	0.44
NSCAC_0980	tal	Transaldolase	1061	729.6	395.9	273.2	-1	0	0
NSCAC_1100	csrA	pleiotropic regulatory protein for carbon source metabolism	1054.5	1246.4	187.6	165.2	0	0.36	0.6
NSCAC_0044		exported protein of unknown function	1048.4	2176.3	580.5	1636.8	1	0.36	0.59
NSCAC_0295		Ste24 endopeptidase	1028.3	595.9	54.9	32.5	-1	0	0
NSCAC_0783	coaX	Type III pantothenate kinase	1012.4	1062	230.7	58.7	0	0.05	0.15
NSCAC_0372	rplO	50S ribosomal subunit protein L15	970.9	892.1	187.7	217.7	-1	0	0.01

NSCAC_0141	hupA	HU, DNA-binding transcriptional regulator, alpha subunit	965.5	553.9	374.8	366.6	-1	0	0
NSCAC_1563	norB	Nitric oxide reductase	955.4	525.3	95.3	36.3	-1	0	0
NSCAC_0914	acpP	acyl carrier protein (ACP)	948.5	1456.9	66	31.3	0	0.32	0.55
NSCAC_0257		conserved protein of unknown function	943.5	990.8	123.9	21	0	0.05	0.17
NSCAC_0259		protein of unknown function	929.9	1332.3	246.8	75	0	0.53	0.73
NSCAC_0360	rplP	50S ribosomal protein L16	913.4	1289.7	296.7	512.3	0	0.81	0.9
NSCAC_0348	rpsL	30S ribosomal protein S12	904.4	1112.5	346.1	292.5	0	0.62	0.79
NSCAC_1157		protein of unknown function	896.7	187.9	213	21.3	-3	0	0
NSCAC_0091	petA	Quinol-cytochrome c reductase, iron-sulfur subunit	894.5	912.1	48.9	97.3	0	0.01	0.04
NSCAC_0367	rpsH	30S ribosomal subunit protein S8	891	1212.7	471.4	371.7	0	0.89	0.95
NSCAC_1768	atpD	F1 sector of membrane-bound ATP synthase, beta subunit	889.7	977.7	120	162.1	0	0.02	0.09
NSCAC_0440	tehB	putative AdoMet-dependent methyltransferase	888.7	894.3	98.3	206.1	0	0.1	0.26
NSCAC_0246	nuoL	NADH ubiquinone oxidoreductase subunit L	863.5	834.2	40.6	113.7	0	0.02	0.08
NSCAC_1627	hflC	Protein HflC	853.2	683.9	77.5	25.2	-1	0	0
NSCAC_0551		conserved protein of unknown function	844.4	509.9	412.5	209.7	-1	0	0
NSCAC_0904		conserved membrane protein of unknown function	834.7	729.5	47.2	197.1	-1	0.02	0.08
NSCAC_1672		Peptidoglycan-associated lipoprotein	827.8	940.2	361.2	232	0	0.37	0.6
NSCAC_1751	minE	cell division topological specificity factor	826.1	877.4	202.7	57.3	0	0.03	0.1
NSCAC_0350	fusA	protein chain elongation factor EF-G, GTP-binding	825.7	1325.5	120.7	122.1	0	0.04	0.13
NSCAC_0903		conserved membrane protein of unknown function	820.6	625	150.1	122.6	-1	0	0.01
NSCAC_0247	nuoM	NADH ubiquinone oxidoreductase subunit M	818.7	768.2	117.2	99.2	-1	0.02	0.09
NSCAC_0460		Heat shock protein Hsp20	809.2	1771.7	78.8	119.8	1	0	0
NSCAC_1767	atpC	F1 sector of membrane-bound ATP synthase, epsilon subunit	801.4	754.7	160.7	84.5	-1	0.01	0.03
NSCAC_1497		Fatty acid desaturase	799.2	559.1	132.3	77	-1	0	0
NSCAC_1156		Lipoprotein (fragment)	797	149.6	147.7	20.3	-3	0	0
NSCAC_0447		conserved protein of unknown function	791.6	593.2	127.4	159.3	-1	0	0
NSCAC_1720		protein of unknown function	790.9	270.4	344	34.5	-2	0	0
NSCAC_0356	rplB	50S ribosomal protein L2	785.7	1287.8	151.2	97.3	0	0.03	0.1
NSCAC_1391		Thiol:disulfide interchange protein	779.3	880.8	130.3	15.3	0	0.07	0.2
NSCAC_0933	pgsA	phosphatidylglycerophosphate synthetase	767.1	654.5	136.3	115.7	-1	0	0.02
NSCAC_1495	rpmB	50S ribosomal protein L28	752.9	713.6	466.7	384	0	0.24	0.46

NSCAC_1727	hisH	Imidazole glycerol phosphate synthase subunit HisH	749.4	778.6	119.2	255	0	0.23	0.46
NSCAC_0213	fba	Fructose-bisphosphate aldolase	742.4	776.3	105	117.5	0	0	0.01
NSCAC_0766		membrane protein of unknown function	730.5	684.5	118	67.4	-1	0.01	0.06
NSCAC_0359	rpsC	30S ribosomal protein S3	723.8	985.3	250.3	329.7	0	0.98	0.99
NSCAC_0083	pntB	pyridine nucleotide transhydrogenase, beta subunit	711.8	485.1	12.8	70	-1	0	0
NSCAC_1220	ccpA	Cytochrome c551 peroxidase	710.3	1585.7	136.6	495.1	1	0	0
NSCAC_1770	atpA	F1 sector of membrane-bound ATP synthase, alpha subunit	702.4	855.2	30.1	42.1	0	0.31	0.54
NSCAC_1773	atpE	F0 sector of membrane-bound ATP synthase, subunit c	702.1	822.6	157.4	233.5	0	0.49	0.71
NSCAC_0378	rplQ	50S ribosomal protein L17	697.7	945.8	111	45.6	0	0.93	0.97
NSCAC_0938	can2	Carbonic anhydrase 2	694.2	509.3	123	99.7	-1	0	0
NSCAC_0785		Sulfurtransferase	691.4	614.4	208.3	90.6	-1	0	0.01
NSCAC_1772	atpF	F0 sector of membrane-bound ATP synthase, subunit b	688.1	971.3	131	258	0	0.76	0.87
NSCAC_1212	ccmE	periplasmic heme chaperone	682.1	678.8	197.7	27	0	0.02	0.08
NSCAC_0344	rplJ	50S ribosomal subunit protein L10	676.3	907.6	157.6	146.2	0	0.92	0.97
NSCAC_0363	rplN	50S ribosomal protein L14	670.6	909.8	164.9	190.9	0	0.95	0.98
NSCAC_1267	rpe	D-ribulose-5-phosphate 3-epimerase	660.7	579.8	142.7	57.7	-1	0	0
NSCAC_1507		tRNA (5-methylaminomethyl-2-thiouridylate)-methyltransferase	659.1	1006.5	26.1	291	0	0.47	0.68
NSCAC_1626	hflK	modulator for HflB protease specific for phage lambda cII repressor	651.2	582.1	133.2	197.7	-1	0	0.02
NSCAC_1177	zwf	Glucose-6-phosphate 1-dehydrogenase	650.9	406.1	79.6	23.7	-1	0	0
NSCAC_1176	gnd	6-phosphogluconate dehydrogenase, decarboxylating	648.3	418.4	102.7	38.8	-1	0	0
NSCAC_0298	trxB	thioredoxin reductase 1	640.4	522.5	77.1	57.9	-1	0	0.01
NSCAC_0364	rplX	50S ribosomal subunit protein L24	636.3	1113	193.8	410.3	0	0.14	0.33
NSCAC_0992		Amidohydrolase	611.8	574.5	155.4	25.8	-1	0	0.02
NSCAC_0028	rpoD	RNA polymerase, sigma 70 (sigma D) factor	607.2	606.5	58.4	37.5	0	0	0.01
NSCAC_0090	petB	Quinol-cytochrome c reductase, cytochrome b subunit	607.2	620.2	17.9	57.1	0	0	0.02
NSCAC_0724	terE	Tellurium resistance protein TerE	601.9	439.1	231.6	230.8	-1	0	0.02
NSCAC_0829		conserved protein of unknown function	600.2	1783.8	208.2	394.4	1	0	0
NSCAC_1657		conserved exported protein of unknown function	599.9	389.1	179.1	74.8	-1	0	0
NSCAC_0543	aceE	Pyruvate dehydrogenase, decarboxylase component E1	593	471.3	79.3	46.1	-1	0	0
NSCAC_0240	nuoF	NADH-quinone oxidoreductase, F subunit	579.6	728.4	105.4	21.5	0	0.53	0.73
NSCAC_0705		DegP2 peptidase, Serine peptidase, MEROPS family S01B	578.3	221.9	74.4	10	-2	0	0

NSCAC_0238	nuoD	NADH-quinone oxidoreductase subunit D	572.4	721.5	54.9	69.5	0	0.59	0.77
NSCAC_1499		ABC transporter related protein	571.3	724.5	133.5	29.3	0	0.66	0.81
NSCAC_1765	glmS	L-glutamine:D-fructose-6-phosphate aminotransferase	566.1	415.2	29.7	23.1	-1	0	0
NSCAC_0343	rplA	50S ribosomal subunit protein L1	565.8	663.7	180.4	212.9	0	0.36	0.6
NSCAC_0353	rplC	50S ribosomal subunit protein L3	556.2	740.3	98.5	166.8	0	0.86	0.93
NSCAC_0264		conserved exported protein of unknown function	555.4	567.7	160.1	110.2	0	0.03	0.11
NSCAC_1118	glgC	glucose-1-phosphate adenylyltransferase	555	625.5	61.6	31.6	0	0.07	0.2
NSCAC_0210	gap	Glyceraldehyde-3-phosphate dehydrogenase	553.8	625	98.5	32.8	0	0.06	0.18
NSCAC_0950		conserved protein of unknown function	546.3	1027.6	79.4	186	0	0.02	0.07
NSCAC_1702		FAD-dependent pyridine nucleotide-disulfide oxidoreductase	546.3	382.1	92.9	20.9	-1	0	0
NSCAC_0293		Rhodanese-related sulfurtransferase	541.7	471.3	117.4	46.4	-1	0	0
NSCAC_1711	rpsT	30S ribosomal subunit protein S20	541.6	288.6	103.3	99.7	-1	0	0
NSCAC_0546		conserved membrane protein of unknown function	533.3	741.9	130.2	67.2	0	0.76	0.87
NSCAC_0886	rpsA	30S ribosomal subunit protein S1	533.1	702.6	108.5	308.4	0	0.82	0.9
NSCAC_1148		Amidohydrolase 2	529	858.6	96.8	184.2	0	0.09	0.25
NSCAC_1390	cycA	Cytochrome c class I	526.2	934.9	210.2	189.1	0	0.06	0.18
NSCAC_0905	terD	tellurium resistance protein	526.2	778.8	273.9	212.6	0	0.58	0.76
NSCAC_0248	nuoN	NADH ubiquinone oxidoreductase subunit N	522.9	609.1	60.1	124	0	0.35	0.58
NSCAC_0866		TonB-dependent heme/hemoglobin receptor family protein/TonB-dependent hemoglobin/transferrin/lactoferrin receptor family protein	520.7	338.5	40.3	75.4	-1	0	0
NSCAC_0376	rpsD	30S ribosomal protein S4	518.2	953.1	92.1	390	0	0.06	0.18
NSCAC_0365	rplE	50S ribosomal protein L5	513.1	644.1	121.2	230.1	0	0.58	0.76
NSCAC_0669	ispH	1-hydroxy-2-methyl-2-(E)-butenyl 4-diphosphate reductase, 4Fe-4S protein	510.6	555.5	109	117.8	0	0.05	0.16
NSCAC_0971	yahK	putative oxidoreductase, Zn-dependent and NAD(P)-binding	510.6	605.9	112	19	0	0.34	0.58
NSCAC_0541	gdh	Glutamate dehydrogenase	507.5	646	58.1	52.1	0	0.64	0.8
NSCAC_0498		conserved protein of unknown function	502.7	371.4	67.8	52.5	-1	0	0
NSCAC_1456	rpoZ	DNA-directed RNA polymerase subunit omega	500.5	833.9	95.2	144.4	0	0.04	0.12
NSCAC_0352	rpsJ	30S ribosomal protein S10	500.3	502.1	135.7	93.4	0	0.03	0.12
NSCAC_1256	ihfB	Integration host factor subunit beta	497.6	220.8	295.5	158.2	-2	0.01	0.05
NSCAC_1285		CDP-diacylglycerol/serine O-phosphatidyltransferase	496.3	599.5	96.7	108.5	0	0.5	0.71
NSCAC_1161	fdhA	Glutathione-independent formaldehyde dehydrogenase	492.2	627.9	109.4	40.9	0	0.65	0.8

NSCAC_0236	nuoB	NADH-quinone oxidoreductase subunit B 2	491.7	522.3	86.9	53	0	0.09	0.24
NSCAC_0175	rplM	50S ribosomal protein L13	490.5	812.6	188.5	167.2	0	0.16	0.37
NSCAC_1288	ilvI	Acetolactate synthase, large subunit	488.3	490.5	70	27.5	0	0	0
NSCAC_0241	nuoG	NADH-quinone oxidoreductase, chain G	488	509.2	19.3	54.7	0	0.01	0.03
NSCAC_0037		protein of unknown function	486.8	1606.6	140.1	734.9	1	0	0
NSCAC_0229	rrmJ	23S rRNA methyltransferase	486.1	1054.5	154.9	55.3	1	0	0
NSCAC_0926	clpP	ATP-dependent Clp protease proteolytic subunit (Endopeptidase Clp) (Caseinolytic protease) (Protease Ti) (Heat shock protein F21.5)	482	548.7	154	118.8	0	0.25	0.47
NSCAC_0244	nuoJ	NADH-ubiquinone/plastoquinone oxidoreductase chain J	482	601.6	61.8	26.6	0	0.53	0.73
NSCAC_0941	glk	Glucokinase	481.5	281.9	16.9	41.7	-1	0	0
NSCAC_1573	yrbD	toluene transporter subunit: membrane component of ABC superfamily	480.8	585.9	120.4	249.4	0	0.52	0.72
NSCAC_1728	hisA	Phosphoribosylformimino-5-aminoimidazole carboxamide ribotide isomerase	480	473.9	79.4	59.1	0	0.02	0.08
NSCAC_1327		O-antigen polymerase	478.5	287.7	61.9	2.6	-1	0	0
NSCAC_1210	ccmC	heme exporter subunit ; membrane component of ABC superfamily	473.3	432.5	133.3	50.7	-1	0.03	0.11
NSCAC_0586		conserved protein of unknown function	472.8	436.1	132	83.4	-1	0	0.01
NSCAC_1169		putative Multidrug resistance protein B	472.8	718	59.2	135.2	0	0.54	0.73
NSCAC_0564		conserved exported protein of unknown function	468.7	258.8	84.3	26.7	-1	0	0
NSCAC_0355	rplW	50S ribosomal subunit protein L23	468.6	820.6	154.9	403.5	0	0.23	0.46
NSCAC_0377	rpoA	RNA polymerase, alpha subunit	467.9	620.2	149	306	0	0.9	0.95
NSCAC_0242	nuoH	NADH:ubiquinone oxidoreductase, membrane subunit H	464.9	436.4	23.8	37.4	-1	0.01	0.04
NSCAC_0172		putative RNA-binding protein	464.7	848.7	121	89.1	0	0.02	0.07
NSCAC_1624		RNA-binding protein Hfq (modular protein)	463.6	369.5	89.9	76.1	-1	0	0
NSCAC_0031	groES	chaperone Hsp10, affects cell division	461.8	603.7	88.5	125.9	0	0.74	0.86
NSCAC_0668		Hopanoid biosynthesis associated radical SAM protein HpnH	460.3	577.1	58.5	94.5	0	0.43	0.66
NSCAC_1576		VacJ family lipoprotein	460.2	389.5	69	40.8	-1	0	0
NSCAC_0628		protein of unknown function	459.6	391.2	100.9	214.2	-1	0.02	0.08
NSCAC_0579	pnp	polynucleotide phosphorylase/polyadenylase	456.3	454.8	103.1	63.1	0	0	0.02
NSCAC_0174	rpsI	30S ribosomal protein S9	455.9	401.7	144.5	60	-1	0	0.01
NSCAC_0915	fabG	3-oxoacyl-[acyl-carrier-protein] reductase	454.9	415.6	99.5	69.3	-1	0	0
NSCAC_1769	atpG	F1 sector of membrane-bound ATP synthase, gamma subunit	453.2	641.2	89.7	145.6	0	0.71	0.84
NSCAC_1500		putative ABC transporter, permease protein	452.8	590.7	92.7	32.7	0	0.81	0.9

NSCAC_1286	ilvC	Ketol-acid reductoisomerase	444.8	486.3	32.8	47.5	0	0.03	0.1
NSCAC_1305		putative glutaredoxin-like protein (grxD-like)	444.3	662.3	106.9	62.7	0	0.7	0.83
NSCAC_1757	gcvT	aminomethyltransferase, tetrahydrofolate-dependent, subunit (T protein) of glycine cleavage complex	443.5	432.7	40.3	53.4	0	0.03	0.1
NSCAC_1755	hemC	hydroxymethylbilane synthase	440.4	516.7	37.1	14.2	0	0.13	0.32
NSCAC_0349	rpsG	30S ribosomal protein S7	439.6	839.6	71.7	215.2	0	0.01	0.04
NSCAC_1655	hemL	Glutamate-1-semialdehyde aminotransferase	437.4	382.9	98.1	29.2	-1	0	0
NSCAC_0357	rpsS	30S ribosomal protein S19	436.9	700.9	99	105.5	0	0.17	0.39
NSCAC_1530	metF	5,10-methylenetetrahydrofolate reductase	434.3	326.8	18.2	22.3	-1	0	0
NSCAC_0670	dxs	1-deoxyxylulose-5-phosphate synthase, thiamine-requiring, FAD-requiring	434	480.2	62.8	32.5	0	0.04	0.13
NSCAC_0575	infB	translation initiation factor IF-2	432.7	399.8	73.9	32.1	-1	0	0
NSCAC_1708	rpmA	50S ribosomal protein L27	427.3	852.8	59.8	129.1	1	0	0
NSCAC_1130	panD	aspartate 1-decarboxylase	427.2	275.1	78.2	53.1	-1	0	0
NSCAC_1729	hisF	imidazole glycerol phosphate synthase, subunit HisF	426.2	457	2.4	22.5	0	0.03	0.11
NSCAC_1714	ctaA	Heme A synthase	425	292.1	22.5	19	-1	0	0
NSCAC_1730	hisIE	Phosphoribosyl-AMP cyclohydrolase/phosphoribosyl-ATP pyrophosphatase	423.2	572.5	108.6	177.8	0	0.96	0.98
NSCAC_1707	rplU	50S ribosomal subunit protein L21	422.7	878.1	186.6	554.5	1	0.1	0.26
NSCAC_0328		conserved protein of unknown function	421	345.1	89.6	36.9	-1	0	0
NSCAC_0199	hemF	coproporphyrinogen III oxidase	418.2	476.2	33.9	121.4	0	0.36	0.59
NSCAC_0731	bfr	bacterioferritin, iron storage and detoxification protein	413.8	595.4	211.9	487.5	0	0.91	0.96
NSCAC_0484		HupE/UreJ protein	409.6	666.6	24.8	50.2	0	0.17	0.39
NSCAC_0777		conserved exported protein of unknown function	409.6	900.6	47.4	25.3	1	0	0
NSCAC_0889	rpoS	RNA polymerase, sigma S (sigma 38) factor	408.2	1861.9	147.1	1501.4	2	0	0.02
NSCAC_0366	rpsN	30S ribosomal protein S14	406.2	635.7	133.1	228.2	0	0.41	0.64
NSCAC_0383	cysD	Sulfate adenylyltransferase, small subunit	406.1	479.8	60.1	91.6	0	0.19	0.41
NSCAC_0725		Tellurite resistance TerB	405.4	459.9	47	204.9	0	0.28	0.52
NSCAC_0035		exported protein of unknown function	405.2	782.3	238.8	773	0	0.54	0.73
NSCAC_1750	minD	cell division inhibitor, a membrane ATPase,activates minC	403.6	485.2	128.5	79.1	0	0.42	0.65
NSCAC_0209	tktA	transketolase 1, thiamin-binding	403.5	410.6	18.4	35.6	0	0	0.01
NSCAC_1738	ybaL	Inner membrane protein YbaL	395.7	244.5	24.2	32.6	-1	0	0

NSCAC_0235	nuoA	NADH-quinone oxidoreductase subunit A	395.7	470.8	124.6	71.6	0	0.53	0.73
NSCAC_1717		conserved protein of unknown function	394.5	374.9	104.5	158.4	-1	0.13	0.32
NSCAC_1107	tsaB	tRNA threonylcarbamoyladenosine biosynthesis protein TsaB	389.9	505.9	83.2	88.2	0	0.81	0.9
NSCAC_0401	pyrH	uridylate kinase	385.3	360.9	85.3	95.6	-1	0	0.02
NSCAC_0726		Lipid A 3-O-deacylase-related protein (modular protein)	384.1	479	81.4	39.4	0	0.43	0.66
NSCAC_0930	treZ	Malto-oligosyltrehalose trehalohydrolase	382.7	333.9	25.9	24.9	-1	0	0
NSCAC_1474	suhB	inositol monophosphatase	382.5	429.1	75.4	9.5	0	0.09	0.23
NSCAC_0147	gspG	pseudopilin, cryptic, general secretion pathway	380.1	315.7	56.8	61.5	-1	0	0
NSCAC_0817	dksA	DNA-binding transcriptional regulator of rRNA transcription, DnaK suppressor protein	378.3	571.1	291.1	181.2	0	0.73	0.86
NSCAC_1553	argC	N-acetyl-gamma-glutamyl-phosphate reductase	377.1	450.2	59.5	45.2	0	0.16	0.37
NSCAC_0013	ctpA	Carboxy-terminal-processing protease	375.5	455.3	68.3	89	0	0.29	0.52
NSCAC_1610		conserved protein of unknown function	375.4	411.4	60.4	59.2	0	0.17	0.38
NSCAC_0354	rplD	50S ribosomal subunit protein L4	374.8	504.6	49.3	88.1	0	0.92	0.97
NSCAC_0329		conserved membrane protein of unknown function	374.2	350.4	61.7	113.1	-1	0.09	0.25
NSCAC_0243	nuoI	NADH-quinone oxidoreductase subunit I 2	373.1	465.1	9.2	45.3	0	0.54	0.73
NSCAC_0340	secE	Protein translocase subunit SecE	369.4	444.8	85.1	25.8	0	0.37	0.61
NSCAC_1544		conserved protein of unknown function	368.8	641.8	40.9	31.1	0	0.09	0.24
NSCAC_1014	aspC	Aspartate aminotransferase	363.8	234.8	48.5	20.1	-1	0	0
NSCAC_0052		putative tRNA-i(6)A37 modification enzyme (MiaB)	363.4	523.2	123.9	44.1	0	0.56	0.75
NSCAC_0594		conserved exported protein of unknown function	363.2	1090.9	26.9	39.3	1	0	0
NSCAC_1706	ispB	Trans-hexaprenyltranstransferase	361.8	248.3	95.9	26.7	-1	0	0
NSCAC_0542		protein of unknown function	359.5	283.3	92.1	44.4	-1	0	0
NSCAC_0423	glyA	serine hydroxymethyltransferase	354.2	442.8	57.7	98.2	0	0.45	0.67
NSCAC_1546	dsbD	Thiol:disulfide interchange protein DsbD	354.1	434.9	70.7	9.8	0	0.47	0.69
NSCAC_0125	sugE	multidrug efflux system protein	352.9	227.2	19.4	89.1	-1	0	0.01
NSCAC_0859	gmd	GDP-D-mannose dehydratase	352	438.2	21	26.1	0	0.51	0.71
NSCAC_1208	ccmA	heme exporter subunit ; ATP-binding component of ABC superfamily	350.4	490.4	79.2	14.1	0	0.7	0.83
NSCAC_1523		conserved membrane protein of unknown function	350	490.1	8.4	125.6	0	0.83	0.91
NSCAC_0667		Hopanoid biosynthesis associated membrane protein HpnM	349.5	298.5	11.3	102.7	-1	0	0.01
NSCAC_1189	fur	negative regulator of ferric iron uptake	348.8	433.9	110.4	131.5	0	0.59	0.77

NSCAC_1182		conserved protein of unknown function	346.7	349.9	38.3	35	0	0	0.01
NSCAC_1549	accB	acetyl CoA carboxylase, BCCP subunit	345.2	339.8	42.7	35.7	0	0.01	0.03
NSCAC_0975		conserved protein of unknown function	345.2	415.4	106.9	74.4	0	0.43	0.66
NSCAC_1006	himA	integration host factor (IHF), alpha subunit, DNA-binding protein, DNA replication	343.5	701	160.1	169.9	1	0.02	0.08
NSCAC_1307	argD	Acetylornithine aminotransferase	343	266.8	37.7	41.9	-1	0	0.01
NSCAC_0084	pntA	pyridine nucleotide transhydrogenase, alpha subunit	342.6	302.7	123.2	22.2	-1	0.01	0.04
NSCAC_0155	rpsF	30S ribosomal subunit protein S6	342.6	384	36.1	133	0	0.22	0.45
NSCAC_0990		conserved membrane protein of unknown function	341.4	162.6	84.5	25.6	-2	0	0
NSCAC_1550	accC	biotin carboxylase (A subunit of acetyl-CoA carboxylase)	341.3	447.1	37.2	29.5	0	0.81	0.9
NSCAC_0531	rplS	50S ribosomal protein L19	338.6	383.3	60.6	90.2	0	0.18	0.39
NSCAC_0375	rpsK	30S ribosomal protein S11	335.7	405.1	35.5	76.4	0	0.23	0.45
NSCAC_0939	apaH	diadenosine tetraphosphatase	335	334.5	50	63.7	0	0.08	0.21
NSCAC_0237	nuoC	NADH-quinone oxidoreductase subunit C	333.1	477.7	63	74.3	0	0.55	0.74
NSCAC_1649		MotA/TolQ/ExbB proton channel	333	225.3	24.9	16.1	-1	0	0
NSCAC_1710	proB	Glutamate 5-kinase	328.2	491.7	32.1	48.6	0	0.42	0.66
NSCAC_0502		ApbE family lipoprotein	327.9	254.8	38.6	17.8	-1	0	0
NSCAC_0406	yaeL	zinc metallopeptidase	326.1	475.2	34.3	31.7	0	0.49	0.71
NSCAC_0610		Outer membrane efflux protein	322.8	545.7	74.7	80.8	0	0.03	0.12
NSCAC_1213	ccmF	heme lyase, CcmF subunit	319.2	252.7	29.5	4.8	-1	0	0
NSCAC_1119	pgi	Glucose-6-phosphate isomerase	316.3	314.4	89.4	83.9	0	0.04	0.12
NSCAC_1275		conserved protein of unknown function	315	306.5	70.5	75.1	0	0.03	0.1
NSCAC_1774	atpB	F0 sector of membrane-bound ATP synthase, subunit a	314.9	484.8	98.5	77.1	0	0.43	0.66
NSCAC_0478	ureB	Urease beta subunit	312.7	1547.3	89.6	76.3	2	0	0
NSCAC_0942	pgl	6-phosphogluconolactonase	312.7	177.7	47.5	43.8	-1	0	0
NSCAC_1303	clpA	ATPase and specificity subunit of ClpA-ClpP ATP-dependent serine protease, chaperone activity	312.5	422.1	52.6	44.6	0	1	1
NSCAC_1211		Heme exporter protein CcmD	311.7	827	195.4	132.7	1	0.18	0.4
NSCAC_0666		Hopanoid biosynthesis associated RND transporter like protein HpnN	311	289.5	32.6	8	-1	0	0
NSCAC_0188		Methyltransferase FkbM family	308.5	325.6	113.3	111	0	0.16	0.36
NSCAC_0030	fxsA	inner membrane protein	307.9	283	111.8	53.1	-1	0.08	0.22
NSCAC_0913	fabF	3-oxoacyl-[acyl-carrier-protein] synthase II	307.4	356.5	39.8	32.9	0	0.25	0.47

NSCAC_0900		conserved exported protein of unknown function	307.3	333.3	142.3	188.4	0	0.37	0.61
NSCAC_0925	clpX	ATP-dependent Clp protease ATP-binding subunit	306.7	421.3	94.4	67.3	0	0.87	0.93
NSCAC_1555		Fe2-S2-type ferredoxin	306.2	307.1	79.3	73	0	0.05	0.15
NSCAC_0917	fabH	3-oxoacyl-[acyl-carrier-protein] synthase III	303.8	340.7	12.6	27.6	0	0.15	0.35
NSCAC_1771	atpH	F1 sector of membrane-bound ATP synthase, delta subunit	302	252.9	49	93.4	-1	0	0.01
NSCAC_0880	pheA	Chorismate mutase / Prephenate dehydratase	300.5	246.5	53.2	22	-1	0	0
NSCAC_0838		Ion transport protein	300	159.2	48.7	57.7	-1	0	0
NSCAC_1775		conserved membrane protein of unknown function	299.7	402.2	45.3	70.7	0	0.96	0.98
NSCAC_0341	nusG	transcription antitermination protein	298.3	511.2	34.9	83.7	0	0.06	0.18
NSCAC_0583	argG	Argininosuccinate synthase	296.7	371.6	99.5	21.2	0	0.65	0.81
NSCAC_0924	lon	DNA-binding ATP-dependent protease La	296.7	345.1	62.8	90.9	0	0.23	0.45
NSCAC_1435		conserved membrane protein of unknown function	295.1	404.8	31.9	22.2	0	0.99	0.99
NSCAC_0906	terA	Tellurium resistance protein TerA	295	398.7	57	34.1	0	0.94	0.98
NSCAC_0951		Outer membrane lipoprotein Slp	293.4	348	77.5	48	0	0.44	0.66
NSCAC_1012	thrS	threonyl-tRNA synthetase	291.8	346.4	79.7	22.1	0	0.33	0.57
NSCAC_1265	sufB	component of SufBCD complex	290.4	192.8	37	19.2	-1	0	0
NSCAC_0918	plsX	fatty acid/phospholipid synthesis protein	289.9	438.2	93.5	48.7	0	0.34	0.57
NSCAC_0074		conserved exported protein of unknown function	289.5	253.7	15.2	9.9	-1	0	0
NSCAC_1172		Secretion protein HlyD family	289	483.1	24.6	24.9	0	0.03	0.11
NSCAC_0919	rpmF	50S ribosomal subunit protein L32	288.9	852.2	29.9	62.6	1	0	0
NSCAC_0233	tpiA	Triosephosphate isomerase	286.4	283.3	148.2	37.6	0	0.15	0.34
NSCAC_0450		exported protein of unknown function	286	540.9	65.1	221.7	0	0.05	0.16
NSCAC_0405	dxr	1-deoxy-D-xylulose 5-phosphate reductoisomerase	285.8	241.3	49	18.7	-1	0	0.01
NSCAC_0327	secD	SecYEG protein translocase auxillary subunit	283.6	255.9	78	25.6	-1	0	0.01
NSCAC_1178		conserved protein of unknown function	283.4	260	73.5	35.5	-1	0.01	0.03
NSCAC_1634	infA	protein chain initiation factor IF-1	283.1	273.8	117.6	20.4	0	0.07	0.21
NSCAC_0578	rpsO	30S ribosomal subunit protein S15	282.4	400.8	81.4	104.1	0	0.72	0.85
NSCAC_0545	lpdA	Pyruvate dehydrogenase complex, dihydrolipoamide dehydrogenase component E3	281.7	377.9	37.2	27	0	0.93	0.97
NSCAC_0277		Phospholipid/glycerol acyltransferase	281.2	314.4	51.4	68.6	0	0.32	0.55
NSCAC_0347	rpoC	DNA-directed RNA polymerase beta' chain (Transcriptase beta' chain) (RNA polymerase beta' subunit)	280.9	375.3	55.1	77	0	0.9	0.96

NSCAC_1732	hitA	Protein HitA	280.9	400.6	114.9	28.3	0	0.76	0.87
NSCAC_0574	nusA	transcription termination/antitermination L factor	280.2	348.9	119.4	79	0	0.69	0.83
NSCAC_0963	trmU	tRNA (5-methylaminomethyl-2-thiouridylate)-methyltransferase	280.2	399	55.5	7.1	0	0.6	0.77
NSCAC_1011		protein chain initiation factor IF-3 (fragment)	280	344.2	112.7	45.1	0	0.69	0.83
NSCAC_0940	apaG	protein associated with Co2+ and Mg2+ efflux	279.4	263.9	80.8	5.4	-1	0.01	0.05
NSCAC_1325	rpoH	RNA polymerase, sigma 32 (sigma H) factor	278.1	609.1	115.4	181.7	1	0	0.02
NSCAC_1250	mucD	putative periplasmic serine endoprotease DegP-like	277.6	255.9	52.2	75.8	-1	0	0.02
NSCAC_1292	dapA	dihydrodipicolinate synthase	277	311.4	46.9	49.7	0	0.07	0.2
NSCAC_0004	fbp	Fructose-1,6-bisphosphatase class 1	275.8	292.1	44.4	53.4	0	0.11	0.27
NSCAC_0399	rpsB	30S ribosomal subunit protein S2	275.6	405.7	94.3	71.6	0	0.5	0.71
NSCAC_0693		conserved protein of unknown function	274.4	257.2	133	87.4	-1	0.08	0.23
NSCAC_0573	yhbC	conserved hypothetical protein	274.3	220.7	68.4	36.1	-1	0	0.02
NSCAC_0297		conserved protein of unknown function	273.3	188.4	40.5	39	-1	0	0
NSCAC_0400	tsf	protein chain elongation factor EF-Ts	273.3	316.4	62.3	124.3	0	0.31	0.54
NSCAC_0494		conserved exported protein of unknown function	272.8	207.9	69.2	17.4	-1	0	0
NSCAC_1502		conserved protein of unknown function	272.8	367.4	77.9	148.4	0	0.95	0.98
NSCAC_1192		conserved protein of unknown function	270.5	357.7	55.8	47.6	0	0.83	0.91
NSCAC_0758	acnA	Aconitate hydratase	270.5	277.2	32.6	17.4	0	0.03	0.12
NSCAC_1199		conserved protein of unknown function	269.1	369.9	59.9	112.9	0	0.92	0.97
NSCAC_1018		protein of unknown function	267.3	0	51.8	0	NA	NA	NA
NSCAC_1266		FeS assembly SUF system regulator	267.2	189.1	21.5	17.9	-1	0	0
NSCAC_0969	mdm C	O-methyltransferase MdmC	267	294	126.1	81.4	0	0.35	0.58
NSCAC_1469	pilA	Fimbrial protein	265.6	349.4	56	20.4	0	0.82	0.91
NSCAC_0544	aceF	Pyruvate dehydrogenase, dihydrolipoyltransacetylase component E2	265.2	352.6	17.1	45.3	0	0.82	0.9
NSCAC_1223		conserved protein of unknown function	263.7	395.4	45.7	25.7	0	0.33	0.56
NSCAC_0178	yadH	ABC transporter	263.5	235.1	31.3	77.4	-1	0.05	0.15
NSCAC_1287	ilvH	Acetolactate synthase, small subunit	261.8	314.3	66.8	28	0	0.32	0.56
NSCAC_0878	serC	phosphoserine aminotransferase	261.4	406.1	50.2	124.4	0	0.34	0.57
NSCAC_1064		conserved protein of unknown function	260.4	337.6	55.5	121.9	0	0.69	0.83
NSCAC_1049		Sel1 domain protein repeat-containing protein (fragment)	259.7	320	102.4	58.5	0	0.62	0.79

NSCAC_0757		conserved protein of unknown function	259.3	460.3	56.9	234.8	0	0.19	0.4
NSCAC_0221	pepP	proline aminopeptidase P II	259.1	207.8	67.7	29.9	-1	0	0
NSCAC_0212	pykA	pyruvate kinase II	257.8	335.4	56.4	26.1	0	0.76	0.87
NSCAC_0819		protein of unknown function	257.4	0	48.7	0	NA	NA	NA
NSCAC_1593	hemE	uroporphyrinogen decarboxylase	257	311.5	15.7	28.3	0	0.41	0.64
NSCAC_0616		conserved protein of unknown function	256.9	155.5	66	5.3	-1	0	0
NSCAC_1304	clpS	regulatory protein for ClpA substrate specificity	256.9	358.6	98.5	129.3	0	0.83	0.91
NSCAC_0374	rpsM	30S ribosomal protein S13	255.9	483.6	189.8	272.7	1	0.41	0.64
NSCAC_0704	gpmA	Phosphoglyceromutase	255.9	220.4	36.9	18.4	-1	0	0
NSCAC_1661	secF	Protein translocase subunit SecF	255.4	329.8	65.9	48.9	0	0.7	0.84
NSCAC_0784	perM	putative permease	254.8	428.9	26.8	32.5	0	0.11	0.28
NSCAC_0197	prkB	phosphoribulokinase	254.7	330.7	68.5	54.3	0	0.79	0.89
NSCAC_0427	rnhB	ribonuclease HII, degrades RNA of DNA-RNA hybrids	253.8	208.2	40.1	33.5	-1	0	0.01
NSCAC_1233	hupB	DNA-binding protein HU-beta	252.7	396.9	102	150.9	0	0.43	0.66
NSCAC_1651		conserved exported protein of unknown function	252.6	222.2	108.3	60.2	-1	0.02	0.09
NSCAC_0775		Sigma-54 factor interaction domain-containing protein	252.2	507.8	13.6	46.8	1	0	0.02
NSCAC_0927	tig	Trigger factor	252.1	315.8	61.2	86.1	0	0.57	0.75
NSCAC_0732		NnrS family protein	251.5	195.7	4.7	12.8	-1	0	0
NSCAC_1444		Toluene tolerance family protein	251.5	337.9	105	118	0	1	1
NSCAC_1486	dacC	D-alanyl-D-alanine carboxypeptidase DacC	251.3	246.4	57.1	23.9	0	0	0.02
NSCAC_0621		putative Spore coat protein CotH	251.2	454.8	9.3	110.1	0	0.08	0.22
NSCAC_0022	plsY	1-acyl glycerol-3-phosphate synthetase component	249.6	304.8	24.7	85.1	0	0.62	0.78
NSCAC_0033	yqjF	putative quinol oxidase subunit	249.3	220.5	91.4	74.2	-1	0.02	0.09
NSCAC_0480	ureE	Urease accessory protein UreE	249	2368.2	41.6	332.4	3	0	0
NSCAC_0614		conserved protein of unknown function	248.5	488	39.8	332.5	0	0.17	0.38
NSCAC_1742	rplY	50S ribosomal protein L25	245.9	392.2	91.1	138.3	0	0.34	0.57
NSCAC_0342	rplK	50S ribosomal protein L11	244.9	352.7	88.7	113.8	0	0.69	0.83
NSCAC_0547		Sel1 repeat protein (fragment)	244.8	476.7	72.1	33.8	1	0	0.01
NSCAC_1688		conserved exported protein of unknown function	242.9	350.9	46.8	40.8	0	0.5	0.71
NSCAC_1214	ccmG	periplasmic thioredoxin of cytochrome c-type biogenesis	242.9	271	48.1	41.8	0	0.14	0.34
NSCAC_1302		conserved protein of unknown function	238.1	265.2	92.6	59.1	0	0.31	0.54

NSCAC_0568		Serine--pyruvate transaminase	237.6	327.7	46.6	69.2	0	0.88	0.94
NSCAC_1068	trpB	tryptophan synthase beta chain	237.6	257.9	10.8	55.6	0	0.21	0.44
NSCAC_0887	ihfB	integration host factor (IHF), DNA-binding protein, beta subunit	236.9	227.5	122.4	91.5	0	0.19	0.41
NSCAC_0162		conserved membrane protein of unknown function	236.5	325.8	54.2	69.9	0	0.81	0.9
NSCAC_0883	aroA	3-phosphoshikimate 1-carboxyvinyltransferase	236.1	237.5	21.7	21.1	0	0.04	0.14
NSCAC_1065	folC	bifunctional folylpolyglutamate synthase and dihydrofolate synthase	235.5	290	21.3	24.9	0	0.49	0.7
NSCAC_1297		conserved exported protein of unknown function	235	510.9	46	99.3	1	0	0
NSCAC_0916	fabD	malonyl-CoA-[acyl-carrier-protein] transacylase	233.2	304.6	82.3	32.5	0	0.92	0.97
NSCAC_1412	ftsA	ATP-binding cell division protein involved in recruitment of FtsK to Z ring	232.9	386.7	7.9	61.9	0	0.15	0.35
NSCAC_0879	serA	D-3-phosphoglycerate dehydrogenase	232.8	396.8	52.6	91.1	0	0.08	0.21
NSCAC_1123	ndk	nucleoside diphosphate kinase (NDK) (NDP kinase) (Nucleoside-2-P kinase)	232.8	221.6	14.4	48.3	-1	0.01	0.03
NSCAC_0665	ispG	4-hydroxy-3-methylbut-2-en-1-yl diphosphate synthase	232.4	403.8	60.5	29.4	0	0.03	0.12
NSCAC_0739		RDD domain containing protein	232.2	260	55	22.9	0	0.21	0.43
NSCAC_1397		Protein-tyrosine phosphatase, low molecular weight	231.9	549.3	143.2	290.9	1	0.03	0.12
NSCAC_0696		conserved protein of unknown function	231.8	239.9	54.8	58.4	0	0.2	0.42
NSCAC_1160	acrB	multidrug efflux system protein	231.7	391.7	21.4	11.7	0	0.14	0.33
NSCAC_1271	trpG	bifunctional protein:[Includes: para-aminobenzoate synthase glutamine amidotransferase component II (ADC synthase); anthranilate synthase component II]	231.4	414.5	97.6	28	0	0.06	0.18
NSCAC_0177	uppP	Undecaprenyl-diphosphatase	230.1	247.4	61.7	41.1	0	0.24	0.47
NSCAC_1437		Mammalian cell entry related domain protein	230	270.1	34.5	42	0	0.21	0.43
NSCAC_0036		protein of unknown function	229.9	779.1	113.3	305.1	1	0	0
NSCAC_0526		Cytochrome c assembly protein	229.9	134.5	9.5	36.2	-1	0	0
NSCAC_1337		Asparagine synthetase	229.2	350.3	50.6	10.3	0	0.25	0.48
NSCAC_0211	pgk	phosphoglycerate kinase	229	269.2	24	40.1	0	0.18	0.39
NSCAC_0855		putative Glycosyl transferase group 1	228.9	289.1	46.9	84.2	0	0.58	0.76
NSCAC_0251	rbsK	Ribokinase	228.3	261.3	71.2	78.7	0	0.29	0.52
NSCAC_0935		conserved protein of unknown function	227.1	264.5	61.9	146.1	0	0.47	0.68
NSCAC_0596		conserved exported protein of unknown function	226.2	2026.5	53.5	114.5	3	0	0
NSCAC_1420	mraY	phospho-N-acetylmuramoyl-pentapeptide transferase	226.1	372.9	31.1	34.9	0	0.22	0.44

NSCAC_1097		Helix-turn-helix protein	225.5	171.8	53.3	38.7	-1	0	0.01
NSCAC_1215	ccmH	Cytochrome c-type biogenesis protein CcmH	223.7	324.8	57.4	53	0	0.6	0.77
NSCAC_0404		Phosphatidate cytidylyltransferase	223.5	230	40.3	38.7	0	0.09	0.25
NSCAC_1781	yidC	Membrane protein insertase YidC	223.3	252	74.3	28.1	0	0.25	0.48
NSCAC_0477	ureA	Urease gamma subunit	223.3	497.9	127.7	113.4	1	0.06	0.17
NSCAC_1537	ubiD	3-octaprenyl-4-hydroxybenzoate decarboxylase	223	156	47.2	21.1	-1	0	0
NSCAC_0379	ubiG	bifunctional 3-demethylubiquinone-9 3-methyltransferase and 2-octaprenyl-6-hydroxy phenol methylase	221.8	356.5	19.9	55.5	0	0.24	0.47
NSCAC_0207	dnaJ	chaperone Hsp40, co-chaperone with DnaK	221.6	332.6	75.1	20.8	0	0.45	0.67
NSCAC_1231	fabI	enoyl-[acyl-carrier-protein] reductase, NADH-dependent	221	175.9	36.6	11.8	-1	0	0
NSCAC_1217		protein of unknown function	220.9	501.3	57.6	309.6	1	0.03	0.11
NSCAC_0346	rpoB	DNA-directed RNA polymerase beta chain (Transcriptase beta chain) (RNA polymerase beta subunit)	220.8	307.5	3.7	35	0	0.86	0.93
NSCAC_0227	carB	carbamoyl-phosphate synthase, large subunit	220.7	211.4	10.2	5.6	-1	0	0.02
NSCAC_0752	cysE	serine acetyltransferase	220.5	107.8	42.1	30	-1	0	0
NSCAC_1643		protein of unknown function	220.3	157.5	101	23.8	-1	0.01	0.04
NSCAC_0206	dnaK	chaperone Hsp70 in DNA biosynthesis/cell division	219.5	409.4	32.6	128.2	0	0.04	0.13
NSCAC_0424	ribD	Riboflavin biosynthesis protein RibD [Includes: Diaminohydroxyphosphoribosylaminopyrimidine deaminase ; 5-amino-6-(5-phosphoribosylamino)uracil reductase]	219.1	301.1	21.9	62.2	0	0.94	0.98
NSCAC_0337	hslV	peptidase component of the HslUV protease	219	261.1	69.3	13.6	0	0.47	0.69
NSCAC_1261	nifU	NifU-like protein	218.5	158.1	84.1	41.4	-1	0	0.01
NSCAC_1554	erpA	Iron-sulfur cluster insertion protein ErpA 2	218.3	375.3	61.8	172.1	0	0.24	0.47
NSCAC_1477	sucC	succinyl-CoA ligase subunit beta	218.2	195	14.9	8.4	-1	0	0.01
NSCAC_0245	nuoK	NADH-quinone oxidoreductase subunit K	218.1	186	24.2	51.9	-1	0.01	0.04
NSCAC_1715		conserved protein of unknown function	217.3	174.6	28.9	30.7	-1	0	0
NSCAC_0964	hflD	High frequency lysogenization protein HflD homolog	216.8	250	96.5	25.9	0	0.5	0.71
NSCAC_1493		Uncharacterized 37.6 kDa protein in cld 5'region	216.6	282.4	86.9	6.2	0	0.93	0.97
NSCAC_0580	fabA	beta-hydroxydecanoyl thioester dehydrase	216.2	216.8	102.5	5.9	0	0.14	0.33
NSCAC_1491	gatA	aspartyl/glutamyl-tRNA(Asn/Gln) amidotransferase subunit A	216	290.2	12.1	30.7	0	0.95	0.98
NSCAC_0163	lpxD	UDP-3-O-acylglucosamine N-acyltransferase	215.1	317.7	25.6	65.4	0	0.63	0.79
NSCAC_0860	wcaI	putative glycosyl transferase	214.8	143.6	41.1	14.4	-1	0	0

NSCAC_0468		conserved protein of unknown function	213.8	428.2	47.4	186.4	1	0.04	0.14
NSCAC_0161		putative oxidoreductase, aryl-alcohol dehydrogenase like protein	213.4	310.8	17.6	23.5	0	0.54	0.74
NSCAC_0523		conserved protein of unknown function	213.2	184.6	49.1	45	-1	0.03	0.1
NSCAC_1528	metK	S-adenosylmethionine synthase	213	303	33.8	15.8	0	0.68	0.82
NSCAC_0475		Urea transporter	212.3	456.3	22.6	51.1	1	0	0
NSCAC_1647		TonB family C-terminal domain protein	212	333.3	71.1	27.6	0	0.27	0.5
NSCAC_1066	accD	acetyl-CoA carboxylase, beta (carboxyltranferase) subunit	211.5	244.5	23	19.1	0	0.24	0.47
NSCAC_1744	ispE	4-diphosphocytidyl-2-C-methylerythritol kinase	211.2	340.4	36.7	14.4	0	0.16	0.37
NSCAC_1206		GTP-binding protein HSR1-related protein	211	108	22.3	7.4	-1	0	0
NSCAC_1547		Alkyl hydroperoxide reductase/ Thiol specific antioxidant/ Mal allergen	210.7	321	32	75.4	0	0.38	0.62
NSCAC_1147	yhjE	putative transporter	210.5	201.1	50.4	47.1	-1	0.1	0.26
NSCAC_0734		conserved protein of unknown function	208.4	317.7	38.5	60.7	0	0.29	0.52
NSCAC_0921	purF	amidophosphoribosyltransferase	207.4	256.3	14.9	68.1	0	0.45	0.67
NSCAC_1005		Regulatory protein MerR	206.7	460.5	41	71.1	1	0	0
NSCAC_0595		conserved exported protein of unknown function	206.7	703.3	13.7	49.3	1	0	0
NSCAC_1086		putative [NiFe]-hydrogenase, large subunit HyfG	206.5	247.5	32.6	76.8	0	0.58	0.76
NSCAC_1716		putative Cytochrome oxidase biogenesis protein Surf1	206.3	138.4	38.8	3.8	-1	0	0
NSCAC_1152		conserved membrane protein of unknown function	206.2	238.7	11.6	28.3	0	0.27	0.51
NSCAC_0029		protein of unknown function	206.1	142.5	24.4	40.4	-1	0	0.01
NSCAC_0201	pyrD	Dihydroorotate dehydrogenase B (NAD(+)), catalytic subunit	206	196.8	57.7	32.3	-1	0.07	0.2
NSCAC_0525		protein of unknown function	205.9	218.7	38.7	25.1	0	0.05	0.16
NSCAC_1115	gcvP A	putative glycine dehydrogenase (decarboxylating) subunit 1	205.3	288.8	46	54	0	0.73	0.86
NSCAC_0501		conserved protein of unknown function	204.9	170.2	9	12.5	-1	0	0
NSCAC_0261		putative Lipoprotein	204	351.1	52.4	51.3	0	0.06	0.19
NSCAC_0239	nuoE	NADH-quinone oxidoreductase subunit E	203.9	295.9	63.5	33.2	0	0.84	0.92
NSCAC_0974	pyk	Pyruvate kinase	203.6	193.3	16.8	18	-1	0	0.01
NSCAC_1309	mpg1	Mannose-1-phosphate guanyltransferase	203.3	270.6	7.9	26.6	0	0.91	0.97
NSCAC_0265		exported protein of unknown function	201.7	357.1	53.5	99.6	0	0.06	0.18
NSCAC_1284	leuA	2-isopropylmalate synthase	201.6	170.1	45.2	18.1	-1	0	0
NSCAC_1351	ilvD	dihydroxy-acid dehydratase	201.5	177.2	36.9	12.8	-1	0	0

NSCAC_1639	guaB	IMP dehydrogenase	200.6	217.5	48.8	29.4	0	0.07	0.2
NSCAC_1418	ftsW	integral membrane protein involved in stabilizing FstZ ring during cell division	199.8	300.7	19.7	73.7	0	0.58	0.76
NSCAC_1650		Outer membrane transport energization protein ExbB	199.6	155.9	31.7	27.4	-1	0	0
NSCAC_1341		Sugar transferase, PEP-CTERM/EpsH1 system associated	199.6	198.3	12.9	19.3	0	0.03	0.1
NSCAC_1143		conserved exported protein of unknown function	199.4	251.5	182.4	234.8	0	0.92	0.97
NSCAC_0387	surE	broad specificity 5'(3')-nucleotidase and polyphosphatase	198.6	251.9	47.6	36.2	0	0.67	0.82
NSCAC_1343		Polysaccharide deactylase family protein, PEP-CTERM locus subfamily	198.5	227.3	17.3	46.4	0	0.14	0.33
NSCAC_0885	cmk	cytidylate kinase	197.2	236.1	49.5	36.4	0	0.5	0.71
NSCAC_1135		conserved membrane protein of unknown function	197.2	205.2	36.3	72.4	0	0.21	0.43
NSCAC_0762		putative Hopanoid biosynthesis associated glycosyl transferase protein HpnI	197	153.1	23.6	15.9	-1	0	0
NSCAC_1355		conserved protein of unknown function	196.7	838.7	30.5	33.2	2	0	0
NSCAC_1008	pheS	phenylalanine tRNA synthetase, alpha subunit	196.1	165.8	35.4	22.2	-1	0	0.02
NSCAC_1216		Cytochrome c-type biogenesis protein CcmI	195.8	218.4	57.5	15.7	0	0.16	0.37
NSCAC_1726	hisB	Imidazoleglycerol-phosphate dehydratase	195.3	252	80.5	82.5	0	0.78	0.89
NSCAC_1540		conserved protein of unknown function	194.1	226.7	21.7	46.4	0	0.2	0.42
NSCAC_1165		conserved membrane protein of unknown function	194.1	201.7	49.6	39.8	0	0.16	0.37
NSCAC_0226	carA	carbamoyl-phosphate synthase, small subunit	193.9	175.7	17.5	24.7	-1	0	0
NSCAC_0479	ureC	Urease alpha subunit	193.8	609.8	6.9	78.6	1	0	0
NSCAC_1298		OmpA/MotB domain protein	193.7	217.8	66.9	43.7	0	0.25	0.47
NSCAC_0402	frr	ribosome recycling factor	193.7	220.9	36.5	56.8	0	0.23	0.46
NSCAC_0626	frmB	S-formylglutathione hydrolase	193.3	191.5	12.7	29.2	0	0.02	0.07
NSCAC_0801	cysG	Siroheme synthase [Includes: Uroporphyrinogen-III C-methyltransferase ; Precorrin-2 dehydrogenase ; Sirohydrochlorin ferrochelatase]	192.6	244	53	8.6	0	0.71	0.84
NSCAC_1136	guaD	Guanine deaminase	191.7	178.2	59.7	41.1	-1	0.06	0.17
NSCAC_0723		conserved protein of unknown function	190.8	137.1	9.5	24	-1	0	0
NSCAC_0171	deaD	ATP-dependent RNA helicase DeaD	190.6	275.4	36.9	82.5	0	0.74	0.86
NSCAC_1226		putative Glycosyl transferase family 1	190.3	256.9	13.8	6.9	0	1	1
NSCAC_1009	rplT	50S ribosomal protein L20, also posttranslational autoregulator	190.3	272.7	50.3	29.1	0	0.59	0.77
NSCAC_1604	yajR	Inner membrane transport protein YajR	190.1	137.9	42.5	15.8	-1	0	0
NSCAC_1631	ybaB	conserved hypothetical protein	189.3	368.3	53.4	120.7	1	0.02	0.09

NSCAC_1268		Phosphoglycolate phosphatase	189.2	268.1	52.8	6.6	0	0.65	0.8
NSCAC_0772		Thiamine biosynthesis protein ThiS	188.8	259.6	82.6	56.6	0	1	1
NSCAC_1061		protein of unknown function	188.8	225.5	106.4	17.5	0	0.59	0.77
NSCAC_1209	ccmB	heme exporter subunit ; membrane component of ABC superfamily	188.2	251.4	4.6	53.8	0	0.95	0.98
NSCAC_0702	ycgN	conserved hypothetical protein	188.1	299.3	45.8	85.8	0	0.52	0.72
NSCAC_1411	ftsZ	GTP-binding tubulin-like cell division protein	187.5	338.1	45.6	64.9	0	0.14	0.34
NSCAC_1155		Amino acid peptide transporter	187.4	270.9	6.7	23.8	0	0.61	0.78
NSCAC_1171		Putative outer membrane chanel lipoprotein (modular protein)	187.3	380.3	48.4	33.3	1	0	0.02
NSCAC_1434		conserved protein of unknown function	187.2	238.8	50.8	58.2	0	0.62	0.78
NSCAC_1501		conserved protein of unknown function	186.5	287.3	56.8	122.4	0	0.5	0.71
NSCAC_0392	pdxJ	pyridoxine 5'-phosphate synthase	186	276.3	69.9	31	0	0.5	0.71
NSCAC_0720		conserved membrane protein of unknown function	186	218.6	10.1	34	0	0.29	0.52
NSCAC_1035		conserved membrane protein of unknown function	185.8	216.6	75.6	26.9	0	0.43	0.66
NSCAC_0695		putative Patatin-like phospholipase	185.4	125.6	66.9	34.2	-1	0	0.02
NSCAC_0446		Trehalase	185.4	238.3	38.1	17.2	0	0.76	0.87
NSCAC_1239		Nitrilase/cyanide hydratase and apolipoprotein N-acyltransferase	185.4	277.9	11.9	20.2	0	0.49	0.71
NSCAC_1681	yebC	conserved hypothetical protein	185.3	237	98	38.7	0	0.85	0.92
NSCAC_1407		conserved membrane protein of unknown function	185	221.6	51.9	11.2	0	0.47	0.68
NSCAC_1333		putative Polysaccharide biosynthesis protein	184.8	157.5	27.8	18.6	-1	0.01	0.04
NSCAC_0730	cmoA	tRNA (cmo5U34)-methyltransferase	184.7	179.1	131.6	33.2	0	0.23	0.46
NSCAC_1439		conserved protein of unknown function	184.4	230.8	53.1	64.7	0	0.62	0.78
NSCAC_0384	cysN	Sulfate adenylyltransferase, large subunit	184.2	228.7	26.6	47.9	0	0.47	0.68
NSCAC_1269	trpE	Anthranilate synthase component 1	183	170.1	11.6	12.9	-1	0.01	0.03
NSCAC_1145	qseB	Transcriptional regulatory protein QseB	182.8	193.6	35.8	53.9	0	0.26	0.49
NSCAC_1076	asd	Aspartate-semialdehyde dehydrogenase	182.5	118.1	81.8	15.3	-1	0	0
NSCAC_1260		FeS assembly SUF system protein SufT	182.4	200.4	37.5	32.4	0	0.15	0.35
NSCAC_0220		2-polyprenyl-6-methoxyphenol 4-hydroxylase	182	153.2	11.1	21.5	-1	0	0.01
NSCAC_0167		Type I secretion outer membrane protein, TolC family	182	580.5	27	55.5	1	0	0
NSCAC_0326		Preprotein translocase, YajC subunit	181.9	266.5	24	72.4	0	0.62	0.79
NSCAC_0059	trkH	potassium transporter	181.6	266	29.2	15.9	0	0.46	0.67
NSCAC_0703		conserved exported protein of unknown function	181.5	336.2	37.8	66.5	0	0.12	0.29

NSCAC_1350	yqjG	S-transferase	180.5	154.4	7.4	29.5	-1	0	0.01
NSCAC_1262	sufS	selenocysteine lyase, PLP-dependent	180.5	128.2	45.7	19.1	-1	0	0
NSCAC_0796	eno	enolase	180.1	179.3	39.3	23.8	0	0.02	0.09
NSCAC_0711		TonB-system energizer ExbB	179.9	217.1	18.6	18	0	0.34	0.58
NSCAC_1170		putative outer membrane chanel lipoprotein	179.7	324.4	20.2	7.9	0	0.01	0.03
NSCAC_0157	rplI	50S ribosomal subunit protein L9	179.1	287.3	102.7	154.8	0	0.5	0.71
NSCAC_0625	frmA	alcohol dehydrogenase class III/glutathione-dependent formaldehyde dehydrogenase	179.1	162.8	56.5	8.6	-1	0.01	0.04
NSCAC_0679		conserved membrane protein of unknown function	179	403.8	72	35.1	1	0	0.01
NSCAC_1529	ahcY	Adenosylhomocysteinase	178.8	233.4	20.7	16.9	0	0.74	0.86
NSCAC_0381		conserved membrane protein of unknown function	178.6	121.5	16.7	4.2	-1	0	0
NSCAC_1552	purH	fused IMP cyclohydrolase ; phosphoribosylaminoimidazolecarboxamide formyltransferase	178.4	259.6	41.4	23.6	0	0.64	0.8
NSCAC_1675	tolR	Protein TolR	178.2	223.6	74.8	33	0	0.77	0.88
NSCAC_1473	bipA	GTP-binding protein	178.2	182.7	25	29.7	0	0.02	0.08
NSCAC_1289	pyrC	Dihydroorotase	178	326.8	13.6	66.1	0	0.05	0.15
NSCAC_0984		putative FAD linked oxidase domain protein	178	274.4	60.6	69.4	0	0.41	0.64
NSCAC_0822		conserved protein of unknown function	177.3	145.7	111.7	72.1	-1	0.07	0.2
NSCAC_1264	sufC	component of SufBCD complex, ATP-binding component of ABC superfamily	177.2	140.7	30.6	50.2	-1	0	0.01
NSCAC_0776		ATP-binding region ATPase domain protein	177	435.9	24.4	64.4	1	0	0
NSCAC_1531	gloA	lactoylglutathione lyase	176.5	289	54.3	46.5	0	0.18	0.4
NSCAC_1243	hom	Homoserine dehydrogenase	176.3	220.3	20.1	24.3	0	0.58	0.76
NSCAC_1570	murA	UDP-N-acetylglucosamine 1-carboxyvinyltransferase (Enoylpyruvate transferase) (UDP-N-acetylglucosamine enolpyruvyl transferase) (EPT)	176.3	259.3	20.2	5.3	0	0.42	0.65
NSCAC_1203	ybbK	putative protease, membrane anchored	175.1	282.9	69.8	21.9	0	0.26	0.49
NSCAC_1159		Multidrug resistance protein	174.9	396.3	25.1	42.6	1	0	0
NSCAC_0589	cbbX	Protein CbbX	174.1	103	26.3	16.6	-1	0	0
NSCAC_0552	cysJ	Sulfite reductase [NADPH] flavoprotein, alpha subunit	173.4	110	21.3	6.7	-1	0	0
NSCAC_1464		conserved protein of unknown function	173.4	174.5	85.8	37.7	0	0.19	0.4
NSCAC_0680		6-phosphogluconate dehydrogenase NAD-binding protein	173	166.9	24.6	63.6	-1	0.04	0.12
NSCAC_1689		conserved protein of unknown function	171.6	264.2	101.4	150.2	0	0.63	0.79

NSCAC_0503		conserved membrane protein of unknown function	169.7	156.3	36.6	18.4	-1	0.04	0.13
NSCAC_1279		protein of unknown function	169	86.4	102.7	69.6	-1	0.09	0.24
NSCAC_1154		putative Secreted protein containing PEP-CTERM bacterial domain protein	168.9	405.8	11.9	64.4	1	0	0
NSCAC_0983		putative enzyme	168.9	401.8	64.9	139.9	1	0.01	0.05
NSCAC_1505	glnA	glutamine synthetase	168.9	269.5	52.3	17.7	0	0.22	0.44
NSCAC_0727	dfrA	putative dihydroflavonol-4-reductase	168.6	268.1	36.1	29.2	0	0.15	0.35
NSCAC_1568	hisD	Histidinol dehydrogenase	168.5	216.9	42.1	4.3	0	0.78	0.89
NSCAC_0358	rplV	50S ribosomal subunit protein L22	168.3	212	20	164.8	0	0.72	0.85
NSCAC_1664		Binding-protein-dependent transport systems inner membrane component	168.2	182.3	6.8	16.6	0	0.13	0.32
NSCAC_1137		Short-chain dehydrogenase/reductase SDR	168.1	184.6	69.4	32	0	0.3	0.54
NSCAC_0491	mip	Outer membrane protein MIP	168	184.8	35.1	126.7	0	0.36	0.6
NSCAC_0453		conserved protein of unknown function	165.4	179.2	10.1	24.6	0	0.18	0.39
NSCAC_1000		putative O-antigen acetylase	165.4	249.7	46.2	62.5	0	0.6	0.77
NSCAC_1591	aroC	Chorismate synthase	165.2	335.7	17.1	32.7	1	0.01	0.05
NSCAC_0771	thiG	thiamine biosynthesis protein, thiazole moiety	164.9	112	43.3	27.3	-1	0	0
NSCAC_0706		conserved protein of unknown function	164.4	214	11.3	5.6	0	0.76	0.87
NSCAC_0584	purB	adenylosuccinate lyase	164	243.8	53.6	23	0	0.45	0.67
NSCAC_1340		Exosortase 1	163.9	217.3	11.4	35.5	0	0.82	0.91
NSCAC_1571		BolA family protein	163.8	674.5	57.6	40.6	2	0	0
NSCAC_0553	cysI	Sulfite reductase [NADPH] hemoprotein, beta subunit	163.8	114.6	24.4	31.1	-1	0	0
NSCAC_0325	tgt	queuine tRNA-ribosyltransferase (tRNA-guanine transglycosylase) (Guanine insertion enzyme)	163.4	116.6	24.6	22.2	-1	0	0
NSCAC_1754		Uroporphyrinogen III synthase HEM4	163.3	294.4	36.7	47.6	0	0.04	0.14
NSCAC_0565		conserved membrane protein of unknown function	161.1	131.1	27.8	17.5	-1	0	0.02
NSCAC_0452	icd	Isocitrate dehydrogenase	160.7	195.6	20.4	26.7	0	0.51	0.71
NSCAC_1436		ABC transporter related protein	160.5	199	41.4	25.4	0	0.66	0.81
NSCAC_1737	yhdG	Uncharacterized amino acid permease YhdG	160.4	205.8	13.7	23.8	0	0.72	0.85
NSCAC_1339		Sugar transferase, PEP-CTERM/EpsH1 system associated	160	200.6	61.2	53.7	0	0.71	0.85
NSCAC_1521	lolC	Lipoprotein-releasing system transmembrane protein LolC	159.5	128.7	56.7	17	-1	0	0.02
NSCAC_1484	lipB	lipoyl-protein ligase	159.4	240.4	21	35.9	0	0.46	0.68
NSCAC_0075		exported protein of unknown function	159.1	246.1	30.4	61.5	0	0.38	0.61

NSCAC_0608	cusA	copper/silver efflux system, membrane component	159	211.7	26.3	13.8	0	0.96	0.98
NSCAC_0270	yqgE	hypothetical protein	158.9	183.4	42.8	26.1	0	0.25	0.48
NSCAC_1075		NADH:flavin oxidoreductase	158.9	136.9	35.4	14.6	-1	0	0.01
NSCAC_1504		conserved protein of unknown function	158.8	163.9	75.7	24.3	0	0.18	0.4
NSCAC_1508		putative enzyme	158.1	175	43.8	35.9	0	0.22	0.44
NSCAC_0314	yciI	putative enzyme	157.6	478	44.1	64.4	1	0	0
NSCAC_1311		protein of unknown function	156.6	88.2	47.9	21.3	-1	0	0
NSCAC_1492		putative UDP-glucose/GDP-mannose dehydrogenase	156.4	279.6	52.5	77.5	0	0.09	0.23
NSCAC_1224		protein of unknown function	156.4	223.7	43.1	56.6	0	0.75	0.87
NSCAC_0232	glmM	phosphoglucosamine mutase	156	200.5	31.9	17.1	0	0.7	0.83
NSCAC_1766	glmU	fused N-acetyl glucosamine-1-phosphate uridyltransferase ; glucosamine-1-phosphate acetyl transferase	155.8	169.8	28.4	69.6	0	0.21	0.43
NSCAC_1776	parB	putative chromosome-partitioning protein ParB	155.8	326.4	33.4	9.2	1	0	0
NSCAC_0256		putative Acyltransferase 3	155.4	83.8	10.7	7.1	-1	0	0
NSCAC_0996		Peptidase S9 prolyl oligopeptidase active site domain protein	155.1	160.3	17.4	14.9	0	0.02	0.08
NSCAC_0781		NAD(P)H dehydrogenase (quinone)	155	284.7	58.7	27.8	0	0.19	0.41
NSCAC_0537	ppiB	peptidyl-prolyl cis-trans isomerase precursor (PPIase) (Rotamase)	154.9	320.1	103.2	139.9	1	0.09	0.24
NSCAC_1752		HemY domain protein	154.8	269	39.7	89	0	0.12	0.29
NSCAC_0094	nadA	Quinolinate synthase A	154.7	170.8	14	20.8	0	0.18	0.4
NSCAC_0304	cysQ	3'(2'),5'-bisphosphate nucleotidase CysQ	154.1	187.2	33.7	13.1	0	0.41	0.64
NSCAC_0821		conserved protein of unknown function	153.6	169.6	70.7	35.8	0	0.42	0.65
NSCAC_0127		putative pterin-4-alpha-carbinolamine dehydratase	153.4	316.6	119.6	141.3	1	0.48	0.69
NSCAC_0664	gloB	Hydroxyacylglutathione hydrolase	153.2	159.5	28.1	40.5	0	0.06	0.18
NSCAC_0252	gcvPB	putative glycine dehydrogenase (decarboxylating) subunit 2	153.2	198	56.1	17.3	0	0.9	0.96
NSCAC_0877	gyrA	DNA gyrase (type II topoisomerase), subunit A	152.9	196.1	45.4	9.4	0	0.78	0.89
NSCAC_0728		Endonuclease/exonuclease/phosphatase	152.6	356.6	23.2	28.2	1	0	0
NSCAC_0371	rpmD	50S ribosomal subunit protein L30	152.3	237.7	85.6	83.5	0	0.54	0.73
NSCAC_0763		Fe-S protein, radical SAM family	152.2	229.9	26.9	51.9	0	0.43	0.66
NSCAC_0276		D-glycero-beta-D-manno-heptose-1,7-bisphosphate 7-phosphatase	152.1	211.8	50.9	49	0	0.94	0.97
NSCAC_0048	lgt	phosphatidylglycerol-prolipoprotein diacylglyceryl transferase	152.1	140.9	17.2	45.9	-1	0.08	0.21
NSCAC_1743	prs	ribose-phosphate pyrophosphokinase (Phosphoribosyl pyrophosphate synthetase)	151.9	281.6	21.9	70.1	0	0.06	0.18

NSCAC_1196	purM	phosphoribosylaminoimidazole synthetase	151.5	160.7	12.7	12.4	0	0.08	0.23
NSCAC_0315		putative intracellular septation protein A	151.3	193	37.8	8	0	0.69	0.83
NSCAC_1248	lepA	GTP-binding protein	151.1	149.3	8.8	1.8	0	0.03	0.1
NSCAC_1381		protein of unknown function	151.1	224.7	31.7	21	0	0.41	0.64
NSCAC_0778		Ferredoxin 1	150.6	94	19.9	34.5	-1	0	0
NSCAC_0391	recO	DNA repair protein RecO	150.5	183.9	23.3	35.2	0	0.41	0.64
NSCAC_0719	terZ	Tellurium resistance protein TerZ	150.3	186.3	35.4	86.2	0	0.6	0.77
NSCAC_0449		conserved protein of unknown function	150.2	289.5	52.8	36	1	0.01	0.06
NSCAC_1122	yfgB	putative Fe-S containing enzyme	149.8	86.5	11.2	21.3	-1	0	0
NSCAC_0701		conserved protein of unknown function	149.6	279.9	46.4	114.9	0	0.09	0.24
NSCAC_1270	fsr	fosmidomycin efflux system, member of the major facilitator superfamily	149.4	262.9	16.2	11.2	0	0.03	0.1
NSCAC_0846		Binding-protein-dependent transport system protein	149	124	20.9	3.4	-1	0	0
NSCAC_1024	ppa	Inorganic pyrophosphatase	148.4	205.8	35.3	18.3	0	0.79	0.89
NSCAC_1648	exbD	Biopolymer transport protein exbD2	148.1	157.6	103.9	76.8	0	0.48	0.69
NSCAC_0419	hprA	Glycerate dehydrogenase	147.7	182.4	21.8	24.3	0	0.55	0.74
NSCAC_1685	copA	Copper-exporting P-type ATPase A	147.2	95.7	25.6	12.1	-1	0	0
NSCAC_1703		Uncharacterized transporter HI_0895	146.6	183.7	12.2	16.2	0	0.5	0.71
NSCAC_0753	iscS	Cysteine desulfurase	146.2	68.6	35.6	15.2	-1	0	0
NSCAC_0981		conserved membrane protein of unknown function	146.2	88	21.7	32.8	-1	0	0
NSCAC_1153		Methyltransferase domain family	146.1	173.8	36.8	49.5	0	0.4	0.64
NSCAC_0619	metA	Homoserine O-succinyltransferase	146	199.1	17.8	17.9	0	0.94	0.97
NSCAC_1244	alaC	Aspartate aminotransferase	146	175.5	8	21.3	0	0.41	0.64
NSCAC_0760	traL	Protein TraL	145.9	274.1	40.2	95	0	0.05	0.16
NSCAC_1548	aroQ	3-dehydroquinate dehydratase	145.7	256.2	82.5	63.9	0	0.25	0.48
NSCAC_1779	gidA	glucose-inhibited cell-division protein	145.3	232.5	13.8	33.5	0	0.27	0.5
NSCAC_0397		thiamin-monophosphate kinase (modular protein)	144.5	174.3	12.9	27.2	0	0.45	0.67
NSCAC_0815	acs	acetyl-CoA synthetase	144	199.1	30.4	30.9	0	0.81	0.9
NSCAC_0707		exported protein of unknown function	143.3	186.5	29.5	29.4	0	0.8	0.9
NSCAC_0421		conserved protein of unknown function	142.8	106.1	17.4	2.5	-1	0	0
NSCAC_1105		Abortive infection bacteriophage resistance protein	142.4	111.8	30.1	38.8	-1	0.02	0.09
NSCAC_0332	ubiB	2-octaprenylphenol hydroxylase	141.7	167.5	10	19.3	0	0.38	0.61

NSCAC_0330	mpl	UDP-N-acetylmuramate:L-alanyl-gamma-D-glutamyl-me so-diaminopimelate ligase	141.6	110.6	20	23.5	-1	0	0.01
NSCAC_1753		conserved protein of unknown function	141.4	235.4	34.5	138.8	0	0.4	0.64
NSCAC_1483	lipA	lipoate synthase	141	221.4	22.2	7.5	0	0.27	0.5
NSCAC_1067	trpA	Tryptophan synthase alpha chain	141	167	48.1	55.5	0	0.55	0.74
NSCAC_1296	nrdB	ribonucleoside-diphosphate reductase, beta subunit	140.4	160.3	53	48.1	0	0.45	0.67
NSCAC_0858		Glycosyl transferase, group 1	140.3	304.6	31.9	52.2	1	0	0
NSCAC_0225	hemB	Delta-aminolevulinic acid dehydratase	140.3	100.1	15.7	9.3	-1	0	0
NSCAC_1102	alaS	alanyl-tRNA synthetase	140.1	202.6	37.7	16.9	0	0.55	0.74
NSCAC_1746		Tetratricopeptide TPR_2 repeat protein	139.9	321	43.3	146.6	1	0.01	0.04
NSCAC_0773		conserved membrane protein of unknown function	139.9	207.6	21.2	4.7	0	0.61	0.78
NSCAC_1471		ATP-binding region ATPase domain protein (modular protein)	139.3	157.4	2.6	21.9	0	0.25	0.48
NSCAC_1522	lolD	outer membrane-specific lipoprotein transporter subunit ; ATP-binding component of ABC superfamily	138.8	132.5	25.3	16.1	-1	0.06	0.18
NSCAC_1663		Extracellular solute-binding protein family 5	138.7	169.8	16.4	10.8	0	0.42	0.65
NSCAC_1113		Peptidase M23	137.7	223.4	35.5	57.1	0	0.22	0.44
NSCAC_0331		putative aromatic acid decarboxylase	136.6	212.9	33.3	37.6	0	0.32	0.55
NSCAC_0993		conserved protein of unknown function	136.6	210.4	15.3	7.4	0	0.27	0.51
NSCAC_0995		conserved exported protein of unknown function	136.3	282.7	38.7	26.6	1	0	0.02
NSCAC_0937		conserved protein of unknown function	135.8	133.2	30.4	38.4	0	0.04	0.13
NSCAC_1004	metZ	O-succinylhomoserine sulfhydrylase	135.8	152.5	39.1	6.6	0	0.29	0.52
NSCAC_1733	tatA	TatABCE protein translocation system subunit	135.6	244.4	22.1	80.1	0	0.17	0.39
NSCAC_0673	metE	5-methyltetrahydropteroyltriglutamate-homocystein e S-methyltransferase	135.5	195.3	13.3	17.1	0	0.75	0.86
NSCAC_1646		TPR repeat-containing protein	135.5	142.7	27.6	53	0	0.15	0.35
NSCAC_0536	lpxH	UDP-2,3-diacylglucosamine pyrophosphatase	135.5	155.3	19.2	18.1	0	0.26	0.48
NSCAC_0142		conserved protein of unknown function	135.5	196.4	36.9	2.3	0	0.55	0.74
NSCAC_0677	ydaO	putative C32 tRNA thiolase	135.2	203.2	59.5	11.6	0	0.47	0.68
NSCAC_0070	aroB	3-dehydroquinate synthase	135.2	156.8	33	12.6	0	0.36	0.59
NSCAC_0336	hslU	molecular chaperone and ATPase component of HslUV protease	134.4	209	61.5	38.8	0	0.44	0.66
NSCAC_1406	argJ	Arginine biosynthesis bifunctional protein ArgJ	134.1	139.6	23.2	11.5	0	0.14	0.33
NSCAC_0009		Sporulation domain protein	133.6	135.1	91.7	50.6	0	0.33	0.56

NSCAC_1575	yrbF	toluene transporter subunit: ATP-binding component of ABC superfamily	133.6	285.9	39.2	24.6	1	0	0
NSCAC_1326		putative Trypsin	132.7	145.1	20.7	11.1	0	0.09	0.25
NSCAC_0839	mrp	antiporter inner membrane protein	132.3	115.2	24.2	7.6	-1	0	0.01
NSCAC_1628	hisZ	ATP phosphoribosyltransferase regulatory subunit	132.1	146.2	15.9	24.5	0	0.12	0.3
NSCAC_1038	htpG	molecular chaperone HSP90 family	132.1	226.2	15	26.2	0	0.07	0.2
NSCAC_0840		Protein-tyrosine-phosphatase	131.8	186.9	58.6	30.9	0	0.69	0.83
NSCAC_0743	can1	Carbonic anhydrase 1	131.6	157.3	47.6	18	0	0.57	0.76
NSCAC_1295	nrdA	ribonucleoside diphosphate reductase, alpha subunit	131.6	146.5	29.6	21.9	0	0.19	0.4
NSCAC_0219	visC	putative oxidoreductase with FAD/NAD(P)-binding domain	131.4	121.2	15.3	19.7	-1	0	0.02
NSCAC_0957		putative Cell division protein ZipA	130.7	275.6	11.9	14.3	1	0	0
NSCAC_1051	ksgA	S-adenosylmethionine-6-N',N'-adenosyl (rRNA) dimethyltransferase	130.3	131.2	41.7	19.1	0	0.17	0.38
NSCAC_1665		Binding-protein-dependent transport systems inner membrane component	130.2	147.2	11	29.1	0	0.31	0.55
NSCAC_1625	hflX	putative GTPase	129.7	205.3	23.4	34.1	0	0.2	0.42
NSCAC_0140	nsrR	DNA-binding transcriptional regulator	129.4	177	40.9	44.3	0	0.92	0.97
NSCAC_0455		conserved protein of unknown function	129.4	150.9	24.1	21.1	0	0.39	0.63
NSCAC_0944		glucuronosyltransferase	129.3	127.8	1.6	18.1	0	0.03	0.1
NSCAC_0863	wcaJ	putative UDP-sugar lipid carrier transferase	129.2	227.7	13.5	18.5	0	0.05	0.15
NSCAC_1404	khk	Ketohexokinase	128.8	157.3	21.2	17.9	0	0.52	0.72
NSCAC_0609		Efflux transporter, RND family, MFP subunit	128.6	196	38.9	55.6	0	0.45	0.67
NSCAC_1740	ychF	putative GTP-binding protein	128.5	197.2	19.1	51.4	0	0.58	0.76
NSCAC_1342		FemAB-related protein, PEP-CTERM system-associated	127.7	173.9	45.2	63.4	0	0.95	0.98
NSCAC_0222		conserved protein of unknown function	127.6	215.3	44.3	110.4	0	0.33	0.56
NSCAC_0410	lpxA	UDP-N-acetylglucosamine acetyltransferase	127.4	286.5	29.7	44.2	1	0.01	0.03
NSCAC_1026		conserved protein of unknown function	127.4	253.9	32.7	31.5	1	0.01	0.03
NSCAC_0755	ada	O6-methylguanine-DNA methyltransferase	127.1	221.7	53.4	55.7	0	0.16	0.36
NSCAC_0106	xerC	site-specific tyrosine recombinase	126.6	181.7	14.3	13.1	0	0.72	0.85
NSCAC_1735	tatC	TatABCE protein translocation system subunit	126.5	251.1	17.3	39.8	1	0.03	0.1
NSCAC_1324	ftsY	cell division protein	126.4	132.1	57.2	48.7	0	0.25	0.48
NSCAC_0714		Multidrug transporter	126.1	115.4	18.7	38.5	-1	0.09	0.24
NSCAC_0794	pyrG	CTP synthetase	126	171.6	21.6	21.2	0	0.96	0.98
NSCAC_0736	pepA	aminopeptidase A, a cyteinylglycinase	126	244.3	45.4	47.2	1	0.02	0.08

NSCAC_1680	ruvC	component of RuvABC resolvasome, endonuclease	125.8	111.9	46.3	16.9	-1	0.03	0.1
NSCAC_0195	thiC	hydroxymethylpyrimidine moiety synthesis in thiamin biosynthesis	125.6	129.3	33	23	0	0.15	0.35
NSCAC_0733		UspA domain protein	125.4	180.3	17.6	40.5	0	0.67	0.82
NSCAC_1218	coq	2-nonaprenyl-3-methyl-6-methoxy-1,4-benzoquinol hydroxylase	125.4	270.6	50.7	67.2	1	0.05	0.16
NSCAC_1632	recR	gap repair protein	124.9	133	27.8	33.4	0	0.16	0.37
NSCAC_0396	ribE	riboflavin synthase beta chain	124.7	231.2	48.6	32.8	1	0.06	0.19
NSCAC_0470		exported protein of unknown function	123.4	177.4	69.6	148.8	0	0.87	0.93
NSCAC_1020		protein of unknown function	123.1	189.4	35.2	60.4	0	0.56	0.75
NSCAC_1282		Ribosomal-protein-alanine acetyltransferase	122.9	125	24.2	14	0	0.04	0.14
NSCAC_0560		Hopanoid-associated phosphorylase	122.9	198.8	11.8	39.8	0	0.26	0.49
NSCAC_0529	rimM	16S rRNA processing protein	122.9	158.4	79.7	36.2	0	0.98	0.99
NSCAC_1232		PpiC-type peptidyl-prolyl cis-trans isomerase	122.9	182.8	4.9	91.9	0	0.73	0.86
NSCAC_0597		conserved membrane protein of unknown function	122.7	144.2	35.1	21.6	0	0.41	0.64
NSCAC_0864		Inositol monophosphatase	122.4	125.6	34.7	10.9	0	0.1	0.25
NSCAC_0408		Outer membrane chaperone Skp (OmpH)	122.4	269	61.7	147.5	1	0.08	0.22
NSCAC_0196	purD	phosphoribosylglycinamide synthetase phosphoribosylamine-glycine ligase	122.1	164.2	21.9	3.6	0	0.99	0.99
NSCAC_0234		Preprotein translocase, SecG subunit	122	196.5	68.2	47.8	0	0.43	0.66
NSCAC_1421	murF	UDP-N-acetylmuramoyl-tripeptide:D-alanyl-D-alanin e ligase	121.9	146.2	3	12.3	0	0.36	0.6
NSCAC_0922		Colicin V production protein	121.3	167.1	41.4	17.5	0	0.95	0.98
NSCAC_1181		protein of unknown function	121	121	31.4	24.1	0	0.1	0.26
NSCAC_0528	rpsP	30S ribosomal protein S16	121	186.3	71.7	19.9	0	0.49	0.7
NSCAC_1398	rne	Ribonuclease E	121	217.5	30.6	81	0	0.11	0.27
NSCAC_0561	shc	Squalene--hopene cyclase	120.9	137.1	44.7	11.1	0	0.33	0.56
NSCAC_0959	queF	NADPH-dependent 7-cyano-7-deazaguanine reductase	120.8	107.9	35.2	36.9	-1	0.12	0.29
NSCAC_1673	tolB	periplasmic protein	120.7	164.5	33.8	43.5	0	0.93	0.97
NSCAC_0026		conserved protein of unknown function	119.9	136.9	49.2	86.1	0	0.54	0.73
NSCAC_1677	ybgC	acyl-CoA thioesterase	119.7	176.4	44.8	35	0	0.72	0.85
NSCAC_1101	lysC	Aspartate kinase	119.6	210.7	50.8	27.5	0	0.1	0.26
NSCAC_0151		exported protein of unknown function	119.5	394.4	16.2	81.3	1	0	0
NSCAC_1164	def	Peptide deformylase 1	119.3	117.5	40.6	6.3	0	0.12	0.29

NSCAC_1545	cutA	Divalent-cation tolerance protein CutA	119.1	138.3	76.1	60.1	0	0.49	0.71
NSCAC_0857		protein of unknown function	118.5	142.8	33	64.1	0	0.5	0.71
NSCAC_1335		Glycosyl transferase, group 1	118.5	206.5	9.4	38.4	0	0.16	0.36
NSCAC_1185	smpB	trans-translation protein	118.4	110.7	7.7	16.9	-1	0.03	0.1
NSCAC_1402	pfp	Phosphofructokinase	118.3	127.7	21.2	34.7	0	0.15	0.35
NSCAC_0019		conserved protein of unknown function	118.3	167.1	9	36	0	0.8	0.9
NSCAC_0599		Cold-shock protein DNA-binding protein (fragment)	118.3	196	96.8	108.4	0	0.65	0.8
NSCAC_0324	pyrF	orotidine-5'-phosphate decarboxylase	118.1	92	39.6	12.5	-1	0.01	0.06
NSCAC_0456		NADPH-dependent FMN reductase	118.1	167.2	31.4	32.4	0	0.82	0.9
NSCAC_0361	rpmC	50S ribosomal subunit protein L29	117.2	131.3	83.4	116	0	0.6	0.77
NSCAC_1686		Heavy metal transport/detoxification protein	117.2	150.8	35.5	93.1	0	0.81	0.9
NSCAC_1348		PEP-CTERM system TPR-repeat lipoprotein	116.9	179.1	31.7	79	0	0.53	0.73
NSCAC_1577	kdsD	D-arabinose 5-phosphate isomerase	116.8	120.1	4.9	8.5	0	0.08	0.23
NSCAC_0021	folB	bifunctioal dihydroneopterin aldolase ; dihydroneopterin triphosphate 2'-epimerase	116.5	110.2	65.7	43.8	0	0.24	0.46
NSCAC_1780	trmE	GTPase	116.4	188.4	36.7	16.3	0	0.2	0.42
NSCAC_1299		conserved membrane protein of unknown function	116	140.4	33.1	32.1	0	0.6	0.77
NSCAC_0577	truB	tRNA pseudouridine synthase	115.5	202.5	38.7	23.5	0	0.06	0.19
NSCAC_1476	sucD	Succinyl-CoA ligase, subunit alpha	115.2	138.5	26.6	28.5	0	0.56	0.75
NSCAC_0929		Malto-oligosyltrehalose synthase	115.1	161.8	4	32.7	0	0.86	0.93
NSCAC_1586		PTS system fructose subfamily IIA component	115.1	218.1	24	73.6	0	0.04	0.13
NSCAC_0571	dapD	2,3,4,5-tetrahydropyridine-2,6-dicarboxylate N-succinyltransferase	115	199.6	46.8	53.7	0	0.17	0.38
NSCAC_1468		protein of unknown function	115	34.7	77.3	23.3	-2	0	0
NSCAC_1336		Glycosyl transferase group 1	114.7	192.2	26.9	7.7	0	0.1	0.26
NSCAC_0060	rpmE	50S ribosomal subunit protein L31	114.7	248.3	77.1	75.8	1	0.11	0.28
NSCAC_0437	prfC	peptide chain release factor RF-3	114.3	182.6	26.5	22.7	0	0.31	0.54
NSCAC_0146		Type II secretion system protein	114	167.3	59	16.8	0	0.59	0.77
NSCAC_1242	thrC	threonine synthase	114	210.9	20.7	24.3	0	0.05	0.17
NSCAC_0856		protein of unknown function	113.8	172.7	24.2	35	0	0.41	0.64
NSCAC_1520	rpiA	ribose 5-phosphate isomerase A	113.2	121.3	47.5	23.2	0	0.26	0.49
NSCAC_0770	cysZ	Protein CysZ homolog	113.1	121	15.3	25.3	0	0.23	0.45

NSCAC_0530	trmD	tRNA (guanine-1-)-methyltransferase	112.8	190.5	55.9	32.9	0	0.29	0.52
NSCAC_0548		protein of unknown function	112.7	248.9	58.4	56.1	1	0.01	0.05
NSCAC_0442		conserved protein of unknown function	112.3	98.8	9.9	21.8	-1	0	0.02
NSCAC_1480	efp	elongation factor P	112.3	105.7	29.8	19.5	-1	0.02	0.08
NSCAC_0872	nhaA	Na(+)/H(+) antiporter NhaA	112.1	109.5	17	16.7	0	0.03	0.12
NSCAC_1025		conserved protein of unknown function	112	125.8	15.7	12	0	0.24	0.47
NSCAC_0836	rsxA	putative inner membrane subunit of an electron transport system	111.8	96.8	59.5	20.3	-1	0.14	0.33
NSCAC_0362	rpsQ	30S ribosomal subunit protein S17	111.8	68.5	44.4	21.2	-1	0	0
NSCAC_0768		Alkyl hydroperoxide reductase/ Thiol specific antioxidant/ Mal allergen	111.3	136.6	74.2	45.6	0	0.84	0.92
NSCAC_0390	lysS	lysine tRNA synthetase, constitutive	111.2	136.4	20.7	27.8	0	0.5	0.71
NSCAC_0708		Oxidoreductase FAD/NAD(P)-binding domain protein	111.2	103.3	14.6	8.8	-1	0.02	0.08
NSCAC_1659		exported protein of unknown function	111	100.4	23.5	11.6	-1	0.02	0.08
NSCAC_1403	adk	Adenylate kinase	110.9	200.9	41.3	60.5	0	0.1	0.26
NSCAC_0109		General secretion pathway protein J	110.7	126	71.9	6.2	0	0.58	0.77
NSCAC_0208	dapB	4-hydroxy-tetrahydrodipicolinate reductase	110.3	196.5	23.8	7.9	0	0.04	0.13
NSCAC_1074		protein of unknown function	110.3	123.1	8	6.5	0	0.19	0.41
NSCAC_0027	dnaG	DNA primase	109.9	198.1	18.7	33.5	0	0.03	0.1
NSCAC_1489	mltB	Membrane-bound lytic murein transglycosylase B	109.9	229.4	26.2	35.7	1	0	0.01
NSCAC_0058	lnt	Apolipoprotein N-acyltransferase	109.9	192.2	22.3	19.5	0	0.16	0.36
NSCAC_0825		conserved protein of unknown function	109.8	33.3	63.5	17.7	-2	0	0
NSCAC_0504		ABC-2 transporter	109.8	93.1	19.4	6.2	-1	0.01	0.06
NSCAC_1132	panB	3-methyl-2-oxobutanoate hydroxymethyltransferase	109.5	161.4	14.9	20.3	0	0.54	0.73
NSCAC_0985		Peptidase S8 and S53 subtilisin kexin sedolisin	109.4	231.3	12.5	9.1	1	0	0
NSCAC_1709	obgE	GTPase Obg	109.3	329.6	28.7	43.7	1	0	0
NSCAC_1758	yciA	Acyl-CoA thioester hydrolase YciA	109.3	169.6	35.9	10.2	0	0.33	0.57
NSCAC_0764		conserved protein of unknown function	109.2	149.9	20.7	8.5	0	0.91	0.96
NSCAC_1361	rppH	RNA pyrophosphohydrolase	109.1	136.5	7.1	55.8	0	0.65	0.81
NSCAC_1227		membrane protein of unknown function	109	109.3	39.9	12.9	0	0.18	0.4
NSCAC_1408	secA	preprotein translocase subunit, ATPase	108.8	214.4	40.1	47.8	1	0.02	0.08
NSCAC_1455	spoT	bifunctional (p)ppGpp synthetase II and guanosine-3',5'-bis pyrophosphate 3'-pyrophosphohydrolase	108.5	235.7	12.7	17.6	1	0	0.02

NSCAC_0718		Acyltransferase 3	108.4	156.9	25	30.1	0	0.61	0.78
NSCAC_1263		Iron-regulated ABC transporter permease protein SufD	108.3	117.9	16.8	25.2	0	0.12	0.29
NSCAC_1300		putative metal transport system ATP-binding protein CT_416	108.2	92.9	26.2	9.7	-1	0.02	0.08
NSCAC_0948		putative Phosphate-selective porin O and P	108.1	165	10.5	24.4	0	0.49	0.7
NSCAC_1618		NAD-dependent epimerase/dehydratase	108.1	144.3	9.9	16.7	0	0.86	0.93
NSCAC_0228	greA	transcription elongation factor	108	89.1	63.3	85.6	-1	0.22	0.45
NSCAC_0807		FAD linked oxidase domain protein	107.3	107.8	20.2	19.1	0	0.08	0.23
NSCAC_0198	tsaC	Threonylcarbamoyl-AMP synthase	107.3	166.8	41.7	63.5	0	0.51	0.72
NSCAC_0973	fabB	3-oxoacyl-[acyl-carrier-protein] synthase I	107.2	133.5	22.1	13.9	0	0.6	0.77
NSCAC_1191	yfjB	NAD kinase	107.2	123.6	11.3	16.4	0	0.29	0.52
NSCAC_1198	dcd	deoxycytidine triphosphate deaminase	107.2	221.4	30.3	53.5	1	0.01	0.04
NSCAC_1470	pilR	Type 4 fimbriae expression regulatory protein PilR	107	78.9	26.1	18.9	-1	0	0.02
NSCAC_1589		conserved membrane protein of unknown function	107	221.5	56	62.5	1	0.21	0.43
NSCAC_1551	prmA	methylase for 50S ribosomal subunit protein L11	107	169.2	18.1	22.6	0	0.27	0.5
NSCAC_1560	trpS	Tryptophan--tRNA ligase	106.9	181.8	41.2	34.2	0	0.21	0.44
NSCAC_1334		Capsular polysaccharide biosynthesis protein CapK	106.8	172.6	34.8	15.7	0	0.23	0.46
NSCAC_0275	glyS	glycine tRNA synthetase, beta subunit	106.5	143.9	20.8	11.4	0	0.95	0.98
NSCAC_1116	gcvH	glycine cleavage complex lipoylprotein	106.3	179.6	47	105.1	0	0.43	0.66
NSCAC_0128		Membrane-bound metal-dependent hydrolase	106.2	140.8	30.2	25.9	0	0.92	0.97
NSCAC_1293		Amino acid-binding ACT domain protein	105.5	169	10.9	35.6	0	0.28	0.51
NSCAC_1082		conserved exported protein of unknown function	105.4	265.6	34.5	66.9	1	0	0
NSCAC_0119		ABC-2 type transporter	105	187.8	11.2	31.8	0	0.08	0.21
NSCAC_1676	tolQ	membrane spanning protein in TolA-TolQ-TolR complex	104.5	139.4	32.6	26.4	0	0.92	0.97
NSCAC_0591		conserved protein of unknown function	104.4	160.3	34.6	41.8	0	0.44	0.66
NSCAC_1007	pheT	phenylalanine tRNA synthetase, beta subunit	104.4	158.2	26.1	8.6	0	0.42	0.65
NSCAC_0014		Rhodanese domain protein (fragment)	104.1	213.4	70.6	63.9	1	0.11	0.27
NSCAC_0735		DNA polymerase III chi subunit HolC	104.1	267.2	35.9	20.1	1	0	0
NSCAC_0018		conserved protein of unknown function	103.9	109.8	18.4	63.6	0	0.25	0.47
NSCAC_0061		Malate dehydrogenase (Oxaloacetate-decarboxylating) (NADP(+))	103.7	228.3	15.1	15.8	1	0	0
NSCAC_1377		putative Beta-lactamase	103.5	52.5	37.7	5.5	-1	0	0
NSCAC_1003		Uracil-xanthine permease	103.4	299.9	24.1	10.5	1	0	0

NSCAC_1749	minC	putative septum site-determining protein MinC	103.4	133.4	16.8	9.3	0	0.74	0.86
NSCAC_0131	leuC	3-isopropylmalate dehydratase, large subunit	103.3	103.8	19.4	6.4	0	0.04	0.14
NSCAC_0943		conserved protein of unknown function	103.3	51.9	55.4	12.7	-1	0	0.01
NSCAC_1561		Peptidase M50	103.1	213.7	1.7	25.4	1	0.01	0.04
NSCAC_1629	purA	adenylosuccinate synthetase	102.7	103.7	27	24.2	0	0.07	0.21
NSCAC_0678	msrA	methionine sulfoxide reductase A	102.6	170.9	7.9	49	0	0.22	0.45
NSCAC_1622	rho	transcription termination factor	102.3	148.9	42.9	24.1	0	0.62	0.78
NSCAC_0016	secB	protein export chaperone	101.9	213.2	52.1	62	1	0.09	0.24
NSCAC_0409	fabZ	(3R)-hydroxymyristol acyl carrier protein dehydratase	101.8	175.4	74.9	35.6	0	0.37	0.6
NSCAC_1419	murD	UDP-N-acetylmuramoyl-L-alanine:D-glutamate ligase	101.6	175.2	11.5	29.7	0	0.16	0.37
NSCAC_1323		Peptidase M16 domain protein	101.2	168	34.3	27.4	0	0.17	0.38
NSCAC_0203		DNA polymerase III epsilon subunit (modular protein)	101.1	194.2	30.4	76.6	0	0.07	0.21
NSCAC_1653	thiE	Thiamine-phosphate synthase	100.5	152.5	19.3	14.5	0	0.47	0.68
NSCAC_1202		Glyoxalase/bleomycin resistance protein/dioxygenase	100.5	94.6	49.1	52	-1	0.22	0.45
NSCAC_0854		conserved membrane protein of unknown function	100.4	147.8	32.2	50.9	0	0.66	0.81
NSCAC_1572		Sulfate transporter / antisigma-factor antagonist STAS	100.1	129.8	98.3	54.5	0	0.99	0.99
NSCAC_1588	phbI	Phosphoenolpyruvate-protein phosphotransferase	99.6	186.5	31.7	19.7	0	0.05	0.15
NSCAC_0056	ybeX	putative protein involved in divalent ion export	99.6	186.8	28.6	40.9	0	0.04	0.12
NSCAC_1678	ruvB	holliday junction helicase, subunit B	98.9	119.1	2.9	13.9	0	0.47	0.68
NSCAC_0334	ubiE	bifunctional 2-octaprenyl-6-methoxy-1,4-benzoquinone methylase and S-adenosylmethionine:2-DMK methyltransferase	98.8	188.6	17.5	17.2	0	0.07	0.21
NSCAC_0566	glcD	glycolate oxidase subunit, FAD-linked	98.8	104	37.9	16.7	0	0.22	0.44
NSCAC_1782	rnpA	Ribonuclease P protein component	98.6	86.8	40.6	37.7	-1	0.1	0.26
NSCAC_1010	rpmI	50S ribosomal subunit protein L35	98.2	73.4	61.6	30.7	-1	0.11	0.27
NSCAC_0156	rpsR	30S ribosomal protein S18	98.1	304.5	55.1	40.2	1	0	0
NSCAC_1682	ilvE	Branched-chain-amino-acid aminotransferase	98	120	67.3	3.5	0	0.85	0.92
NSCAC_0499		conserved protein of unknown function	97.9	60.9	43.6	7.7	-1	0	0.01
NSCAC_0540	folD	bifunctional 5,10-methylene-tetrahydrofolate dehydrogenase and 5,10-methylene-tetrahydrofolate cyclohydrolase	97.8	78.1	27.8	7.8	-1	0.01	0.04
NSCAC_1671		Tol-pal system protein YbgF	97.6	190.3	41.1	85.1	1	0.1	0.26
NSCAC_0790	glcE	glycolate oxidase FAD binding subunit	97.5	61.6	9	8.1	-1	0	0
NSCAC_0139		conserved membrane protein of unknown function, cytochrome oxidase	97.1	97.1	17.3	28.6	0	0.14	0.33

		associated							
NSCAC_1290	purC	phosphoribosylaminoimidazole-succinocarboxamide synthase	96.6	118.5	36.1	58.7	0	0.67	0.82
NSCAC_1644		putative TonB-dependent receptor	96.6	123	11.6	15.2	0	0.68	0.82
NSCAC_0780	yfgD	putative oxidoreductase	96.5	164.7	82.5	51.4	0	0.64	0.8
NSCAC_0882	tyrA	Prephenate dehydrogenase	96.5	132.1	16.5	13.5	0	0.93	0.97
NSCAC_0671		Isoprenylcysteine carboxyl methyltransferase family protein	96.4	116.1	23.4	8.7	0	0.49	0.71
NSCAC_0085	gltX	glutamyl-tRNA synthetase	96.2	139.5	22.1	23.5	0	0.65	0.8
NSCAC_0299		membrane protein of unknown function	96.1	135.5	11.2	11.4	0	0.76	0.87
NSCAC_1410	lpxC	UDP-3-O-acyl N-acetylglucosamine deacetylase	96.1	284.8	35	45.8	1	0	0
NSCAC_1490	gatC	Aspartyl/glutamyl-tRNA(Asn/Gln) amidotransferase subunit C	96	204.6	63.8	56.3	1	0.07	0.2
NSCAC_0779		conserved protein of unknown function	95.9	89.7	34.2	26.3	-1	0.07	0.21
NSCAC_1338	wecB	UDP-N-acetylglucosamine 2-epimerase	95.9	198.1	19.7	13.5	1	0	0.01
NSCAC_1090		putative [NiFe]-hydrogenase, membrane subunit HyfB	95.8	149.8	19.5	22.6	0	0.51	0.71
NSCAC_1693		conserved membrane protein of unknown function	95.4	125.8	43.9	6.3	0	0.87	0.93
NSCAC_1087		putative [NiFe]-hydrogenase, membrane subunit HyfF	94.8	139.2	19.9	5.8	0	0.74	0.86
NSCAC_1569	hisG	ATP phosphoribosyltransferase	94.8	161.2	16.3	15.3	0	0.2	0.42
NSCAC_1197	purN	phosphoribosylglycinamide formyltransferase 1	94.5	148.9	16.4	31	0	0.46	0.67
NSCAC_1701		conserved protein of unknown function	94.5	135	33.1	44.7	0	0.8	0.9
NSCAC_0994	gshA	Glutamate-cysteine ligase	94.3	96.8	8.7	10.8	0	0.08	0.23
NSCAC_0792	accA	acetyl-CoA carboxylase, carboxytransferase, alpha subunit	94	114.6	14.6	12	0	0.46	0.68
NSCAC_1030	icmt	Isoprenylcysteine carboxyl methyltransferase	93.7	104.5	37.6	21.6	0	0.37	0.6
NSCAC_0797	ftsB	cell division protein	93.7	108.1	28.3	75.2	0	0.54	0.73
NSCAC_1120	hisS	histidyl tRNA synthetase	93.6	108	13.8	11.1	0	0.29	0.52
NSCAC_1748	prfA	peptide chain release factor RF-1	93.6	143.2	33.7	57.5	0	0.58	0.76
NSCAC_0663	lpxA	Acyl-[acyl-carrier-protein]--UDP-N-acetylglucosam ine O-acyltransferase	93.6	188.2	18.2	58.4	1	0.04	0.13
NSCAC_1273	trpC	Indole-3-glycerol phosphate synthase	93.4	133.3	35.9	46.3	0	0.77	0.88
NSCAC_1452	ubiA	p-hydroxybenzoate octaprenyltransferase	93.4	140.9	1.7	4.5	0	0.45	0.67
NSCAC_0274	glyQ	glycyl-tRNA synthetase, alpha chain	93.4	178.2	9.2	6.7	0	0.04	0.13
NSCAC_0291	yjeE	ATPase with strong ADP affinity	93.4	103.1	46.9	18.1	0	0.43	0.66
NSCAC_0310		Phosphate ABC transporter, inner membrane subunit PstA	92.7	102.1	11.9	0.2	0	0.19	0.4

NSCAC_1747	hemA	glutamyl tRNA reductase	92.5	145.1	22.7	36.8	0	0.51	0.72
NSCAC_0114		General secretion pathway protein D	92.5	121.2	17.4	10.4	0	0.84	0.92
NSCAC_0567	GLY K	D-glycerate 3-kinase	92.5	107.4	12.8	27.3	0	0.34	0.57
NSCAC_0761		conserved protein of unknown function	92.4	139.5	118	122.5	0	0.87	0.93
NSCAC_1294	ygaF	putative peroxiredoxin YgaF	92.4	140.5	48.2	19	0	0.57	0.75
NSCAC_1356		membrane protein of unknown function	92.3	143.7	15.4	25.8	0	0.31	0.55
NSCAC_1443	nadE	Glutamine-dependent NAD(+) synthetase	92.2	92.4	4.7	10	0	0.08	0.22
NSCAC_0769		conserved protein of unknown function	92.1	84.9	17.5	30.5	-1	0.05	0.16
NSCAC_1697	argB	Acetylglutamate kinase	92	155.6	16.3	45.5	0	0.3	0.54
NSCAC_0907	ycfH	putative metallodependent hydrolase	91.7	144.6	22.2	9.6	0	0.26	0.49
NSCAC_0639		NUDIX hydrolase	91.2	127.1	17.9	2.8	0	0.86	0.93
NSCAC_1704		Efflux transporter, RND family, MFP subunit	91.1	142.3	41.6	31.3	0	0.43	0.66
NSCAC_0812		UbiA prenyltransferase family protein	90.9	145.1	22.6	18.7	0	0.45	0.67
NSCAC_1687		conserved membrane protein of unknown function	90.6	137.9	11.2	4.5	0	0.49	0.7
NSCAC_1562	serS	seryl-tRNA synthetase, also charges selenocysteinyl-tRNA with serine	90.4	221.7	17	35.6	1	0	0
NSCAC_0422	egtD	Histidine-specific methyltransferase EgtD	90.3	66.7	21.8	29.6	-1	0.01	0.03
NSCAC_0158	dnaB	replicative DNA helicase	90.3	182.1	15.5	7.4	1	0	0.02
NSCAC_1364	udg	UDP-glucose 6-dehydrogenase	90.3	116.3	23.2	21	0	0.76	0.87
NSCAC_0852		MatE family protein	90.2	152.5	9.9	13.5	0	0.1	0.26
NSCAC_0459		conserved protein of unknown function	90.1	89.3	9.7	19.4	0	0.04	0.14
NSCAC_0862		Polysaccharide export protein	90	197.7	20.8	64.9	1	0.01	0.03
NSCAC_1316	glgA	Glycogen synthase	89.9	115.3	13.1	13.9	0	0.7	0.83
NSCAC_0012	yajL	Chaperone protein YajL	89.8	191.6	33.5	56.9	1	0.03	0.1
NSCAC_0611		membrane protein of unknown function	89.6	191.4	15	69.2	1	0.03	0.12
NSCAC_0741	purL	phosphoribosylformyl-glycineamide synthetase	89.6	98.3	14.8	8.7	0	0.21	0.44
NSCAC_0416	ispA	geranyltranstransferase	89.5	182.1	22.4	20	1	0	0.02
NSCAC_1138		O-acetyltransferase	89.4	57.1	21.5	16.4	-1	0	0.01
NSCAC_1168		Drug resistance transporter, EmrB/QacA subfamily	89.3	62.4	13.5	1.5	-1	0	0
NSCAC_0320	clpB	protein disaggregation chaperone	89.3	128.3	15.3	15.3	0	0.67	0.82
NSCAC_1318	ggt	Gamma-glutamyltranspeptidase	88.9	110.9	18.6	4.7	0	0.64	0.8

NSCAC_1609		conserved membrane protein of unknown function	88.5	108.4	30.2	21.9	0	0.61	0.78
NSCAC_1330		Asparagine synthetase	88.5	125.3	22.9	51.4	0	0.83	0.91
NSCAC_0138		Cytochrome oxidase biogenesis protein SCO1/SenC	88.5	49.8	20.5	15.7	-1	0	0
NSCAC_0554		Peptidase M48 Ste24p	88.3	77.8	33.3	7.8	-1	0.04	0.14
NSCAC_1778	rsmG	Ribosomal RNA small subunit methyltransferase G	88.2	101.3	17.7	53.3	0	0.43	0.66
NSCAC_0658	dedA	conserved hypothetical protein; putative inner membrane protein	88	69.7	24.9	7.3	-1	0	0.01
NSCAC_1274		conserved membrane protein of unknown function	88	164	37.5	40.1	0	0.29	0.52
NSCAC_0962	ynfA	conserved hypothetical protein; putative inner membrane protein	88	102.7	34.9	41.6	0	0.7	0.84
NSCAC_0159	alr	alanine racemase 1, PLP-binding, biosynthetic	87.7	163.4	24.7	15.8	0	0.14	0.34
NSCAC_0884		HAD-superfamily hydrolase, subfamily IA, variant 1	87.5	170.4	38.3	55.5	1	0.07	0.19
NSCAC_1620		conserved protein of unknown function	87.3	129.5	27.9	35.7	0	0.68	0.83
NSCAC_0407	bamA	Outer membrane protein assembly factor BamA	87.3	149.3	10.8	12.4	0	0.17	0.38
NSCAC_0570	dapE	Succinyl-diaminopimelate desuccinylase	87.3	120.1	12.2	20.4	0	0.92	0.97
NSCAC_0798	ispD	4-diphosphocytidyl-2C-methyl-D-erythritol synthase	87	118.4	14	26	0	0.96	0.98
NSCAC_1736		Sodium/hydrogen exchanger	87	122.6	14.4	11.4	0	0.82	0.91
NSCAC_0806		Short-chain dehydrogenase/reductase SDR	87	84.4	40.8	27.2	0	0.19	0.41
NSCAC_1494		conserved protein of unknown function	86.9	187.6	35.6	19.8	1	0.01	0.06
NSCAC_0269	yqgF	Holliday junction resolvase	86.7	195.1	29.7	11.8	1	0.01	0.04
NSCAC_0432		Sel1 repeat protein (fragment)	86	157.2	52.2	21	0	0.17	0.38
NSCAC_0145	xpsE	Type II secretion system protein E	86	141.8	14.8	20.6	0	0.19	0.41
NSCAC_0593		putative Quercetin 2,3-dioxygenase	85.9	153.4	40.8	35.4	0	0.16	0.36
NSCAC_0476	ureD	Urease accessory protein UreD	85.6	347.6	19.5	91.3	2	0	0
NSCAC_1454		RutC family protein in vnfA 5'region	85.5	236.8	35.1	94.1	1	0	0.01
NSCAC_1556		Squalene/phytoene synthase	85.4	279.7	25.9	38.8	1	0	0
NSCAC_1317	ppc	Phosphoenolpyruvate carboxylase	85.3	120.2	20.9	16.2	0	0.76	0.87
NSCAC_1413	ftsQ	Cell division protein FtsQ	85.3	209	52.6	68	1	0.02	0.07
NSCAC_1465	yqaA	Inner membrane protein YqaA	85.2	125.4	10.1	39.1	0	0.74	0.86
NSCAC_0271		putative TonB family protein	85.1	233.9	4	21.4	1	0	0
NSCAC_1017		exported protein of unknown function	85.1	69.4	29.6	9.2	-1	0.01	0.05
NSCAC_1088		putative [NiFe]-hydrogenase, membrane subunit HyfE	84.9	110.6	35.8	3.1	0	0.85	0.92
NSCAC_0928		conserved protein of unknown function	84.4	87.3	26.2	22.4	0	0.2	0.42

NSCAC_1254	nadB	L-aspartate oxidase	84.3	132.7	20.7	4.2	0	0.43	0.66
NSCAC_1349		Sugar transferase, PEP-CTERM system associated	84.3	107.9	27.6	17	0	0.79	0.89
NSCAC_1322		Peptidase M16 domain protein	84.1	100.6	6.4	21.9	0	0.41	0.64
NSCAC_0782		Biotin/acetyl-CoA-carboxylase ligase	83.9	102.3	21.6	17.2	0	0.57	0.75
NSCAC_0321		Invasion gene expression up-regulator, SirB	83.8	168.4	52.4	5.1	1	0.09	0.24
NSCAC_1257		Glycosyl transferase family 4	83.8	98.7	42.1	20.5	0	0.65	0.8
NSCAC_1344		PEP-CTERM system associated protein	83.7	82.9	14.8	11	0	0.12	0.3
NSCAC_0386	galU	UTP--glucose-1-phosphate uridylyltransferase	83.7	134	18.6	13.6	0	0.34	0.58
NSCAC_0898		exported protein of unknown function	83.6	148.5	32.6	85.2	0	0.3	0.53
NSCAC_1734	tatB	Sec-independent protein translocase protein TatB	83.5	91.2	16.3	42.5	0	0.29	0.52
NSCAC_0010	argS	Arginine--tRNA ligase	83.4	80.8	21.3	13.7	0	0.05	0.16
NSCAC_0057	yfeH	putative sodium/bile acid symporter family (mazG-like)	83.4	78.4	9.5	23.5	-1	0.06	0.18
NSCAC_0934	uvrC	excinuclease UvrABC, endonuclease subunit	83.3	115.2	27.3	22.7	0	0.86	0.93
NSCAC_1319		putative ferredoxin [4Fe-4S] (Fdx)	82.8	62.8	74.6	71.2	-1	0.44	0.67
NSCAC_0105	dapF	diaminopimelate epimerase	82.5	145.2	36.2	22.4	0	0.21	0.43
NSCAC_0166		Protein-L-isoaspartate(D-aspartate) O-methyltransferase	81.5	179	8.1	34.9	1	0	0.01
NSCAC_0395	cysM	Cysteine synthase	81.1	154	15.6	29.3	0	0.02	0.08
NSCAC_0556	phnW	2-aminoethylphosphonate--pyruvate transaminase	81	230.5	17.6	23.7	1	0	0
NSCAC_0572		conserved protein of unknown function	80.8	167.8	22.9	20.9	1	0.01	0.03
NSCAC_1124	proS	prolyl-tRNA synthetase	80.8	87.6	13.1	23.2	0	0.18	0.39
NSCAC_1079	leuD	3-isopropylmalate dehydratase, small subunit	80.7	122.3	16.9	23.3	0	0.45	0.67
NSCAC_0205	grpE	Protein GrpE	80.1	166.6	28.1	87	1	0.1	0.25
NSCAC_1692	proC	Pyrroline-5-carboxylate reductase	80.1	92.4	24.7	23.5	0	0.53	0.73
NSCAC_0338	exoB	UDP-glucose 4-epimerase	80	181.4	31.2	7.4	1	0	0.02
NSCAC_1222		Methyltransferase domain family	80	207.4	36.5	30.6	1	0	0.01
NSCAC_0853		Sulfotransferase	80	116.6	1.8	50.6	0	0.8	0.9
NSCAC_0323		exported protein of unknown function	79.8	104.8	31.8	59.9	0	0.89	0.95
NSCAC_0204	hrcA	Heat-inducible transcription repressor HrcA	79.8	148.1	24.9	29.8	0	0.1	0.26
NSCAC_0881	hisC	Histidinol-phosphate aminotransferase	79.6	111.9	9	1.7	0	0.78	0.88
NSCAC_1459		conserved protein of unknown function	79.5	165.6	20.4	48.4	1	0.02	0.07
NSCAC_0582		conserved protein of unknown function	79.5	101.4	32.4	13.9	0	0.86	0.93

NSCAC_0516	ileS	isoleucyl-tRNA synthetase	79.5	121.9	13.2	9.4	0	0.37	0.6
NSCAC_1221		ABC transporter related protein	79.4	154.7	25.8	49.5	1	0.05	0.16
NSCAC_0681		Peptidase M16C associated domain protein	79.2	120.3	9.3	14.7	0	0.5	0.71
NSCAC_1200		conserved protein of unknown function	79.2	170.6	44.1	96	1	0.12	0.29
NSCAC_1389	yihA	GTP-binding protein	79.2	105.5	20.4	11.5	0	0.97	0.99
NSCAC_0104		conserved protein of unknown function	79.2	176.1	14	32.7	1	0.02	0.08
NSCAC_0808		conserved membrane protein of unknown function	78.8	105.9	15	20.7	0	0.92	0.97
NSCAC_0473		ABC transporter, permease component	78.7	151.7	21.7	14	1	0.03	0.1
NSCAC_1536		CDP-alcohol phosphatidyltransferase	78.5	82.6	11.9	17	0	0.2	0.42
NSCAC_0613		UbiE/COQ5 methyltransferase	78.4	146.9	5.6	33	0	0.05	0.16
NSCAC_0871	aroG	Phospho-2-dehydro-3-deoxyheptonate aldolase	78.4	139.4	7	20.2	0	0.13	0.32
NSCAC_1162		putative sugar transporter of the major facilitator superfamily (MFS)	78.3	70.5	10.9	3.8	-1	0.02	0.08
NSCAC_1745	lolB	Outer-membrane lipoprotein LolB	77.6	119.6	32.9	22.8	0	0.65	0.8
NSCAC_1094		protein of unknown function	77.4	103.8	26.5	26.9	0	0.99	0.99
NSCAC_0190		Monooxygenase FAD-binding protein	77.2	99.1	21.3	6	0	0.79	0.89
NSCAC_1201	yhfA	Protein YhfA	77.2	108.2	21.8	19.3	0	0.81	0.9
NSCAC_1417	murG	N-acetylglucosaminyl transferase	77.2	155.6	25.9	35.7	1	0.02	0.09
NSCAC_0813	hslR	Heat shock protein 15 homolog	77.2	240.7	30	36.9	1	0	0
NSCAC_0774	mfd	transcription-repair coupling factor	77.2	112.4	6.3	5.4	0	0.6	0.77
NSCAC_1077		Sulfatase	77.1	79.7	30.1	9.3	0	0.27	0.5
NSCAC_0148		General secretion pathway protein H	77.1	133.3	38.8	1	0	0.42	0.65
NSCAC_0622		conserved membrane protein of unknown function	76.9	95.8	16	23	0	0.64	0.8
NSCAC_1515	proA	Gamma-glutamyl phosphate reductase	76.9	101.2	33.9	9	0	0.93	0.97
NSCAC_1179		protein of unknown function	76.3	39.9	10.5	5.3	-1	0	0
NSCAC_0095		conserved protein of unknown function	76	146	32	27.8	0	0.17	0.39
NSCAC_0412	bamB	Outer membrane protein assembly factor BamB	75.5	163.1	27.1	30	1	0.06	0.18
NSCAC_1253	rpoE	RNA polymerase, sigma 24 (sigma E) factor	75.4	107.6	39.7	22.8	0	0.74	0.86
NSCAC_1592		conserved protein of unknown function	75.3	259.5	40.4	67.4	1	0	0
NSCAC_0272		Surface antigen (D15)	75.1	118.2	18.4	12.1	0	0.38	0.61
NSCAC_1238		Agmatine deiminase	75	138.3	53.2	13.2	0	0.27	0.51
NSCAC_0202		Generic methyl-transferase	74.9	115.7	16.7	16.9	0	0.41	0.64

NSCAC_0911		Aminodeoxychorismate lyase	74.8	162.2	26.6	2	1	0	0.02
NSCAC_1083		Nucleosidase	74.6	158.8	47.2	56.3	1	0.07	0.21
NSCAC_0466		Phospholipase C 4	74.5	256.1	9.4	10.8	1	0	0
NSCAC_1054	lptD	LPS-assembly protein LptD	74.3	99.1	8	22.4	0	0.99	1
NSCAC_1466	pilA	PilA	74	82.8	28.3	27.6	0	0.53	0.73
NSCAC_0126		Glutamine amidotransferase class-I	73.9	93.1	18	49.7	0	0.71	0.85
NSCAC_0103	lysA	Diaminopimelate decarboxylase	73.8	141.3	13.2	11.5	1	0.01	0.06
NSCAC_0788	fpr	Ferredoxin--NADP reductase	73.8	119.6	35.9	59.1	0	0.49	0.71
NSCAC_1597	smf	conserved hypothetical protein	73.7	102.4	6.2	15	0	0.89	0.95
NSCAC_1259	manC	Mannose-1-phosphate guanylyltransferase/mannose-6-phosphate isomerase	73.6	104.9	6.4	9	0	0.77	0.88
NSCAC_0861		putative Polysaccharide export protein	73.5	120.2	30	50.6	0	0.4	0.63
NSCAC_0093	aspS	aspartyl-tRNA synthetase	73.5	86.8	25	9.7	0	0.55	0.74
NSCAC_1670	queE	7-carboxy-7-deazaguanine synthase	73.4	70.6	21	19.8	-1	0.19	0.4
NSCAC_1365	gatB	Aspartyl/glutamyl-tRNA(Asn/Gln) amidotransferase subunit B	73.3	106	31.1	32.5	0	0.73	0.86
NSCAC_1612		conserved membrane protein of unknown function	72.9	85.9	11.1	8.8	0	0.47	0.68
NSCAC_0896		conserved protein of unknown function	72.9	109.6	18.1	26.4	0	0.73	0.86
NSCAC_0535		Thiol-disulfide interchange protein DsbC	72.8	155.5	10.6	11.2	1	0	0.02
NSCAC_1163		putative sugar transporter of the major facilitator superfamily (MFS)	72.7	122.1	7	12.5	0	0.18	0.4
NSCAC_1741	pth	peptidyl-tRNA hydrolase	72.7	88.7	22.2	17.6	0	0.6	0.77
NSCAC_0865		exported protein of unknown function	72.6	101.2	10.6	25	0	0.91	0.96
NSCAC_0956	ligA	DNA ligase, NAD(+)-dependent	72.5	115.7	20.9	13	0	0.28	0.51
NSCAC_0618		Sel1 domain protein repeat-containing protein	72.4	186.6	26	72	1	0	0.01
NSCAC_0786		conserved protein of unknown function	72.3	130.4	24.7	3.6	0	0.27	0.5
NSCAC_1731	hisE	Phosphoribosyl-ATP pyrophosphatase/Phosphoribosyl-AMP cyclohydrolase	72.3	227.7	13.3	58.9	1	0	0
NSCAC_0522		protein of unknown function	72.2	155.5	8.5	32.5	1	0.01	0.05
NSCAC_0471		conserved membrane protein of unknown function	71.7	110.8	19.6	32.7	0	0.62	0.78
NSCAC_1081		Major facilitator superfamily MFS_1	71.7	97.4	14.3	9.3	0	0.97	0.99
NSCAC_0429		protein of unknown function	71.7	41.5	25.6	11.7	-1	0	0.02
NSCAC_0389	prfB	Peptide chain release factor 2	71.2	149.3	13.1	17.5	1	0.01	0.03
NSCAC_1089		putative [NiFe]-hydrogenase, membrane subunit HyfC	70.9	117.5	18.8	7.6	0	0.36	0.6

NSCAC_0110		Type II secretion system protein K	70.9	140.4	3.2	27	1	0.03	0.11
NSCAC_1039	pgm	Phosphoglucomutase	70.8	88.8	15.7	18	0	0.74	0.86
NSCAC_1557	rluB	23S rRNA pseudouridylate synthase	70.7	123.1	35.9	8.7	0	0.26	0.49
NSCAC_0069	aroK	Shikimate kinase	70.7	141.1	43.9	92.7	1	0.25	0.48
NSCAC_0555	pepN	Aminopeptidase N	70.7	95.5	8	11.3	0	0.98	0.99
NSCAC_0292	yjeF	putative carbohydrate kinase	70.7	72	19.6	14.8	0	0.22	0.44
NSCAC_0558		Phosphoenolpyruvate phosphomutase	70.6	117.6	11.1	12.9	0	0.18	0.4
NSCAC_1517		Rare lipoprotein B family	70.6	50.7	24.5	5.3	-1	0	0.02
NSCAC_0426	ribB	3,4-dihydroxy-2-butanone 4-phosphate synthase	70.6	105.4	25.2	18.5	0	0.54	0.73
NSCAC_1401		Methyltransferase type 12	70.5	81.2	25.8	18.5	0	0.5	0.71
NSCAC_1595	topA	DNA topoisomerase 1	70.2	123.1	11.4	22.9	0	0.1	0.26
NSCAC_0837	metG	methionyl-tRNA synthetase	70.1	97.3	9.4	28.9	0	0.93	0.97
NSCAC_0160	radA	repair protein radA homolog	70.1	92.5	18.6	23.2	0	0.95	0.98
NSCAC_0305		Phosphate-selective porin O and P	70.1	59	48.7	3.9	-1	0.17	0.38
NSCAC_0454		Predicted molecular weight phosphotyrosine protein phosphatase	70.1	96.2	15.3	15.2	0	0.92	0.97
NSCAC_0179	yadG	ABC transporter	69.9	114.9	45.3	9.7	0	0.43	0.66
NSCAC_1095		conserved protein of unknown function	69.9	149.1	7.4	58	1	0.03	0.1
NSCAC_1036		conserved protein of unknown function	69.7	75.3	9.2	24	0	0.36	0.6
NSCAC_0258		Excinuclease ABC, A subunit	69.7	63.3	5.6	6.9	-1	0.04	0.13
NSCAC_1416	murC	UDP-N-acetylmuramate:L-alanine ligase	69.7	125.5	14.4	17.2	0	0.11	0.28
NSCAC_1308	argF	ornithine carbamoyltransferase	69.5	81.4	31.7	20	0	0.56	0.75
NSCAC_0803	ampD	N-acetyl-anhydromuranmyl-L-alanine amidase	69.2	47.7	11.9	16.5	-1	0	0.01
NSCAC_0117		PpiC-type peptidyl-prolyl cis-trans isomerase	69.1	143.6	8	41.6	1	0.01	0.05
NSCAC_1117	malQ	4-alpha-glucanotransferase	69.1	168.2	5.3	18.2	1	0	0
NSCAC_0398	map	methionine aminopeptidase	69	119.1	16.8	29.7	0	0.28	0.52
NSCAC_0795	kdsA	2-dehydro-3-deoxyphosphooctonate aldolase	68.6	92.8	36.1	11.7	0	0.91	0.96
NSCAC_0510		Glycosyl transferase family 2	68.5	99.6	1.6	5.3	0	0.64	0.8
NSCAC_0488		conserved membrane protein of unknown function	68.4	50.9	23.7	12.5	-1	0.03	0.1
NSCAC_1247	lepB	Signal peptidase I	68.3	120.6	17.7	44.6	0	0.19	0.41
NSCAC_0335		conserved protein of unknown function	68.3	115.9	22.7	55.3	0	0.36	0.6
NSCAC_1482	gltA	Citrate synthase	68.2	110.1	1.2	16.8	0	0.32	0.55

NSCAC_0425	ribE	Riboflavin synthase	68.1	100.5	44.3	14.6	0	0.68	0.83
NSCAC_0231	folP	7,8-dihydropteroate synthase	68	116.4	16.6	31.6	0	0.31	0.54
NSCAC_0279		protein of unknown function	68	178.8	8.7	41.3	1	0	0
NSCAC_1516		DNA polymerase III, delta subunit	67.9	102.4	21.4	10.3	0	0.5	0.71
NSCAC_1712	murJ	Protein MurJ homolog	67.8	94	10.6	8.7	0	0.96	0.98
NSCAC_1600	fmt	10-formyltetrahydrofolate:L-methionyl-tRNA(fMet) N-formyltransferase	67.8	81.5	17.4	35.4	0	0.63	0.79
NSCAC_0873		Peptidase M61 domain protein	67.6	97.1	17	4.3	0	0.69	0.83
NSCAC_0870		conserved protein of unknown function	67.6	52.4	31.9	15.5	-1	0.07	0.21
NSCAC_0124		Glycosyl transferase family 2 (fragment)	67.5	71.4	7.2	7.1	0	0.16	0.36
NSCAC_0431		conserved protein of unknown function, Beta-lactamase-like	67.3	99.3	18.6	26.3	0	0.67	0.82
NSCAC_1409		conserved protein of unknown function	67.3	110	40.2	51.1	0	0.75	0.87
NSCAC_0979		conserved protein of unknown function	67.2	34.5	30.9	16.5	-1	0	0.01
NSCAC_1052	pdxA	4-hydroxy-L-threonine phosphate dehydrogenase, NAD-dependent	67	66	24.7	13.7	0	0.21	0.43
NSCAC_1069	trpF	N-(5'-phosphoribosyl)anthranilate isomerase	66.8	151.2	25.2	45.8	1	0.01	0.04
NSCAC_0017	gpsA	glycerol-3-phosphate dehydrogenase (NAD+)	66.7	90.2	14.5	10.3	0	0.93	0.97
NSCAC_1636	tadA	tRNA-specific adenosine deaminase	66.7	86.7	29.9	41.8	0	0.9	0.96
NSCAC_1114	anmK	anhydro-N-acetylmuramic acid kinase	66.6	142	15.8	14.9	1	0.03	0.12
NSCAC_1134	pcnB	poly(A) polymerase I	66.5	78	17.7	8.9	0	0.45	0.67
NSCAC_0100	pilD	Type 4 prepilin-like proteins leader peptide-processing enzyme [Includes: Leader peptidase ; N-methyltransferase]	66.5	69.1	31.2	19.6	0	0.4	0.64
NSCAC_1258	cysC	Adenylyl-sulfate kinase	66.5	103.5	27.3	22.9	0	0.47	0.69
NSCAC_1449	bioB	biotin synthase	66.4	93.7	11.2	7.6	0	0.87	0.93
NSCAC_1777		conserved protein of unknown function	66.3	148.3	18.2	36.3	1	0.01	0.03
NSCAC_1637		conserved protein of unknown function	66.3	98.4	7.9	17.1	0	0.6	0.77
NSCAC_1190		DNA repair protein RecN	66.2	141.3	25.8	33.6	1	0.01	0.06
NSCAC_1158	hemH	Ferrochelatase	65.9	131.1	9.3	5.2	1	0.01	0.04
NSCAC_0737		Permease YjgP/YjgQ family protein	65.9	57.1	24.5	2.7	-1	0.05	0.16
NSCAC_1194		Signal peptide protein	65.5	84.8	19.4	25.7	0	0.87	0.93
NSCAC_0600	corA	Magnesium and cobalt transport	65.5	65.2	10.4	12.4	0	0.18	0.39
NSCAC_0960		Carbohydrate kinase	65.3	77.7	3.4	17.7	0	0.59	0.77
NSCAC_1414	ddlB	D-alanine:D-alanine ligase	65.2	200.5	11.4	15.1	1	0	0

NSCAC_0382	cysH	5'-adenylylsulfate reductase (thioredoxin)	65.1	84.5	22.6	12.8	0	0.87	0.94
NSCAC_0307		Phosphate-selective porin O and P	65	142.4	28.5	21.4	1	0.01	0.06
NSCAC_1328		Glycosyl transferase, group 1	65	69.1	21.9	29.4	0	0.31	0.54
NSCAC_1485	dat	D-alanine aminotransferase	64.9	107.8	22.2	20	0	0.27	0.51
NSCAC_1103	recA	DNA strand exchange and recombination protein with protease and nuclease activity.	64.8	121.6	38.3	10.2	1	0.18	0.4
NSCAC_0042		putative Outer membrane efflux protein	64.7	61.8	10.5	11.8	0	0.11	0.28
NSCAC_0200	yibK	putative rRNA methylase	64.6	144.1	17.6	39.1	1	0.02	0.08
NSCAC_1602	trkA	NAD-binding component of TrK potassium transporter	64.5	84.4	21.4	19.4	0	0.93	0.97
NSCAC_0557	ppd	Phosphonopyruvate decarboxylase	64.5	98.2	13	15	0	0.55	0.74
NSCAC_0562		Squalene/phytoene synthase	64.4	97.1	19.4	6.5	0	0.6	0.77
NSCAC_0712	exbD	membrane spanning protein in TonB-ExbB-ExbD complex	64.2	122.8	22.3	48.9	1	0.11	0.28
NSCAC_0250		conserved protein of unknown function	64.2	73.8	7.2	5.3	0	0.39	0.62
NSCAC_0683	hslO	33 kDa chaperonin	64.2	81.6	5.1	14.8	0	0.74	0.86
NSCAC_0814	yheT	putative esterase YheT	64.2	141.6	26.7	11.2	1	0.01	0.06
NSCAC_0143	rnr	exoribonuclease R, RNase R	64.2	154.5	17.1	3.3	1	0	0
NSCAC_0968		Phosphomethylpyrimidine kinase	63.9	55.7	8.6	7.2	-1	0.03	0.1
NSCAC_1467		Fimbrial protein	63.9	80.9	5.5	3.9	0	0.71	0.85
NSCAC_1487		RlpA-like protein (fragment)	63.9	101.2	40.5	22.5	0	0.77	0.88
NSCAC_0438		Peptidase M28 (fragment)	63.7	136.9	20.9	5.3	1	0.01	0.03
NSCAC_0169	waaD	ADP-L-glycero-D-mannoheptose-6-epimerase, NAD(P)-binding	63.6	125.5	19.7	18.3	1	0.03	0.12
NSCAC_1219		conserved protein of unknown function	63.6	127.9	20	14.2	1	0.02	0.07
NSCAC_0533	xerD	site-specific tyrosine recombinase	63.5	158.2	10.7	20.2	1	0	0
NSCAC_1613		conserved membrane protein of unknown function	62.9	146.3	23.3	7.7	1	0.01	0.06
NSCAC_1599	def	peptide deformylase	62.9	92.8	25.3	39.9	0	0.71	0.85
NSCAC_0267	pyrC	Dihydroorotase-like protein	62.8	103.2	19.7	6.7	0	0.28	0.51
NSCAC_0113		conserved protein of unknown function	62.6	95.4	55.9	17.5	0	0.71	0.85
NSCAC_1252		Anti sigma-E protein RseA, N-terminal domain	62.6	103.6	39.4	46.3	0	0.5	0.71
NSCAC_0254		putative Uncharacterized inner membrane transporter yiJE	62.4	66.3	45.1	25	0	0.47	0.69
NSCAC_1638	guaA	GMP synthetase (glutamine aminotransferase)	62.2	77.6	16.3	19.8	0	0.67	0.82
NSCAC_0949		conserved protein of unknown function	62.1	110.6	35.6	20.6	0	0.31	0.54

NSCAC_0923		putative Sporulation domain protein	61.8	85.5	29.8	56.9	0	0.98	0.99
NSCAC_0615		putative N-hydroxyarylamine O-acetyltransferase	61.8	109.3	32.4	30	0	0.23	0.46
NSCAC_1518	leuS	leucyl-tRNA synthetase	61.7	66.1	27.5	6.9	0	0.39	0.62
NSCAC_0515		conserved protein of unknown function	61.7	94.3	35.5	38.2	0	0.82	0.9
NSCAC_0620		conserved protein of unknown function	61.6	124.2	25.5	14.3	1	0.06	0.19
NSCAC_1448	bioF	8-amino-7-oxononanoate synthase	61.5	102.5	6	30.3	0	0.38	0.61
NSCAC_1373		Glycosyl transferase family 9	61.4	81.5	13.5	7.5	0	0.87	0.94
NSCAC_0312	phoU	negative regulator of PhoR/PhoB two-component regulator	61.3	110.6	5.9	15.6	0	0.09	0.24
NSCAC_1078	leuB	3-isopropylmalate dehydrogenase	61.3	62.8	30.9	5.7	0	0.37	0.61
NSCAC_0617		conserved protein of unknown function	60.9	31.5	11.2	49	-2	0.37	0.61
NSCAC_0624		putative Spore coat protein CotH	60.9	144.1	19.5	11.5	1	0	0.01
NSCAC_1207		conserved membrane protein of unknown function	60.8	72.5	14.4	9.6	0	0.56	0.74
NSCAC_1151	acrR	TetR/AcrR family transcriptional regulator, acrAB operon repressor	60.7	122	31.2	66	0	0.3	0.54
NSCAC_1175		Efflux transporter, RND family, MFP subunit	60.6	78.7	11.6	20.4	0	0.77	0.88
NSCAC_1519	CHA1	L-threonine ammonia-lyase	60.5	81.3	10	11.2	0	0.94	0.97
NSCAC_0646		CRISPR-associated protein, Csy4 family	60.5	96.3	40	12.9	0	0.75	0.87
NSCAC_0170	rfaE	fused heptose 7-phosphate kinase ; heptose 1-phosphate adenyltransferase	60.2	110	10.6	15.3	0	0.08	0.21
NSCAC_1047		DNA protecting protein DprA	60.2	91.6	13.7	72.8	0	0.79	0.89
NSCAC_1759		conserved protein of unknown function	60.1	88.8	9.2	11	0	0.59	0.77
NSCAC_1652	rubA	rubredoxin	60.1	132.7	32.6	22.6	1	0.13	0.31
NSCAC_0713	czcA	Cation efflux system protein CzcA	60	76.7	14	9	0	0.73	0.86
NSCAC_0309		Binding-protein-dependent transport systems inner membrane component	59.9	85	3.9	5.8	0	0.8	0.9
NSCAC_0793	tilS	tRNA(Ile)-lysidine synthase	59.7	81.1	18.3	3.3	0	0.95	0.98
NSCAC_1598		Peptidoglycan-binding LysM (fragment)	59.6	119.6	29.2	22.7	1	0.06	0.18
NSCAC_1433		FAD linked oxidase domain protein	59.5	154.3	15.3	26.5	1	0	0
NSCAC_1542		Pentapeptide repeat protein	59.5	72.7	39.5	37	0	0.81	0.9
NSCAC_1415	murB	UDP-N-acetylenolpyruvoylglucosamine reductase	59.4	139	18.7	6.9	1	0	0.01
NSCAC_0569		conserved protein of unknown function	59.2	110	4.8	22.1	0	0.12	0.3
NSCAC_1699	dut	deoxyuridine 5'-triphosphate nucleotidohydrolase	59.2	108	22.1	30	0	0.22	0.44
NSCAC_1425	mraW	S-adenosyl-dependent methyltransferase activity on membrane-located substrates	59.1	150.5	14	32.8	1	0	0

NSCAC_0448	corA	magnesium/cobalt transporter	59	125	1.2	20.3	1	0.01	0.03
NSCAC_1382		putative O-antigen polymerase	58.8	102.8	22.4	9.5	0	0.15	0.36
NSCAC_0144	psd	Phosphatidylserine decarboxylase proenzyme	58.7	134.8	16.1	19.3	1	0.02	0.09
NSCAC_0843		Tetratricopeptide repeat domain protein	58.6	112.2	12.3	11	1	0.04	0.12
NSCAC_1080		Peptidase M50 (fragment)	58.5	80.2	15.5	10.5	0	0.98	0.99
NSCAC_0047		conserved protein of unknown function	58.5	92.2	10.4	17.2	0	0.52	0.72
NSCAC_0585		conserved protein of unknown function	58.4	86.2	24.6	16.1	0	0.62	0.78
NSCAC_1630		putative DNA polymerase III, subunits gamma and tau	58.2	66.7	18.6	3.8	0	0.42	0.66
NSCAC_0302		Peptidase M23	58.1	72.6	29.4	13	0	0.79	0.89
NSCAC_0403	ispU	undecaprenyl pyrophosphate synthase	58.1	111.9	26.4	20.4	1	0.1	0.26
NSCAC_0891		protein of unknown function	57.9	76.5	16.4	55.8	0	0.86	0.93
NSCAC_0989	gor	Glutathione reductase	57.9	77.1	12.2	12.4	0	0.99	1
NSCAC_0841		Protein-tyrosine kinase	57.7	102	11.9	24.6	0	0.13	0.32
NSCAC_1023		protein of unknown function	57.7	5.8	26.8	10	-4	0.07	0.2
NSCAC_0002	gyrB	DNA gyrase, subunit B	57.7	70.6	9	34	0	0.61	0.78
NSCAC_0040		conserved membrane protein of unknown function	57.6	57.5	23.2	15.9	0	0.23	0.45
NSCAC_1045		conserved protein of unknown function	57.6	107.5	29.1	38.1	1	0.21	0.43
NSCAC_1594	purE	N5-carboxyaminoimidazole ribonucleotide mutase	57.3	90	11.3	26.6	0	0.48	0.69
NSCAC_1234	rph	Ribonuclease PH	57.3	63.1	17.4	16.9	0	0.34	0.58
NSCAC_1251		Sigma E regulatory protein, MucB/RseB	57.3	96.1	3.7	25.8	0	0.32	0.55
NSCAC_0098		DNA gyrase inhibitor YacG (fragment)	57	130.1	33.4	52.6	1	0.19	0.4
NSCAC_0108		conserved protein of unknown function	56.6	91.2	10.5	9.5	0	0.29	0.52
NSCAC_0467		protein of unknown function	56.6	83.2	40.5	33.4	0	0.79	0.89
NSCAC_0635		putative thiol-disulfide oxidoreductase DCC	56.4	62.7	3.6	6.6	0	0.31	0.54
NSCAC_0691		Response regulator containing a CheY-like receiver domain and an HTH DNA-binding domain	56.3	58.1	14	13.9	0	0.29	0.52
NSCAC_0538	gltX	Glutamate-tRNA ligase	56.3	91.6	8.5	21.2	0	0.39	0.62
NSCAC_0322	ybbO	Uncharacterized oxidoreductase YbbO	56.2	90.3	4.4	10.4	0	0.39	0.62
NSCAC_1654		MscS Mechanosensitive ion channel	56.2	59.2	11.5	26	0	0.28	0.51
NSCAC_0266		Dihydroorotate oxidase B, electron transfer subunit	56	53.3	15	2.6	-1	0.09	0.24
NSCAC_0910	tmk	Thymidylate kinase	56	82.1	11.7	23	0	0.67	0.82

NSCAC_1347		Polysaccharide export protein, PEP-CTERM sytem-associated	55.7	82.7	21.7	15.9	0	0.84	0.92
NSCAC_1205	speD	S-adenosylmethionine decarboxylase	55.7	101.4	18.3	19	0	0.11	0.27
NSCAC_1235	yggV	dITP/XTP pyrophosphatase	55.6	75.9	9.8	20.5	0	0.99	0.99
NSCAC_1511	rng	ribonuclease G	55.6	95.5	14.4	8.3	0	0.17	0.38
NSCAC_1283		Phage SPO1 DNA polymerase-related protein	55.4	110.4	36.9	9.1	1	0.29	0.52
NSCAC_0414	folE	GTP cyclohydrolase FolE2	55.3	134	33.8	27.1	1	0.01	0.06
NSCAC_1596	smg	conserved hypothetical protein	55.3	120.3	12.6	31.2	1	0.02	0.09
NSCAC_1070	truA	pseudouridylate synthase I	55.3	76.8	11.3	29.8	0	0.85	0.92
NSCAC_0581		Glutathione S-transferase, N-terminal domain	54.9	99.5	20.1	24.5	0	0.33	0.56
NSCAC_1141		Type III restriction system methylase (fragment)	54.8	43.6	23	25.4	-1	0.13	0.31
NSCAC_1142		conserved protein of unknown function	54.8	62.5	14.4	16	0	0.44	0.66
NSCAC_0630		exported protein of unknown function	54.8	73.7	14.5	8.5	0	0.93	0.97
NSCAC_1002		putative Cyanuric acid amidohydrolase	54.6	213.4	5.8	48.4	2	0	0
NSCAC_0049	thyA	thymidylate synthetase	54.6	104.9	33	21.9	1	0.22	0.44
NSCAC_1583		PTS IIA-like nitrogen-regulatory protein PtsN	54.6	135.1	18.3	30.9	1	0	0.02
NSCAC_0920		protein of unknown function	54.5	93.2	10.1	37	0	0.29	0.52
NSCAC_1400		Peptidase, S49 (Protease IV) family	54.2	132.2	17	28	1	0	0.01
NSCAC_0849		DNA polymerase, beta domain protein region (fragment)	54.2	55.8	33.8	34.2	0	0.47	0.68
NSCAC_1241		conserved protein of unknown function	54.2	51.2	47.6	17.6	0	0.8	0.9
NSCAC_1614		Mov34/MPN/PAD-1 family protein	54.1	73	15.3	17.7	0	0.96	0.98
NSCAC_1345		Protein-tyrosine kinase	54	104.8	27.1	31.3	1	0.12	0.29
NSCAC_0413	der	GTPase involved in ribosome synthesis and maintenance	54	133.1	7.9	21.9	1	0	0.01
NSCAC_1131	panC	pantothenate synthetase	54	83.4	18.2	30.5	0	0.57	0.75
NSCAC_0661	degT	Pleiotropic regulatory protein	53.9	147.5	20.3	27.2	1	0	0.02
NSCAC_1204	ybbJ	conserved hypothetical protein; putative inner membrane protein	53.9	193.4	25.7	54.4	1	0	0
NSCAC_1422	murE	UDP-N-acetylmuramoyl-L-alanyl-D-glutamate--2,6-di aminopimelate ligase	53.9	68.6	14	5.6	0	0.8	0.9
NSCAC_1458	yicC	conserved hypothetical protein	53.7	97.4	13.8	27.6	0	0.16	0.37
NSCAC_1756	argH	Argininosuccinate lyase	53.6	68.9	12.5	2.4	0	0.8	0.9
NSCAC_0721		ATP/GTP-binding protein	53.6	69.4	8.1	16.8	0	0.85	0.92
NSCAC_0805		HAD-superfamily hydrolase, subfamily IB	53.5	92	17.2	7.9	0	0.28	0.52

NSCAC_1060		conserved membrane protein of unknown function	53.4	91.2	19.5	15.7	0	0.28	0.51
NSCAC_1281	serB	Phosphoserine phosphatase	53.3	89.8	24.1	40.8	0	0.39	0.62
NSCAC_1188	bamE	Outer membrane protein assembly factor BamE	53.2	90.5	17.7	55	0	0.46	0.68
NSCAC_1092		putative Fatty acid desaturase	53	65.6	22.4	6.4	0	0.72	0.85
NSCAC_1385	speE	Spermidine synthase	52.7	54.2	15.3	2	0	0.24	0.47
NSCAC_0653		protein of unknown function	52.7	74.4	21.1	11.5	0	0.8	0.9
NSCAC_0385		Polysaccharide biosynthesis protein CapD	52.6	78.7	14.4	27.8	0	0.67	0.82
NSCAC_0982		putative enzyme	52.5	81.9	11.7	36.8	0	0.68	0.82
NSCAC_0318		conserved protein of unknown function	52.4	94.8	32.3	42.7	0	0.53	0.73
NSCAC_1512	ybeA	conserved hypothetical protein	52.4	108.1	19.3	22.5	1	0.09	0.23
NSCAC_1029	arsH	NADPH-dependent FMN reductase	52.2	119.9	18.9	19.2	1	0.06	0.17
NSCAC_0765		putative Membrane protein	52.1	89.5	5.2	7.5	0	0.23	0.46
NSCAC_0313		conserved membrane protein of unknown function	52.1	70.2	17.7	4.6	0	0.95	0.98
NSCAC_0025	rpsU	30S ribosomal protein S21	52	53.3	15.1	24.2	0	0.37	0.61
NSCAC_0952		conserved protein of unknown function	52	64.7	13.6	21	0	0.73	0.86
NSCAC_0086		conserved hypothetical protein	51.9	147.5	34.2	42.7	1	0.01	0.04
NSCAC_0509		Glycosyl transferase family 2	51.9	90.1	15.7	14	0	0.24	0.46
NSCAC_0659		Alpha/beta hydrolase fold hydrolase or acyltransferase	51.7	87.3	0.9	15.7	0	0.26	0.49
NSCAC_1503	mutM	formamidopyrimidine/5-formyluracil/ 5-hydroxymethyluracil DNA glycosylase	51.3	97.1	4.3	23.3	0	0.09	0.25
NSCAC_1193	recJ	Single-stranded-DNA-specific exonuclease RecJ	51.3	60.9	13.5	12.8	0	0.64	0.8
NSCAC_0606		Iron permease FTR1	51.2	80.6	14.4	14	0	0.44	0.66
NSCAC_1513	ybeB	hypothetical protein	51.1	63.3	27.1	57.9	0	0.69	0.83
NSCAC_1315	rmlB	dTDP-D-glucose-4,6-dehydratase	51.1	62.5	9.2	12.5	0	0.6	0.77
NSCAC_0303	nudE	ADP compounds hydrolase NudE	51	136.8	15.9	17	1	0	0
NSCAC_0977		UvrD/REP helicase	51	58.2	14.3	5.7	0	0.43	0.66
NSCAC_1228		protein of unknown function	50.9	82.8	4.2	13.3	0	0.39	0.62
NSCAC_0194	mak	Mannofructokinase	50.9	49.3	14.2	11.9	0	0.17	0.38
NSCAC_1658		conserved exported protein of unknown function	50.8	39.2	19.9	4.5	-1	0.05	0.16
NSCAC_0024	gcp	O-sialoglycoprotein endopeptidase	50.8	61.2	24.2	9.7	0	0.61	0.78
NSCAC_1050	yceF	hypothetical protein	50.7	153.8	42.3	44.3	1	0.01	0.04

NSCAC_1543	rsmJ	Ribosomal RNA small subunit methyltransferase J	50.7	51.2	4.9	7.5	0	0.2	0.42
NSCAC_0268	pyrB	Aspartate carbamoyltransferase	50.7	101.2	13.1	10.7	1	0.03	0.1
NSCAC_0489	ybbA	putative transporter subunit: ATP-binding component of ABC superfamily	50.6	40.3	24.7	15	-1	0.08	0.21
NSCAC_1585	yhbJ	hypothetical protein; putative nucleoside triphosphate hydrolase domain	50.3	92.9	19.3	19.7	0	0.3	0.53
NSCAC_1321	yhhF	putative methyltransferase	50.1	89.8	15.3	5.5	0	0.24	0.46
NSCAC_0749		ABC transporter related protein	50.1	54.8	15	12.4	0	0.35	0.59
NSCAC_1166	nagZ	Beta-hexosaminidase	50	73.5	5.1	12.6	0	0.67	0.82
NSCAC_0811		membrane protein of unknown function	49.8	98.4	11.1	33.6	1	0.07	0.21
NSCAC_1240		transposase (fragment)	49.6	172.1	14	38.5	1	0	0
NSCAC_1383		Glycosyl transferase family 9	49.4	66.3	5	18.6	0	0.93	0.97
NSCAC_1346		Polysaccharide chain length determinant protein, PEP-CTERM locus subfamily	49.3	107	6.6	37.8	1	0.03	0.1
NSCAC_1472		conserved protein of unknown function	49.2	19.7	20.6	10.6	-2	0	0.02
NSCAC_0709		putative C4-dicarboxylate transporter protein	49.2	41	4.9	3.3	-1	0.04	0.12
NSCAC_0875	apt	Adenine phosphoribosyltransferase	48.6	82.7	29.8	26.3	0	0.47	0.68
NSCAC_0249	rluD	23S rRNA pseudouridine synthase	48.6	88.8	33.9	22.3	1	0.31	0.54
NSCAC_0539	cysS	cysteinyl-tRNA synthetase	48.5	91.2	16.9	18.6	0	0.18	0.39
NSCAC_0411		conserved protein of unknown function	48.4	124.6	22.1	89	1	0.06	0.19
NSCAC_1679	ruvA	component of RuvABC resolvasome, regulatory subunit	48.4	50.8	17.2	11.6	0	0.39	0.62
NSCAC_1691	yggS	putative enzyme	48.3	116	33.4	18.9	1	0.05	0.16
NSCAC_1291		NlpB/DapX lipoprotein	48.3	84.5	12.9	36.5	0	0.32	0.55
NSCAC_0549	relA	GTP pyrophosphokinase	48.2	69.6	18.3	14.4	0	0.75	0.87
NSCAC_1524		DNA internalization-related competence protein ComEC/Rec2	48.2	55.2	4	14.3	0	0.5	0.71
NSCAC_1386	speA	Biosynthetic arginine decarboxylase	48.1	64.6	4.6	9.6	0	0.96	0.98
NSCAC_0273		conserved protein of unknown function	48.1	77.5	15	5.4	0	0.35	0.58
NSCAC_0508		conserved protein of unknown function	48.1	51.6	14.8	13.8	0	0.37	0.6
NSCAC_1698	algC	Phosphomannomutase/phosphoglucomutase	48	115.6	10.4	28.3	1	0	0.01
NSCAC_0064		Type IV pilus assembly protein PilM	47.9	100	2.4	28.4	1	0.05	0.16
NSCAC_1590		CHRD domain containing protein (fragment)	47.8	352.5	11.4	154.2	2	0	0
NSCAC_1666	yliA	putative peptide transport fused subunits of ABC superfamily: ATP-binding components	47.7	65.6	3.4	8.5	0	0.94	0.98

NSCAC_1332		Polysaccharide deacetylase	47.7	71.2	20.3	21.1	0	0.64	0.8
NSCAC_0685		conserved protein of unknown function	47.5	60.2	13.3	9.3	0	0.73	0.86
NSCAC_0722	stiP	Cysteine protease StiP	47.5	57.3	14.7	9.4	0	0.6	0.77
NSCAC_1567	hisC	Histidinol-phosphate aminotransferase	47.5	79.1	25.1	5.7	0	0.39	0.62
NSCAC_0020		2-amino-4-hydroxy-6-hydroxymethyldihydropteridine pyrophosphokinase	47.5	63.1	15.9	31.1	0	0.92	0.97
NSCAC_0043	crcB	Protein CrcB homolog	47.5	90.5	13.7	11.2	0	0.11	0.28
NSCAC_0282	mutL	DNA mismatch repair protein MutL	47.4	83.2	5.8	2.3	0	0.17	0.38
NSCAC_0294	orn	oligoribonuclease	47.1	79	8.8	7.3	0	0.25	0.48
NSCAC_1479	epmA	Elongation factor P--(R)-beta-lysine ligase	47.1	88.7	28	19	1	0.23	0.46
NSCAC_0063	mrcA	fused penicillin-binding protein 1a: murein transglycosylase ; murein transpeptidase	46.9	81.4	4	28.6	0	0.31	0.54
NSCAC_1246	rnc	RNase III	46.7	72.2	4	16.7	0	0.49	0.71
NSCAC_0055	ybeY	putative metal-dependent hydrolase	46.5	52.7	5	12.8	0	0.4	0.64
NSCAC_0831	rsxE	putative inner membrane NADH-quinone reductase	46.4	63.1	18.3	12.5	0	0.91	0.96
NSCAC_0936		DNA/RNA non-specific endonuclease	46.1	74	29.9	9.3	0	0.51	0.72
NSCAC_0486		TonB-dependent receptor	45.9	52.4	6.6	4.3	0	0.46	0.68
NSCAC_1440	yhdG	Uncharacterized amino acid permease YhdG	45.8	39.9	7	7.4	-1	0.08	0.22
NSCAC_0443		NnrS protein	45.7	97.2	10.5	5.6	1	0.01	0.06
NSCAC_0961		AMP-dependent synthetase and ligase	45.6	56.2	9.2	10.1	0	0.63	0.79
NSCAC_1582	rpoN	RNA polymerase, sigma 54 (sigma N) factor	45.6	59.8	12.2	16.5	0	0.86	0.93
NSCAC_0380	cysB	HTH-type transcriptional regulator CysB	45.6	99.3	21	11.6	1	0.03	0.11
NSCAC_0224	aroE	Shikimate dehydrogenase	45.5	82.4	14.3	3.6	0	0.24	0.46
NSCAC_0598		conserved exported protein of unknown function	45.5	1091.7	10	349.8	4	0	0
NSCAC_0511	fcl	GDP-L-fucose synthase	45.4	70.6	14.2	22.9	0	0.59	0.77
NSCAC_1606		conserved protein of unknown function	45.3	83.2	13.7	16.8	0	0.28	0.51
NSCAC_1358		conserved protein of unknown function	45.1	95.9	15.5	21.7	1	0.12	0.29
NSCAC_1525	msbA	fused lipid transporter subunits of ABC superfamily: membrane component ; ATP-binding component	45.1	66.3	22.3	10.7	0	0.66	0.81
NSCAC_1463	macB	fragment of fused macrolide transporter subunits of ABC superfamily: ATP-binding component ; membrane component (part 2)	45	77.1	8.1	8.3	0	0.28	0.51
NSCAC_1579		conserved protein of unknown function	44.9	119.6	37.4	19.4	1	0.02	0.09

NSCAC_1696	pyrE	orotate phosphoribosyltransferase	44.9	103.9	15.8	23.3	1	0.02	0.09
NSCAC_1431	yraO	DnaA initiator-associating factor for replication initiation	44.8	67.2	22.5	21.4	0	0.63	0.79
NSCAC_1372		putative Fatty acid desaturase	44.8	96.3	29.9	31.7	1	0.1	0.26
NSCAC_1445	bioD	dethiobiotin synthetase	44.1	30.2	12	12.3	-1	0.02	0.07
NSCAC_1167		ErfK/YbiS/YcfS/YnhG family protein	44	18.1	40.1	17.9	-2	0.11	0.28
NSCAC_1376		conserved protein of unknown function	43.9	42.2	11.4	15.4	0	0.28	0.51
NSCAC_0088		protein of unknown function	43.8	43.9	38	21.7	0	0.8	0.9
NSCAC_1584		HPr kinase	43.8	96	19.5	21.2	1	0.12	0.3
NSCAC_1245	era	membrane-associated, 16S rRNA-binding GTPase	43.8	67.6	27.7	33.3	0	0.67	0.82
NSCAC_0111		Fimbrial assembly family protein	43.8	110.1	15	6.5	1	0.01	0.06
NSCAC_0833	rsxD	putative inner membrane oxidoreductase	43.7	47.9	9.5	12.9	0	0.45	0.67
NSCAC_0602	czcA	Cation efflux system protein CzcA	43.6	59.7	5.6	11.3	0	0.95	0.98
NSCAC_1429		LppC family lipoprotein	43.6	41.8	7	7.8	-1	0.12	0.29
NSCAC_0472		ABC transporter, ATPase component	43.5	66.2	7.6	17.9	0	0.59	0.77
NSCAC_0490	tesA	Esterase TesA	43.4	72.1	14	12.2	0	0.32	0.55
NSCAC_0662	lpxB	Lipid-A-disaccharide synthase	43.3	56.6	12.2	3.9	0	0.91	0.97
NSCAC_0751	yfhQ	putative methyltransferase	43.2	33.9	8.3	3.4	-1	0.03	0.11
NSCAC_1063	trmB	tRNA (guanine-N(7)-)-methyltransferase	43.2	49.9	13.5	9.3	0	0.51	0.72
NSCAC_0686	alkB	oxidative demethylase of N1-methyladenine or N3-methylcytosine DNA lesions	43	101.8	19.9	40.7	1	0.04	0.14
NSCAC_1033	ycbL	putative metal-binding hydrolase	43	49.4	14.3	22.5	0	0.65	0.81
NSCAC_0576	rbfA	Ribosome-binding factor A	42.7	49.3	26	19.3	0	0.62	0.78
NSCAC_0418	ribF	Riboflavin biosynthesis protein RibF [Includes: Riboflavin kinase ; FMN adenylyltransferase]	42.6	59.5	7.4	8.1	0	0.89	0.95
NSCAC_1313	rmlC	dTDP-4-deoxyrhamnose-3,5-epimerase	42.6	77	32.8	34.1	0	0.36	0.6
NSCAC_0150	qacF	Quaternary ammonium compound-resistance protein QacF	42.5	97.9	38.4	54.3	1	0.18	0.4
NSCAC_0191		L-lysine 6-oxidase (fragment)	42.5	58.8	11	6.6	0	0.99	0.99
NSCAC_1423	ftsI	transpeptidase involved in septal peptidoglycan synthesis (penicillin-binding protein 3)	42.4	91.5	2.2	4	1	0.01	0.05
NSCAC_0999		protein of unknown function	42.3	96.4	13.3	5.9	1	0.01	0.04
NSCAC_0874		protein of unknown function	42.1	8.6	21.8	14.9	-3	0.24	0.46
NSCAC_1352	rep	DNA helicase and single-stranded DNA-dependent ATPase	42.1	70.5	11.5	10.9	0	0.4	0.63

NSCAC_1354		conserved protein of unknown function	42	116.3	19.4	56.5	1	0.01	0.05
NSCAC_0300	lolA	Outer-membrane lipoprotein carrier protein	42	70.6	32.2	19.2	0	0.49	0.7
NSCAC_0133		LysM domain protein (modular protein)	42	55.5	16.9	13.2	0	0.97	0.99
NSCAC_0283		exported protein of unknown function	42	69.2	9.7	13.7	0	0.45	0.67
NSCAC_0710		Outer membrane protein	41.9	74.8	14.1	17.1	0	0.18	0.4
NSCAC_1144		Peptidase propeptide and YPEB domain protein	41.9	86.8	21.8	35.9	1	0.13	0.32
NSCAC_0532		Methylated-DNA/protein-cysteine methyltransferase	41.7	116.1	24.3	22.7	1	0.01	0.03
NSCAC_1457	gmk	guanylate kinase	41.7	90.4	8.3	20.1	1	0.05	0.15
NSCAC_0444		conserved protein of unknown function	41.7	93.8	12.5	13	1	0.04	0.14
NSCAC_0908		Type 4 fimbrial biogenesis protein PilZ	41.7	75	36.6	17.8	0	0.74	0.86
NSCAC_0417	xseB	Exodeoxyribonuclease 7 small subunit	41.4	27.1	41.8	15.6	-1	0.37	0.6
NSCAC_0120		ABC transporter, ATPase subunit	41.1	27.7	10.8	3.4	-1	0.01	0.06
NSCAC_0965		Abortive infection protein	40.9	94.3	10.6	35	1	0.03	0.11
NSCAC_0311	pstB	high-affinity phosphate transport protein (ABC superfamily, atp_bind)	40.4	58.2	17.2	11.4	0	0.8	0.9
NSCAC_0288		BNR/Asp-box repeat domain protein	40	30.1	15.8	8.7	-1	0.08	0.23
NSCAC_1195		conserved exported protein of unknown function	39.8	63.9	8.1	25.7	0	0.52	0.72
NSCAC_0847		conserved protein of unknown function	39.7	71.6	11.2	37.5	0	0.34	0.57
NSCAC_0306		Phosphate-selective porin O and P	39.7	104.7	9.7	10	1	0	0.02
NSCAC_0832	rsxG	putative oxidoreductase	39.6	17.7	17.5	13.3	-2	0.02	0.07
NSCAC_0505		ABC transporter	39.3	53.9	8.8	19.6	0	0.99	0.99
NSCAC_1112	tyrS	Tyrosine--tRNA ligase	39.1	58.8	10.9	21.4	0	0.76	0.87
NSCAC_0122		Transferase hexapeptide repeat containing protein	39.1	47.2	23.8	13	0	0.7	0.84
NSCAC_1674		Cell division and transport-associated protein TolA	39	93.2	5.9	10.4	1	0	0.02
NSCAC_1623	miaA	delta(2)-isopentenylpyrophosphate tRNA-adenosine transferase	38.7	34.3	15.7	4.4	-1	0.17	0.39
NSCAC_0997		conserved protein of unknown function	38.7	64.1	14.5	2.3	0	0.53	0.73
NSCAC_1236	yggW	putative oxidoreductase	38.6	79.5	18	20.7	1	0.24	0.46
NSCAC_1587	ptsH	Phosphocarrier protein HPr	38.4	66.8	19	7.9	0	0.3	0.53
NSCAC_0632	valS	valyl-tRNA synthetase	38.4	43.5	3.5	3.5	0	0.44	0.66
NSCAC_0791	dnaE	DNA polymerase III alpha subunit	38.3	47.3	8.7	10.1	0	0.7	0.84
NSCAC_1615	moeB	Molybdopterin-synthase adenylyltransferase	38.3	55.2	20.1	16.6	0	0.76	0.87
NSCAC_0654		conserved membrane protein of unknown function	38.1	38.4	20.1	2.8	0	0.39	0.63

NSCAC_0868	metR	HTH-type transcriptional regulator MetR	37.9	42	2.4	7.1	0	0.41	0.64
NSCAC_1369	bioA	Adenosylmethionine-8-amino-7-oxononanoate aminotransferase	37.9	108.3	15.5	11	1	0	0
NSCAC_0062		conserved protein of unknown function	37.8	55.4	25.1	5.4	0	0.93	0.97
NSCAC_0660		Oxidoreductase domain protein	37.8	73.9	32.4	18.1	1	0.25	0.47
NSCAC_0607		conserved membrane protein of unknown function	37.5	57	42.3	9.1	0	0.97	0.99
NSCAC_1331		putative Cellulose biosynthesis-like protein	37.3	69.5	17.6	34	0	0.38	0.61
NSCAC_1608		exported protein of unknown function	36.8	40.3	12.7	18.5	0	0.59	0.77
NSCAC_1059		conserved exported protein of unknown function	36.6	48.5	21.6	16.6	0	0.84	0.92
NSCAC_1393		conserved protein of unknown function	36.5	34.6	14.5	17.4	-1	0.37	0.6
NSCAC_1237		protein of unknown function	36.4	31.9	39.1	28.2	-1	0.6	0.77
NSCAC_0121		conserved protein of unknown function	36.3	54.2	9.7	28.1	0	0.74	0.86
NSCAC_0262	ccrMI M	Modification methylase CcrMI	36.2	72.3	3.5	10.2	1	0.08	0.22
NSCAC_1032		conserved protein of unknown function	36.1	169.5	14.7	28.5	2	0	0
NSCAC_0517	lspA	prolipoprotein signal peptidase (signal peptidase II)	36	54.6	14.1	30	0	0.74	0.86
NSCAC_1366		exported protein of unknown function	35.6	50.5	8.6	11	0	0.82	0.91
NSCAC_1312	rmlD	dTDP-4-dehydrorhamnose reductase subunit, NAD(P)-binding, of dTDP-L-rhamnose synthase	35.4	65.5	7.1	11.2	0	0.15	0.35
NSCAC_1016		conserved protein of unknown function	35.3	36.1	9.1	27	0	0.56	0.74
NSCAC_0676		protein of unknown function	35.2	29.8	24.4	17.7	-1	0.3	0.53
NSCAC_1690	pilT	twitching motility protein	35.2	37.3	11.6	7.7	0	0.4	0.64
NSCAC_0601		conserved membrane protein of unknown function	35.2	63.2	8	6.7	0	0.29	0.52
NSCAC_1541		Exodeoxyribonuclease III	34.7	62	24.9	22.9	0	0.45	0.67
NSCAC_1085		putative [NiFe]-hydrogenase, small subunit HyfI	34.6	77.2	24.2	24.9	1	0.11	0.28
NSCAC_1656	uvrD	DNA-dependent ATPase I and helicase II	34.5	33.9	4.9	5.3	0	0.21	0.43
NSCAC_1121		Type IV pilus biogenesis/stability protein PilW	34.5	51.6	12.2	16.2	0	0.81	0.9
NSCAC_1186	yfjG	conserved hypothetical protein	34.5	65.2	9.7	30	0	0.27	0.5
NSCAC_0011	priA	Primosome factor n' (replication factor Y)	34.4	73.9	13.7	7	1	0.09	0.23
NSCAC_0612		Hemin-degrading family protein	34.4	49.1	11.5	24.3	0	0.89	0.95
NSCAC_0534		conserved protein of unknown function	34.4	50.6	21.1	23.4	0	0.99	0.99
NSCAC_0802	nadC	quinolinate phosphoribosyltransferase	34.4	55.9	7.7	11.4	0	0.43	0.66
NSCAC_1447	bioH	Pimeloyl-[acyl-carrier protein] methyl ester esterase	34.2	42.3	18.4	6.5	0	0.83	0.91

NSCAC_1695		Major facilitator superfamily MFS_1	34.2	48.1	18	3.6	0	0.97	0.99
NSCAC_1451		Micrococcal nuclease (SNase-like)	34.2	102.8	7.7	17.5	1	0	0
NSCAC_0415		6-carboxy-5,6,7,8-tetrahydropterin synthase	34.2	76.3	23.4	9	1	0.22	0.44
NSCAC_0319	bsaA	Glutathione peroxidase homolog BsaA	34	36.6	19.8	28.1	0	0.69	0.83
NSCAC_1106	mutS	methyl-directed mismatch repair protein	34	34.5	4.2	4.9	0	0.3	0.53
NSCAC_0154	rlmB	23S rRNA (guanosine-2'-O-)-methyltransferase RlmB	34	50.3	13.6	3.8	0	0.78	0.89
NSCAC_1578	kdsC	3-deoxy-D-manno-octulosonate 8-phosphate phosphatase	33.8	107.3	11.7	79.6	1	0.02	0.09
NSCAC_0656		ABC transporter, ATP-binding protein	33.6	42.6	15.4	14.8	0	0.87	0.93
NSCAC_1187	yfjF	hypothetical protein	33.5	60.1	17.5	11.3	0	0.4	0.63
NSCAC_0308		Phosphate ABC transporter substrate-binding protein, PhoT family	33.4	58.1	18.9	14.1	0	0.38	0.62
NSCAC_1526	lpxK	lipid A 4'kinase	33.4	80.7	7.6	7.4	1	0.01	0.04
NSCAC_0152		Glutaredoxin 2	33.4	124.4	15.5	20.7	1	0	0.01
NSCAC_0482	ureG	Urease accessory protein UreG	33.3	220.1	28.7	50.3	2	0	0
NSCAC_0988		Putative di-heme cytochrome c	33.2	53.4	2.6	10	0	0.54	0.73
NSCAC_0818	cbbY P	Protein CbbY, plasmid	33.2	46.6	4.4	9.5	0	0.91	0.96
NSCAC_0828	rlmJ	Ribosomal RNA large subunit methyltransferase J	33.1	63.6	17.3	10.7	1	0.24	0.46
NSCAC_1310		Aminoglycoside phosphotransferase	33	58.1	7.5	16	0	0.26	0.49
NSCAC_0333		Sterol-binding domain protein	32.7	103	14.3	36.4	1	0	0.01
NSCAC_1605	ssb	Single-stranded DNA-binding protein	32.6	46.4	12.8	7.6	0	0.82	0.9
NSCAC_0445		Predicted proline hydroxylase	32.5	65	8.2	25.9	1	0.2	0.42
NSCAC_0657		Secretion protein HlyD	32.5	59.9	6.7	5.3	0	0.21	0.43
NSCAC_1146		ATP-binding region ATPase domain protein	32.4	73.1	2.9	7	1	0.02	0.08
NSCAC_0729		conserved membrane protein of unknown function	32.4	141.5	5.7	14.1	2	0	0
NSCAC_1363		Secretion ATPase, PEP-CTERM locus subfamily	32.4	55.9	27.6	9.4	0	0.47	0.68
NSCAC_1581		putative lipopolysaccharide transport protein B (ABC superfamily, atp_bind) (LptB)	32.3	102.3	9.8	20.5	1	0	0.01
NSCAC_0799	ispF	2C-methyl-D-erythritol 2,4-cyclodiphosphate synthase	32.3	64.4	20.4	26.3	1	0.33	0.56
NSCAC_0461	yheS	fused putative transporter subunits of ABC superfamily: ATP-binding components	32	61.4	4.9	1.4	0	0.14	0.34
NSCAC_1053	surA	Chaperone SurA	32	51.5	13.1	31	0	0.61	0.78
NSCAC_0253	ygiD	putative enzyme	31.6	87.1	15.6	5.3	1	0	0.01

NSCAC_0955		BFD domain protein (2Fe-2S)-binding domain protein	31.5	48.1	28.5	25.6	0	0.95	0.98
NSCAC_0742		Smr domain protein	31.5	49.8	3.1	11.1	0	0.58	0.76
NSCAC_0054	ybeZ	putative enzyme with nucleoside triphosphate hydrolase domain	31.4	105.5	7.2	13.5	1	0	0
NSCAC_1453	recG	ATP-dependent DNA helicase	31.3	48.2	9.1	10.4	0	0.58	0.76
NSCAC_1042	mboI AM	Modification methylase MboIA	31.3	49.2	8.7	14.2	0	0.66	0.81
NSCAC_0953	yjbQ	conserved hypothetical protein	31.2	33.3	22.6	24.1	0	0.66	0.81
NSCAC_0967		conserved membrane protein of unknown function	31.2	49.1	4.2	15.1	0	0.59	0.77
NSCAC_1700	dfp	fused 4'-phosphopantothenoylcysteine decarboxylase ; phosphopantothenoylcysteine synthetase, FMN-binding	31.1	59.9	14.8	15.4	1	0.2	0.42
NSCAC_0286		Type I restriction modification DNA specificity domain protein (fragment)	31	47	27	8.6	0	0.96	0.98
NSCAC_1603	uvrA	Excinuclease ABC, subunit A	30.9	34.4	4.8	3.8	0	0.47	0.68
NSCAC_1739		conserved protein of unknown function	30.9	82.9	16.8	29.5	1	0.06	0.18
NSCAC_1091		TonB-dependent receptor	30.8	35.9	8.1	5.2	0	0.62	0.78
NSCAC_0715		Outer membrane efflux protein	30.8	21.6	4.5	12	-1	0.08	0.23
NSCAC_0527	ffh	4.5S-RNP protein, GTP binding export factor, part of signal recognition particle with 4.5 RNA	30.6	64.6	6.7	10.1	1	0.06	0.17
NSCAC_1272	trpD	Anthranilate phosphoribosyltransferase	30.4	85.6	2.6	14.3	1	0	0
NSCAC_0130		Glycosyl transferase, group 2 family protein	30.4	39.6	17.3	7.6	0	0.97	0.99
NSCAC_0604		Outer membrane efflux protein	30.3	38.5	7.9	12.5	0	0.78	0.89
NSCAC_0842		protein of unknown function	30.3	54.7	14.5	5	0	0.29	0.52
NSCAC_1580		Lipopolysaccharide transport periplasmic protein LptA (modular protein)	30.2	63.9	12.4	44.3	1	0.25	0.47
NSCAC_1461		Efflux transporter, RND family, MFP subunit	30.1	33.9	12.7	9.1	0	0.57	0.76
NSCAC_0008		Formylmethanofuran dehydrogenase, subunit B	29.8	85.1	6.1	12.3	1	0	0.01
NSCAC_0826		conserved protein of unknown function	29.8	16.3	32.3	4.7	-1	0.25	0.48
NSCAC_1641		putative TonB-dependent receptor	29.7	65.7	8.2	1.7	1	0.06	0.18
NSCAC_0954		conserved protein of unknown function	29.5	29.3	20.2	14.6	0	0.53	0.73
NSCAC_0945		putative Phosphate-selective porin O and P	29.4	55.1	6.8	10.8	0	0.29	0.52
NSCAC_0164		Peptidase M48 Ste24p	29.3	79.7	9.9	24	1	0.02	0.09
NSCAC_0317		Phospholipid/glycerol acyltransferase	29.3	38.2	5	4.7	0	0.95	0.98
NSCAC_0068		Type IV pilus biogenesis protein PilQ	29.1	27.3	5.8	3.6	-1	0.25	0.47

NSCAC_1015		conserved protein of unknown function	28.8	44.2	10	22.8	0	0.72	0.85
NSCAC_1073		conserved protein of unknown function	28.7	31.9	17.9	14.4	0	0.65	0.81
NSCAC_0001	dnaN	DNA polymerase III subunit beta	28.6	34	22.6	16	0	0.84	0.92
NSCAC_1357		RimK domain protein ATP-grasp	28.6	55.2	8	1.1	1	0.18	0.4
NSCAC_0787		conserved protein of unknown function	28.5	31.9	30.7	19.3	0	0.91	0.96
NSCAC_1642		putative TonB-dependent siderophore receptor	28.5	59.6	1.7	6.4	1	0.07	0.2
NSCAC_1301		Periplasmic solute binding protein	28.5	101	21.1	27.1	1	0.01	0.03
NSCAC_0192		L-lysine 6-oxidase (fragment)	28.3	86	11.1	25.6	1	0.01	0.05
NSCAC_1760		Transposon Tn7 transposition protein TnsE (fragment)	28.3	34	15.1	10.4	0	0.73	0.86
NSCAC_1104	ygaD	conserved hypothetical protein	28.1	64.7	11.3	19.8	1	0.05	0.16
NSCAC_1128		Regulatory protein GntR HTH	28	31.3	10.2	7.3	0	0.52	0.73
NSCAC_1601	rsmB	16S rRNA m5C967 methyltransferase, S-adenosyl-L-methionine-dependent	28	44.8	5.6	16.9	0	0.66	0.81
NSCAC_1783	rpmH	50S ribosomal protein L34	28	31.3	48.5	23.5	-1	NA	NA
NSCAC_0869		Acid phosphatase	27.9	61.7	2.9	17.8	1	0.08	0.22
NSCAC_0193		conserved membrane protein of unknown function	27.9	45.6	4.4	14.2	0	0.53	0.73
NSCAC_0848		conserved protein of unknown function	27.7	40.3	24.3	11.5	0	0.84	0.92
NSCAC_1399	rluC	23S rRNA pseudouridylate synthase	27.6	54.1	13.6	38.6	0	0.46	0.67
NSCAC_0633		conserved membrane protein of unknown function	27.6	63.8	11.6	6.2	1	0.11	0.27
NSCAC_0506		conserved protein of unknown function	27.6	46.3	2.7	9.6	0	0.45	0.67
NSCAC_0015	grxC	glutaredoxin 3	27.3	36.6	14.1	19.9	0	0.95	0.98
NSCAC_0280		protein of unknown function	27.2	14.9	25.6	16.2	-1	0.44	0.67
NSCAC_0550	hrpA	ATP-dependent RNA helicase HrpA	27.1	28.2	2.1	6.6	0	0.39	0.62
NSCAC_0830	nth	endonuclease III DNA glycosylase/apyrimidinic (AP) lyase	27.1	42.3	10.7	7.1	0	0.71	0.84
NSCAC_0759		protein of unknown function	27	105.2	18	86.9	1	0.01	0.06
NSCAC_1378		Sel1 repeat family protein (fragment)	26.9	44.9	18.1	5.6	0	0.51	0.71
NSCAC_1320	coaD	pantetheine-phosphate adenylyltransferase	26.8	83.7	9.6	22.5	1	0.01	0.03
NSCAC_0463	apbE	Thiamine biosynthesis lipoprotein ApbE	26.8	47.8	8.9	17.5	0	0.36	0.59
NSCAC_0087		Stringent starvation protein B	26.4	54.7	2.8	10.2	1	0.12	0.29
NSCAC_0800		putative Hydrolase, alpha/beta fold family	26.4	64.1	9.2	21	1	0.04	0.13
NSCAC_0958	smc	Chromosome partition protein Smc	26.3	50.8	8.9	12.8	1	0.19	0.4

NSCAC_1388	polA	fused DNA polymerase I 5'->3' exonuclease ; 3'->5' polymerase ; 3'->5' exonuclease	26.3	39.9	4	7.4	0	0.7	0.84
NSCAC_0420		conserved protein of unknown function	25.9	94.5	18.1	29.5	1	0	0.01
NSCAC_1013	uvrB	excinuclease ABC subunit B (ATP-dependent DNA excision repair enzyme UvrAC)	25.4	45.7	9.4	1.9	0	0.26	0.49
NSCAC_1230		Guanine-specific ribonuclease N1 and T1	25.3	58.7	7.1	26.6	1	0.08	0.22
NSCAC_0101	tapC	Type IV pilus assembly protein TapC	25.2	61.6	6.6	6.3	1	0.02	0.09
NSCAC_0301	yidD	hypothetical protein	25.2	36	6.6	11.1	0	0.9	0.95
NSCAC_0451		PhoH family protein	25.2	38.6	7.9	18.3	0	0.73	0.86
NSCAC_0189	mutH	DNA mismatch repair protein MutH	25.2	69.9	9.5	20.2	1	0.01	0.04
NSCAC_1488		Rare lipoprotein A	25.1	42.9	21	18.6	0	0.54	0.74
NSCAC_0524	mutY	A/G-specific adenine glycosylase	25	39	3.1	7.2	0	0.57	0.75
NSCAC_0623		putative VTC domain-containing protein	24.9	80.8	6.3	23.1	1	0	0.01
NSCAC_1667		protein of unknown function	24.7	4.3	4.4	7.4	-3	0.04	0.13
NSCAC_0931		protein of unknown function	24.7	36.1	16.4	12.8	0	0.79	0.89
NSCAC_1611		protein of unknown function	24.6	69.4	27.8	61.8	1	0.61	0.78
NSCAC_0481	ureF	Urease accessory protein UreF	24.5	245.1	9.6	41.3	3	0	0
NSCAC_0947		conserved membrane protein of unknown function	24.4	43.4	9.6	10.1	0	0.48	0.69
NSCAC_0078		H4MPT-linked C1 transfer pathway protein	24.3	37.8	14.9	4.3	0	0.67	0.82
NSCAC_0050	dfrA	Dihydrofolate reductase	24.3	93.4	19.6	23.1	1	0.01	0.06
NSCAC_0038		protein of unknown function	24.3	5.3	21.2	9.2	-3	0.44	0.66
NSCAC_0005		Formylmethanofuran dehydrogenase, subunit C	24.3	37.5	4.2	5	0	0.66	0.81
NSCAC_0640		CRISPR-associated helicase Cas3 family	24.2	37.1	1.8	0.9	0	0.69	0.83
NSCAC_0789	glcF	glycolate oxidase iron-sulfur subunit	24.1	33.7	13.1	19.4	0	0.94	0.98
NSCAC_0485		Short chain amide porin	24.1	24.3	7.8	1.1	0	0.39	0.62
NSCAC_0255		Exopolysaccharide synthesis, ExoD	24	26	22.5	10.3	0	0.76	0.87
NSCAC_0184		Methyltransferase type 11	24	16.9	12.9	14	-1	0.27	0.5
NSCAC_0153	nfi	Endonuclease V	24	93.4	2	19.5	2	0	0
NSCAC_1430		conserved protein of unknown function	24	99.5	21.1	15.8	2	0.2	0.42
NSCAC_1056	sbcD	exonuclease, dsDNA, ATP-dependent	23.9	32.9	16.9	4.8	0	0.96	0.98
NSCAC_0998		protein of unknown function	23.9	12.5	25.3	13.2	-2	0.32	0.55
NSCAC_0457	mboI	Modification methylase MboIB	23.9	38.1	8.8	3.7	0	0.57	0.75

	BM								
NSCAC_1379		Sel1 repeat protein (modular protein)	23.8	24.1	1.9	13.3	0	0.51	0.71
NSCAC_1558	scpB	Segregation and condensation protein B homolog	23.6	75.1	18.3	16.9	1	0.03	0.11
NSCAC_1669	queC	7-cyano-7-deazaguanine synthase	23.6	83.1	14.3	4.9	1	0.01	0.04
NSCAC_1374		putative Glycosyl transferase group 1	23.4	45	18.2	13.8	1	0.38	0.61
NSCAC_0123		Glycosyl transferase family 2 (fragment)	23.1	42.1	6.2	10.1	0	0.35	0.58
NSCAC_0388		Peptidase M23	22.9	71.9	20.9	14.1	1	0.12	0.3
NSCAC_0441		conserved protein of unknown function	22.8	35.6	5.9	16.2	0	0.64	0.8
NSCAC_1058		conserved membrane protein of unknown function	22.6	63.4	5.8	9.8	1	0	0.02
NSCAC_1055		putative Nuclease SbcCD subunit C	22.6	37.1	5.9	9.8	0	0.54	0.74
NSCAC_0717		Integral membrane sensor signal transduction histidine kinase	22.5	61.2	20.5	14.5	1	0.12	0.29
NSCAC_0738		Permease YjgP/YjgQ family protein	22.5	38.5	9.6	18	0	0.51	0.72
NSCAC_0514	sfsA	Sugar fermentation stimulation protein homolog	22.5	51.4	1.8	6.7	1	0.06	0.18
NSCAC_1043		protein of unknown function	22.3	15.2	20.2	15.9	-1	0.52	0.72
NSCAC_0132	yjbN	tRNA-dihydrouridine synthase A	22.1	44.2	4.1	8.3	1	0.17	0.38
NSCAC_0978		conserved protein of unknown function	22.1	36.6	8.2	3	0	0.64	0.8
NSCAC_0263		Carboxymuconolactone decarboxylase family protein	21.5	47	18.7	17.3	1	0.3	0.53
NSCAC_1360	argA	Amino-acid acetyltransferase	21.5	38.9	19.4	9.4	0	0.53	0.73
NSCAC_1392		protein of unknown function	21.4	16.2	37	16.7	-1	0.84	0.92
NSCAC_0809		membrane protein of unknown function	21.2	31	14	14.9	0	0.83	0.91
NSCAC_1072		conserved protein of unknown function	21.1	48.1	24.1	24.5	1	0.3	0.53
NSCAC_0284		protein of unknown function	21	37.5	7.3	26.2	0	0.54	0.73
NSCAC_0223		conserved protein of unknown function	20.9	65	24.6	63.1	1	0.43	0.66
NSCAC_0912		Aminodeoxychorismate lyase	20.8	65.1	13.2	17.2	1	0.01	0.06
NSCAC_1633	aat	leucyl/phenylalanyl-tRNA-protein transferase	20.8	36.7	3.7	4.8	0	0.43	0.66
NSCAC_0834	rnfC	Electron transport complex protein RnfC	20.7	55.9	2.7	9.6	1	0.01	0.06
NSCAC_1174		Acriflavin resistance protein	20.6	33	5.9	6.3	0	0.62	0.79
NSCAC_1514	nadD	putative nicotinate-nucleotide adenylyltransferase	20.1	26.6	10.8	10.2	0	0.97	0.99
NSCAC_0631		Auxin Efflux Carrier	19.9	47.2	12.8	9	1	0.13	0.32
NSCAC_1446	bioC	Malonyl-[acyl-carrier protein] O-methyltransferase	19.8	45.7	18.8	16.8	1	0.36	0.6
NSCAC_0066		Pilus assembly protein PilO	19.7	20.3	9.1	7.1	0	0.59	0.77

NSCAC_0149		General secretion pathway protein H (fragment)	19.7	53.8	5.2	23.4	1	0.04	0.13
NSCAC_0655		conserved membrane protein of unknown function	19.7	47	4.7	14.4	1	0.09	0.24
NSCAC_0096		conserved protein of unknown function	19.6	62	20.9	23.6	1	0.15	0.35
NSCAC_0487		protein of unknown function	19.6	42.4	11.5	11.6	1	0.33	0.57
NSCAC_0637		Bifunctional deaminase-reductase domain protein	19.5	53.5	23.2	6.6	1	0.12	0.3
NSCAC_0112		General secretion pathway protein M	19.4	87.4	8.9	21.3	2	0	0
NSCAC_1098		conserved protein of unknown function	19.4	34.9	6.5	10.8	0	0.44	0.67
NSCAC_1645		protein of unknown function	19.2	109.8	14.9	12.2	2	0	0
NSCAC_1559	scpA	Segregation and condensation protein A	19.2	60	17.2	6.1	1	0.03	0.1
NSCAC_0521		protein of unknown function	19.1	75.9	12.3	9.8	2	0	0.01
NSCAC_1424	ftsL	Cell division protein FtsL	18.9	11.6	19.2	6.6	-1	0.36	0.6
NSCAC_0641	cas	CRISPR-associated endonuclease Cas1	18.9	44.3	5.3	2.8	1	0.06	0.18
NSCAC_0750	queA	S-adenosylmethionine:tRNA ribosyltransferase-isomerase	18.9	27.3	7	9.2	0	0.84	0.92
NSCAC_0076		Aspartate/glutamate/uridylate kinase	18.8	33.4	7.9	7.5	0	0.55	0.74
NSCAC_1021		protein of unknown function	18.8	8.6	16.4	7.5	-2	0.33	0.56
NSCAC_0512		Glycosyl transferase family 2	18.7	35.2	5.8	11.4	0	0.37	0.61
NSCAC_1362		HAD-superfamily subfamily IB, PSPase-like protein	18.6	47	4.9	4.8	1	0.03	0.12
NSCAC_1370		protein of unknown function	18.5	78.7	16.5	41.2	2	0.04	0.13
NSCAC_0835	rsxB	putative iron-sulfur protein	18.3	24.5	16	20.4	0	1	1
NSCAC_1034		conserved protein of unknown function	18	40.4	6.1	9.7	1	0.17	0.39
NSCAC_0507		conserved protein of unknown function	18	20.2	24.6	10.8	0	0.89	0.95
NSCAC_1375		Glycosyl transferase family 9	17.9	42.8	10.6	16.7	1	0.15	0.34
NSCAC_1396	kdsB	3-deoxy-manno-octulosonate cytidylyltransferase	17.8	43.4	9.3	9.1	1	0.1	0.25
NSCAC_0186		protein of unknown function	17.8	44.1	16.2	26.5	1	0.44	0.66
NSCAC_1475	bamD	Outer membrane protein assembly factor BamD	17.8	29.4	16.9	9.5	0	0.82	0.91
NSCAC_0674		protein of unknown function	17.8	18.4	19	10.9	0	0.81	0.9
NSCAC_0316	trpH	Protein TrpH	17.8	51.8	16.6	4.8	1	0.21	0.43
NSCAC_0287		Type I restriction enzyme EcoprrI M protein (fragment)	17.6	6.8	15.4	0.8	-2	0.21	0.43
NSCAC_0289		protein of unknown function	17.6	29.3	4.4	10.8	0	0.64	0.8
NSCAC_1384		Glycosyl transferase group 1	17.5	25.8	12.4	11.2	0	0.8	0.9
NSCAC_0642		conserved protein of unknown function	17.5	19.1	3.3	7.3	0	0.61	0.78

NSCAC_0986		Peptidase S8 and S53 subtilisin kexin sedolisin	17.4	35.5	10.3	9.5	1	0.29	0.52
NSCAC_0129		3-beta hydroxysteroid dehydrogenase/isomerase family	17.3	33.7	16.3	8.3	1	0.53	0.73
NSCAC_1353	yifB	putative bifunctional enzyme and transcriptional regulator	17.2	39.3	3.2	0.3	1	0.1	0.25
NSCAC_1071		membrane protein of unknown function	17.1	23.8	3	5.4	0	1	1
NSCAC_1133	folK	2-amino-4-hydroxy-6-hydroxymethyldihyropteridine pyrophosphokinase	16.8	38.9	15.8	9.5	1	0.34	0.58
NSCAC_1314	rmlA	dTDP-glucose pyrophosphorylase (glucose-1-phosphate thymidylyltransferase)	16.8	26.9	9.1	9.8	0	0.63	0.79
NSCAC_1535		conserved protein of unknown function	16.7	87.5	29	26.5	2	0.36	0.6
NSCAC_0697		conserved protein of unknown function	16.6	76.6	4.4	32.2	2	0	0
NSCAC_1096	bepIM	Modification methylase BepI	16.6	26.1	7.2	10.2	0	0.7	0.84
NSCAC_0080		Histidine biosynthesis protein	16.6	64.7	7.6	15	2	0	0.01
NSCAC_1041		protein of unknown function	16.6	29.2	4.4	17.2	0	0.59	0.77
NSCAC_0077	pabB	Aminodeoxychorismate synthase component 1	16.4	40.2	8	5.6	1	0.09	0.24
NSCAC_1427	yraL	putative methyltransferase	16.3	21	2.7	6.8	0	0.88	0.94
NSCAC_0890	fumC	Fumarate hydratase class II	16.3	28.5	7.9	7.8	0	0.54	0.73
NSCAC_0483		conserved protein of unknown function	16.3	129.3	7.7	34.9	3	0	0
NSCAC_1660		protein of unknown function	16.3	33.4	10.1	10.5	1	0.29	0.52
NSCAC_1031		conserved protein of unknown function	16.2	82.3	28	18.3	2	0.37	0.6
NSCAC_0987		conserved protein of unknown function	16.1	24.3	6.8	7.6	0	0.79	0.89
NSCAC_0966		conserved protein of unknown function	16	90.9	13.9	25.8	2	0.13	0.31
NSCAC_0804		putative membrane protein	16	35.5	5	5.1	1	0.19	0.4
NSCAC_0102	pilB	Type 4 fimbrial assembly protein PilB	16	22.5	3.4	3.9	0	0.96	0.98
NSCAC_0563		conserved protein of unknown function	15.9	34.9	6.7	8.1	1	0.23	0.46
NSCAC_0603		Efflux transporter, RND family, MFP subunit	15.9	19.6	3.5	4.5	0	0.79	0.89
NSCAC_1534		exported protein of unknown function	15.4	21.8	9.3	10.7	0	0.98	0.99
NSCAC_0682		HAD-superfamily hydrolase subfamily IA, variant 3	14.9	25.7	1.6	15.8	0	0.63	0.79
NSCAC_1173		RND efflux system, outer membrane lipoprotein, NodT family	14.7	18.3	5.2	4.2	0	0.85	0.92
NSCAC_1062		protein of unknown function	14.7	3.8	13.3	6.6	-2	0.28	0.51
NSCAC_0876		conserved protein of unknown function	14.6	55	7.2	34.1	1	0.01	0.05
NSCAC_0901	argS	Arginyl-tRNA synthetase	14.5	28.6	1.9	8.1	1	0.38	0.61
NSCAC_1617	prmC	Release factor glutamine methyltransferase	14.5	21.9	1.9	5	0	0.85	0.92

NSCAC_0716	tctD	Transcriptional regulatory protein tctD	14.4	33.8	9.2	16.8	1	0.35	0.58
NSCAC_0867		TonB-dependent heme/hemoglobin receptor family protein/TonB-dependent hemoglobin/transferrin/lactoferrin receptor family protein	14.3	25.2	3.7	8.5	0	0.54	0.73
NSCAC_1371	yfiH	Laccase domain protein YfiH	14.2	32.6	13.6	1.5	1	0.44	0.66
NSCAC_0559		conserved protein of unknown function	14	37	16.1	13.5	1	0.31	0.55
NSCAC_1359		GCN5-related N-acetyltransferase (fragment)	13.9	32.1	3.6	12.8	1	0.19	0.41
NSCAC_1027		conserved protein of unknown function	13.9	10.7	12.5	9.5	-1	0.63	0.79
NSCAC_1607	sspIM	M.SspI	13.6	16.3	3.5	1	0	0.77	0.88
NSCAC_0296	ada	Bifunctional transcriptional activator/DNA repair enzyme Ada [Includes: Methylphosphotriester-DNA--protein-cysteine S-methyltransferase ; Methylated-DNA--protein-cysteine methyltransferase]	13.4	42.3	3.8	17.9	1	0.04	0.14
NSCAC_0500		Arginine decarboxylase	13.4	44.8	23.2	22.6	1	0.56	0.75
NSCAC_1432		exported protein of unknown function	13	19.3	15.4	13.3	0	0.87	0.93
NSCAC_1683	glnE	Glutamate-ammonia-ligase adenylyltransferase	13	22.4	5.4	2.7	0	0.58	0.76
NSCAC_0638		conserved protein of unknown function	13	31.6	22.4	28.7	1	0.78	0.89
NSCAC_0464		conserved protein of unknown function	12.9	24.3	11.3	14.4	0	0.78	0.88
NSCAC_1044		protein of unknown function	12.9	0	22.3	0	-7	0.05	NA
NSCAC_1329		protein of unknown function	12.9	10.4	22.3	18	-1	0.74	0.86
NSCAC_0215		protein of unknown function	12.8	16.6	11.3	11.8	0	0.95	0.98
NSCAC_0097		Mutator MutT protein	12.7	29.5	7.1	11	1	0.24	0.46
NSCAC_0003		conserved protein of unknown function	12.7	9.9	11.2	11.2	-1	0.61	0.78
NSCAC_0433		protein of unknown function	12.7	51.1	11.1	6.4	1	0.1	0.26
NSCAC_1619	cca	Multifunctional CCA protein [Includes: CCA-adding enzyme ; 2'-nucleotidase ; 2',3'-cyclic phosphodiesterase ; Phosphatase]	12.6	57.7	3.6	7.7	2	0	0
NSCAC_1668		conserved protein of unknown function	12.6	19.7	5.7	9.6	0	0.83	0.91
NSCAC_0740		protein of unknown function	12.4	5.1	21.5	8.8	-2	0.55	0.74
NSCAC_0168	waaA	3-deoxy-D-manno-octulosonic acid transferase	12.2	33.4	11.1	10.9	1	0.27	0.5
NSCAC_0435		protein of unknown function	12.2	0	21	0	-6	0.1	NA
NSCAC_1481	yjeK	putative lysine aminomutase	11.9	31.9	7.5	4	1	0.14	0.34
NSCAC_0439		protein of unknown function	11.9	5.7	20.6	9.8	-1	0.65	0.8
NSCAC_0051		conserved protein of unknown function	11.8	84	4.8	6.4	2	0	0
NSCAC_0648		conserved protein of unknown function	11.7	6.9	10.3	6.1	-1	0.57	0.75

NSCAC_0281	rbcR	RuBisCO operon transcriptional regulator	11.4	31.1	13.1	11.1	1	0.33	0.56
NSCAC_0909		DNA-directed DNA polymerase	11.2	24	6.1	7.1	1	0.42	0.65
NSCAC_0810		membrane protein of unknown function	11.2	34.5	10.5	4	1	0.19	0.4
NSCAC_0134	DDC	Aromatic-L-amino-acid decarboxylase	11	20.3	0.9	3.7	0	0.53	0.73
NSCAC_0290		N-acetylmuramoyl-L-alanine amidase	10.9	17.4	3.4	3.3	0	0.76	0.87
NSCAC_0513		Glycosyl transferase group 1	10.6	35.6	8.6	17.1	1	0.14	0.33
NSCAC_0462	msrB	methionine sulfoxide reductase B	10.6	21.2	9.9	14.1	1	0.59	0.77
NSCAC_1450		Competence protein F	10.4	26.1	10	3.7	1	0.35	0.58
NSCAC_0067		putative Pilus assembly protein PilP	10.3	43	10.5	13.2	2	0.08	0.23
NSCAC_0647		protein of unknown function	10.3	4.2	17.8	7.3	-2	0.59	NA
NSCAC_0006	ffsA	Formylmethanofuran--tetrahydromethanopterin formyltransferase	9.9	22	9.5	5.3	1	0.61	0.78
NSCAC_1478		protein of unknown function	9.6	5.5	16.6	9.6	-1	0.77	NA
NSCAC_1394		protein of unknown function	9.5	7.5	16.5	6.7	-1	0.68	0.83
NSCAC_0645		CRISPR-associated protein, Csy3 family	9.5	18.9	7.9	7	1	0.52	0.72
NSCAC_0007	fhcA	Formyltransferase/hydrolase complex Fhc subunit A	9.5	34.7	2.4	5.4	1	0.02	0.07
NSCAC_0099	coaE	dephospho-CoA kinase	9.4	24.1	2.5	2.8	1	0.16	0.37
NSCAC_0892		Heptaprenyl diphosphate synthase component I (fragment)	9.4	18.1	16.2	9.7	1	0.74	0.86
NSCAC_0744		protein of unknown function	9.4	5.4	16.2	9.4	-1	0.76	NA
NSCAC_1046		Amidophosphoribosyltransferase (modular protein)	8.8	22.9	8.3	19.2	1	0.43	0.66
NSCAC_1438		protein of unknown function	8.8	30.5	15.2	17.8	2	0.32	0.55
NSCAC_1367	rsmE	Ribosomal RNA small subunit methyltransferase E	8.7	11.2	4	7.2	0	0.91	0.97
NSCAC_0081	mptN	Alpha-L-glutamate ligase	8.7	17.6	2.2	9.7	1	0.51	0.71
NSCAC_0756		protein of unknown function	8.6	0	15	0	-6	0.14	NA
NSCAC_0644		protein of unknown function	8.6	0	14.9	0	-6	0.11	NA
NSCAC_0493		conserved exported protein of unknown function	8.5	10.5	14.7	10.6	0	0.98	0.99
NSCAC_1509		Aminoglycoside N(6')-acetyltransferase type 1 (fragment)	8.5	0	14.7	0	-6	0.14	NA
NSCAC_0092		putative regulatory protein, FmdB family	8.2	33.8	14.1	17.8	2	0.23	0.46
NSCAC_1255		conserved protein of unknown function	8.1	17.3	7.1	15.3	1	0.75	0.87
NSCAC_1506	tusA	Sulfurtransferase TusA homolog	8.1	25	14	14.2	1	0.54	0.73
NSCAC_0605		conserved protein of unknown function	7.8	23.5	13.5	5.7	1	0.41	0.64
NSCAC_0165	tusA	Sulfurtransferase TusA homolog	7.6	38.6	13.1	8	2	0.23	0.46

NSCAC_0458		protein of unknown function	7.6	19.2	6.7	2.5	1	0.39	0.62
NSCAC_0214		membrane protein of unknown function	7.4	34.5	6.5	24.5	2	0.13	0.31
NSCAC_1527	ycaR	conserved hypothetical protein	7.3	33.7	12.7	21.4	2	0.2	0.41
NSCAC_0518		Pilus assembly protein	7.2	19.7	6.3	9	1	0.39	0.62
NSCAC_0643		conserved protein of unknown function	7.1	13.9	3.2	3.4	1	0.58	0.76
NSCAC_1093		protein of unknown function	7	3.3	12.2	5.8	-1	0.71	NA
NSCAC_0260		conserved protein of unknown function	7	23	6.1	1.1	1	0.19	0.41
NSCAC_1761		protein of unknown function	6.6	10.5	11.4	4.8	0	0.97	0.99
NSCAC_0976		conserved protein of unknown function	6.5	25.7	11.2	13.1	2	0.28	0.51
NSCAC_0897		protein of unknown function	5.9	21.9	10.2	19.1	2	0.47	0.69
NSCAC_1405		conserved protein of unknown function	5.6	62.9	9.7	13.2	3	0.1	0.25
NSCAC_0079		conserved protein of unknown function	5.6	14.9	5.6	5.4	1	0.44	0.66
NSCAC_0520		putative Fimbrial biogenesis protein	5.4	39	4.7	20.9	2	0.02	0.08
NSCAC_0636		conserved exported protein of unknown function	5.4	42	4.7	22	2	0.01	0.05
NSCAC_1084		Cytochrome c class I (fragment)	5.1	41.9	8.9	17.7	3	0.04	0.14
NSCAC_1229		conserved protein of unknown function	5	26.2	8.7	19.6	2	0.19	0.41
NSCAC_1684		protein of unknown function	4.9	15.9	8.5	14.8	1	0.58	0.76
NSCAC_0082		conserved protein of unknown function	4.9	14.2	5.2	10.2	1	0.47	NA
NSCAC_0634		HAD-superfamily hydrolase, subfamily IA, variant 3	4.9	30.9	4.4	15.7	2	0.05	0.16
NSCAC_1462	macB	fragment of fused macrolide transporter subunits of ABC superfamily: ATP-binding component ; membrane component (part 1)	4.6	32.3	4.1	7.2	2	0.02	0.08
NSCAC_0065		Type 4 fimbrial biogenesis protein PilN	3.2	15.4	5.6	13.4	2	0.45	NA
NSCAC_0041		HlyD family multidrug efflux protein	1.9	22.8	3.2	5.6	3	0.04	0.13
NSCAC_0023		protein of unknown function	0	0	0	0	NA	NA	NA
NSCAC_0053		protein of unknown function	0	0	0	0	NA	NA	NA
NSCAC_0071		Nucleic acid-binding domain protein (fragment)	0	12.4	0	6.7	6	0.03	NA
NSCAC_0072		protein of unknown function	0	0	0	0	NA	NA	NA
NSCAC_0073		protein of unknown function	0	0	0	0	NA	NA	NA
NSCAC_0135		protein of unknown function	0	0	0	0	NA	NA	NA
NSCAC_0176		protein of unknown function	0	0	0	0	NA	NA	NA
NSCAC_0180		protein of unknown function	0	0	0	0	NA	NA	NA

NSCAC_0181		protein of unknown function	0	0	0	0	NA	NA	NA
NSCAC_0182		protein of unknown function	0	25.3	0	29.8	7	0	0.02
NSCAC_0183		protein of unknown function	0	0	0	0	NA	NA	NA
NSCAC_0185		protein of unknown function	0	0	0	0	NA	NA	NA
NSCAC_0216		protein of unknown function	0	0	0	0	NA	NA	NA
NSCAC_0217		protein of unknown function	0	0	0	0	NA	NA	NA
NSCAC_0218		membrane protein of unknown function	0	3.9	0	6.8	4	0.3	NA
NSCAC_0285		protein of unknown function	0	5.3	0	9.2	4	0.25	NA
NSCAC_0393		protein of unknown function	0	19.1	0	17	6	0.02	NA
NSCAC_0394		Holo-[acyl-carrier-protein] synthase (fragment)	0	5.2	0	9	4	0.28	NA
NSCAC_0428		protein of unknown function	0	0	0	0	NA	NA	NA
NSCAC_0430		protein of unknown function	0	0	0	0	NA	NA	NA
NSCAC_0434		protein of unknown function	0	11.3	0	10	5	0.14	NA
NSCAC_0436		protein of unknown function	0	9.1	0	15.8	5	0.2	NA
NSCAC_0465		protein of unknown function	0	0	0	0	NA	NA	NA
NSCAC_0474		conserved protein of unknown function	0	31.3	0	9.7	7	0	0
NSCAC_0492		protein of unknown function	0	0	0	0	NA	NA	NA
NSCAC_0495		protein of unknown function	0	0	0	0	NA	NA	NA
NSCAC_0519		putative Fimbrial protein pilin	0	43.8	0	26.3	7	0	0
NSCAC_0649		conserved protein of unknown function	0	0	0	0	NA	NA	NA
NSCAC_0650		protein of unknown function	0	0	0	0	NA	NA	NA
NSCAC_0651		conserved protein of unknown function	0	3.7	0	6.4	4	0.34	NA
NSCAC_0652		protein of unknown function	0	0	0	0	NA	NA	NA
NSCAC_0672		protein of unknown function	0	0	0	0	NA	NA	NA
NSCAC_0675		protein of unknown function	0	0	0	0	NA	NA	NA
NSCAC_0684		protein of unknown function	0	0	0	0	NA	NA	NA
NSCAC_0687		protein of unknown function	0	0	0	0	NA	NA	NA
NSCAC_0689		protein of unknown function	0	19.3	0	23.1	6	0.02	NA
NSCAC_0690		protein of unknown function	0	8.7	0	7.7	5	0.19	NA
NSCAC_0692		conserved protein of unknown function	0	0	0	0	NA	NA	NA
NSCAC_0694		protein of unknown function	0	0	0	0	NA	NA	NA

NSCAC_0698		protein of unknown function	0	4.2	0	7.3	4	0.31	NA
NSCAC_0699		protein of unknown function	0	40	0	51.8	7	0	0
NSCAC_0700		protein of unknown function	0	0	0	0	NA	NA	NA
NSCAC_0748		Putative cytochrome c, class I	0	0	0	0	NA	NA	NA
NSCAC_0820		conserved exported protein of unknown function	0	753.1	0	47.6	1	0	0
NSCAC_0823		protein of unknown function	0	0	0	0	NA	NA	NA
NSCAC_0824		protein of unknown function	0	9.1	0	8	5	0.18	NA
NSCAC_0827		conserved protein of unknown function	0	0	0	0	NA	NA	NA
NSCAC_0845		protein of unknown function	0	0	0	0	NA	NA	NA
NSCAC_0850		conserved protein of unknown function	0	0	0	0	NA	NA	NA
NSCAC_0851		protein of unknown function	0	0	0	0	NA	NA	NA
NSCAC_0899		protein of unknown function	0	0	0	0	NA	NA	NA
NSCAC_0902		protein of unknown function	0	0	0	0	NA	NA	NA
NSCAC_0932		protein of unknown function	0	0	0	0	NA	NA	NA
NSCAC_0946		protein of unknown function	0	0	0	0	NA	NA	NA
NSCAC_0991		protein of unknown function	0	0	0	0	NA	NA	NA
NSCAC_1001		protein of unknown function	0	0	0	0	NA	NA	NA
NSCAC_1019		protein of unknown function	0	448.7	0	33.3	0	0.06	0.18
NSCAC_1022		protein of unknown function	0	0	0	0	NA	NA	NA
NSCAC_1040		protein of unknown function	0	0	0	0	NA	NA	NA
NSCAC_1048		protein of unknown function	0	22.9	0	28.2	7	0.01	0.05
NSCAC_1057		protein of unknown function	0	0	0	0	NA	NA	NA
NSCAC_1099		protein of unknown function	0	8.4	0	4.8	5	0.15	NA
NSCAC_1139		protein of unknown function	0	0	0	0	NA	NA	NA
NSCAC_1140		protein of unknown function	0	0	0	0	NA	NA	NA
NSCAC_1150		protein of unknown function	0	0	0	0	NA	NA	NA
NSCAC_1180		protein of unknown function	0	0	0	0	NA	NA	NA
NSCAC_1183		protein of unknown function	0	0	0	0	NA	NA	NA
NSCAC_1184		conserved protein of unknown function	0	3.4	0	6	4	0.33	NA
NSCAC_1225		protein of unknown function	0	0	0	0	NA	NA	NA
NSCAC_1249		protein of unknown function	0	0	0	0	NA	NA	NA

NSCAC_1380		protein of unknown function	0	0	0	0	NA	NA	NA
NSCAC_1395		protein of unknown function	0	9.8	0	16.9	5	0.17	NA
NSCAC_1426		conserved protein of unknown function	0	0	0	0	NA	NA	NA
NSCAC_1428		protein of unknown function	0	0	0	0	NA	NA	NA
NSCAC_1441		protein of unknown function	0	12.3	0	3.8	6	0.03	NA
NSCAC_1442		protein of unknown function	0	4.3	0	7.5	4	0.28	NA
NSCAC_1460		protein of unknown function	0	0	0	0	NA	NA	NA
NSCAC_1498		protein of unknown function	0	0	0	0	NA	NA	NA
NSCAC_1510		GCN5-related N-acetyltransferase (fragment)	0	0	0	0	NA	NA	NA
NSCAC_1532		protein of unknown function	0	0	0	0	NA	NA	NA
NSCAC_1533		protein of unknown function	0	4	0	6.9	4	0.33	NA
NSCAC_1566		protein of unknown function	0	4.3	0	7.4	4	0.31	NA
NSCAC_1616		protein of unknown function	0	0	0	0	NA	NA	NA
NSCAC_1621		protein of unknown function	0	5.9	0	10.2	5	0.24	NA
NSCAC_1640		protein of unknown function	0	43.4	0	47.4	7	0.06	0.18
NSCAC_1694	yggU	conserved hypothetical protein	0	14.3	0	5.7	6	0.01	NA
NSCAC_1705		protein of unknown function	0	0	0	0	NA	NA	NA
NSCAC_1762		protein of unknown function	0	0	0	0	NA	NA	NA
NSCAC_1763		Transposon Tn7 transposition protein TnsA (fragment)	0	6.9	0	6.6	5	0.22	NA
NSCAC_1764		protein of unknown function	0	18	0	15.7	6	0.02	NA
